# Greedy routing optimisation in hyperbolic networks

**DOI:** 10.1038/s41598-023-50244-8

**Published:** 2023-12-27

**Authors:** Bendegúz Sulyok, Gergely Palla

**Affiliations:** 1 Department of Biological Physics, Eötvös Loránd University, Pázmány P. stny. 1/A, 1117 Budapest, Hungary; 2 Data-Driven Health Division of National Laboratory for Health Security, Health Services Management Training Centre, Semmelweis University, Kútvölgyi út 2, 1125 Budapest, Hungary

**Keywords:** Complex networks, Statistical physics

## Abstract

Finding the optimal embedding of networks into low-dimensional hyperbolic spaces is a challenge that received considerable interest in recent years, with several different approaches proposed in the literature. In general, these methods take advantage of the exponentially growing volume of the hyperbolic space as a function of the radius from the origin, allowing a (roughly) uniform spatial distribution of the nodes even for scale-free small-world networks, where the connection probability between pairs decays with hyperbolic distance. One of the motivations behind hyperbolic embedding is that optimal placement of the nodes in a hyperbolic space is widely thought to enable efficient navigation on top of the network. According to that, one of the measures that can be used to quantify the quality of different embeddings is given by the fraction of successful greedy paths following a simple navigation protocol based on the hyperbolic coordinates. In the present work, we develop an optimisation scheme for this score in the native disk representation of the hyperbolic space. This optimisation algorithm can be either used as an embedding method alone, or it can be applied to improve this score for embeddings obtained from other methods. According to our tests on synthetic and real networks, the proposed optimisation can considerably enhance the success rate of greedy paths in several cases, improving the given embedding from the point of view of navigability.

## Introduction

Network theory has become ubiquitous in the analysis of complex systems ranging from molecular interactions up to the level of the global economy or the entire society^1–3^. One of the very notable approaches in modelling the statistical features of the web of connections between parts of a complex system is given by hyperbolic networks^4–10^, where nodes are placed in a hyperbolic space and are connected according to a probability that is decreasing with the hyperbolic distance. These models can generate scale-free, highly clustered and small-world random graphs, reproducing some of the most important universal features of networks representing complex systems. In addition, the often observed modular structure of real-world networks^11–13^ can also be easily grasped by these approaches^14–17^.

Probably the most well-known hyperbolic model is the popularity-similarity optimisation (PSO) model^5^, where the nodes are introduced one by one at logarithmically increasing radial coordinates and random uniform angular coordinates in the native disk representation of the 2d hyperbolic space, and the connection probability between nodes is decaying according to a Fermi-function, depending on the hyperbolic distance and a temperature-like parameter. Another very notable approach is provided by the random hyperbolic graph^4^, where the network is static and is obtained by placing the nodes at random onto the native disk and connecting them according to a connection probability that is decaying with the hyperbolic distance in a similar fashion as mentioned above. Several variations and generalisations of these seminal models were proposed over the years by e.g., adding slight modifications to the linking procedures^6–9^, extending the approaches to higher dimensions^18–21^, or incorporating tunable community structures^14,15^.

The success of hyperbolic models inherently brought with itself the interest towards the inverse problem as well, where the task is to find an optimal arrangement of the nodes in the hyperbolic space based on a given input network data. The first ideas about the hyperbolic embedding of networks appeared in Ref. ^22^, which was followed by the development of various different approaches later. A very natural idea that comes up is likelihood optimisation with respect to a hyperbolic model, as implemented in Hypermap ^6^, an early method for minimising a logarithmic loss function based on the assumption that the input network was generated according to an extended version of the PSO-model. Another popular option is to apply dimension reduction techniques on matrices representing the distance relations between the nodes, leading to model-independent embeddings such as the Laplacian eigenmaps approach ^23^, the family of coalescent embeddings ^24^, and the Hydra method ^25^. Dimension reduction and optimisation techniques can be also combined as proposed in the case of the Mercator method ^8,26^ or when fusing the Laplacian embedding approach with E-PSO model-based optimisation ^27^, or when applying a local likelihood optimisation to the output of a coalescent embedding algorithm ^9^. Hyperbolic embeddings were also extended to bipartite networks ^28^, whereas the recent generalisations of hyperbolic embedding approaches include methods for dealing with directed networks ^29^ and the embedding of multiplex networks as well ^30^.

The quality of an embedding can be quantified according to several different measures, e.g., in the case of likelihood optimisation, the lowest achieved value of the loss function is a straightforward simple quality indicator. Another approach for measuring the quality of hyperbolic embeddings is focusing on greedy routing, that was initially motivated by the assumption that the majority of the links in a hyperbolic network tend to follow the geodesic lines ^4^, enabling an efficient routing based on the hyperbolic coordinates ^31^. The idea of greedy routing on networks embedded in a geometric space in general goes back to the pioneering work by Kleinberg ^32^, considering a navigation protocol where we always proceed to the neighbour that is the closest to the destination node according to the distance defined in the given geometric space. Naturally, for networks embedded in a hyperbolic space, this greedy routing path is based on the hyperbolic distance between the nodes ^31^. The routing stops either when the target has been reached, or when hoping onto an already visited node, meaning that instead of reaching the target the path ends in a cycle and the greedy routing is unsuccessful ^22,31^.

Although hyperbolic networks are usually considered to be very suitable for greedy routing ^4,6,22,24,31^, still, in most cases, the greedy paths are not 100% successful in reaching the target, as pointed out by a recent study also raising some concerns regarding the widely believed high congruence between hyperbolic networks and their underlying space^33^. Motivated by that, here we develop an optimisation procedure for increasing the efficiency of greedy routing on the native disk representation of the hyperbolic space, and apply it to both PSO networks generated in the hyperbolic space itself and real networks embedded in the native disk. Besides the study of the achievable increase in the greedy routing efficiency we also examine how the optimisation affects further properties of the embedding.

## Results

### Preliminaries

The efficiency of greedy routing is affected by both the fraction of successful greedy paths and the length of these successful paths. A quantity for quantifying the greedy routing efficiency that grasps both of these factors is given by the greedy routing score ^24^

1 $$\begin{aligned} \textrm{GR}(\{r_i,\theta _i\})= \frac{1}{N(N-1)}\cdot \sum \limits _{s\in N}\,\,\sum \limits _{t\in N, t\ne s}\frac{\ell _{s\rightarrow t}^{\mathrm {(SP)}}}{\ell _{s\rightarrow t}^{\mathrm {(GR)}}}, \end{aligned}$$

where $(\{r_i,\theta _i\})$ refers to the actual node positions given with the help of radial and angular coordinates, *N* is the number of nodes and the summations take into account all possible source-target pairs with $\ell _{s\rightarrow t}^{\mathrm {(SP)}}$ and $\ell _{s\rightarrow t}^{\mathrm {(GR)}}$ denoting the length of shortest and greedy paths respectively (where $\ell _{s\rightarrow t}^{\mathrm {(GR)}}=\infty$ if the routing is unsuccessful). (The details of the calculation of the above measure are described in detail in the Methods).

Unsuccessful paths, where the routing protocol enters a cycle instead of reaching the target usually cause a more serious problem compared to the issues raised by the fact that the length of the successful paths can be sub-optimal. Hence, the main priority of our optimisation algorithm is to increase the fraction of successful paths. A quantity focusing only on the success of the paths was introduced in the literature as the success ratio, $p_{\textrm{s}}$, corresponding to the fraction of the successful greedy paths when all possible source and target pairs are considered^31^. This can be written in a similar fashion to (1) in the form of

2 $$\begin{aligned} p_{\textrm{s}}(\{r_i, \theta _i\}) = \frac{1}{N (N-1)} \sum _{s \in V} \sum _{\begin{array}{c} t \in V \\ t \ne s \end{array}} \delta _{s \rightarrow t}, \end{aligned}$$

where $\delta _{s \rightarrow t} = 1$ if the greedy routing from the source *s* to the target *t* is successful, and otherwise $\delta _{s \rightarrow t} = 0$.

**Figure 1:**
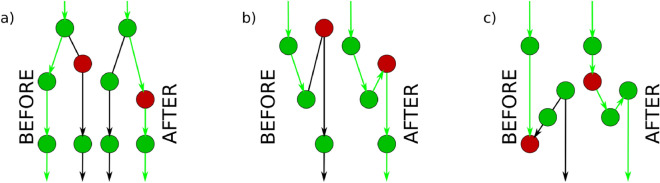
Possibly elementary changes in greedy routes when displacing a single node. In all panels, the target of the path (highlighted in green) is positioned at an arbitrarily large distance downward, and the displaced node is highlighted in red. Panel (**a**) shows the case of re-routing, whereas panels (**b**,**c**) display clogging and unclogging.

Naturally, the optimisation algorithm must displace at least a part of the nodes to induce changes in the routing of the paths. Before describing the details of the optimisation, it is instructive to first consider the possible effects coming from the displacement of a single node from the point of view of successful and unsuccessful paths, as listed in Fig. 1. The simplest scenario, where the displacement of a given node has no effect on the considered greedy path is not shown in the figure. In case the displaced node is not part of the greedy path before the change but is adjacent to one of the nodes in the path, the displacement may reroute the path by directing it through the displaced node, as shown in Fig. 1a. If the displaced node is adjacent to the last node of an unsuccessful greedy path, the displacement might eliminate the clogging, as indicated in Fig. 1b. The clogging in a greedy path can be relieved also by displacing the before the last node, as shown in Fig. 1c. It is important to note that for all the different changes in the routing that are listed in Fig. 1 the “inverse” may also happen at the displacement of nodes (e.g., instead of eliminating the clogging in an unsuccessful path we may accidentally induce the clogging of a previously successful path).

**Figure 2:**
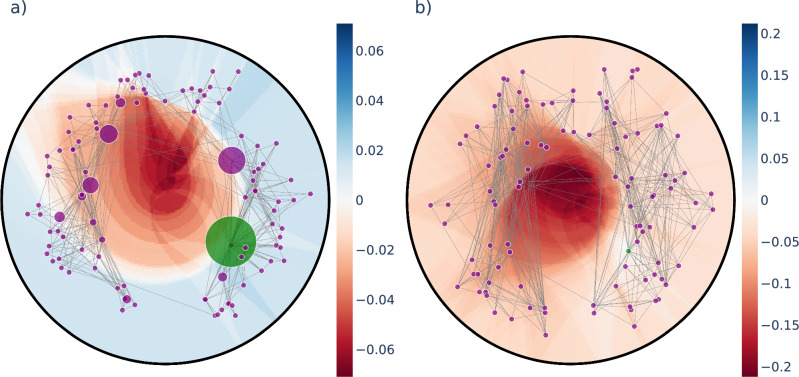
Change in the success ratio under relocation of a single node. The node to be relocated is shown in green and the background colour indicates the change in $p_{\textrm{s}}(\{r_i,\theta _i\})$ defined in Eq. (2) if the node is moved to the given position. The node size is proportional to the number of unsuccessful paths ending at the given node. In panel (**a**) we show the results for a PSO network of $N=1024$ nodes at the beginning of the optimisation process, whereas panel (**b**) depicts the heat-map at the end of the optimisation.

To illustrate that the displacement of even a single node can change the greedy routing in a non-trivial way, in Fig. 2. we show heat maps of the change in the success ratio when relocating a randomly chosen node within the disk defined by the outermost node in a PSO network. According to Fig. 2a, the regions where the change is positive (indicated by the different shades of blue) form intricate patterns together with the regions where the change becomes negative (shown by the different shades of red). However, under a greedy routing optimisation process we expect that the target regions for relocation with a positive change in $p_{\textrm{s}}(\{r_i,\theta _i\})$ will shrink. This is consistent with Fig. 2b, showing the achievable change in the success ratio for relocating the same node in the same network as in Fig. 2a at the end of our optimisation algorithm, where basically no positive change can be obtained by relocating the chosen node. In Sect. S1 in the Supplementary Information we study the shrinkage of the target area with positive change in $p_{\textrm{s}}$ over the iterations in more details.

### Greedy routing optimisation algorithm

Our approach to optimising the greedy routing paths is inspired by the concept of simulated annealing ^34^ in statistical physics. In this general framework, the optimisation task is transformed into the problem of finding the energy minimum in a complicated energy landscape over the parameter space, and a local optimum is found with the help of a Markov-chain Monte-Carlo method. During this optimisation, random moves are considered in the parameter space, and the acceptance probability of a given move depends on the energy difference between the two settings and also on a parameter *T* analogous to the temperature. By starting the annealing procedure at high temperatures, almost all moves are accepted, allowing the exploration of the parameter space, whereas the gradual “cooling” of the system by lowering the *T* suppresses the acceptance of moves that increase the energy and eventually drives the algorithm into a local energy minimum.

In our case, the “energy” for a given set of node coordinates $\{r_i,\theta _i\}$ is defined as

3 $$\begin{aligned} E(\{r_i,\theta _i\}) =1-p_{\textrm{s}}(\{r_i,\theta _i\}), \end{aligned}$$

where $p_{\textrm{s}}(\{r_i,\theta _i\})$ is calculated according to (2) and the energy difference for a transition from $\{r_i,\theta _i\}$ to $\{r'_i,\theta _i'\}$ is simply $\Delta E=E(\{r'_i,\theta _i'\})-E(\{r_i,\theta _i\})=p_{\textrm{s}}(\{r_i,\theta _i\})-p_{\textrm{s}}(\{r'_i,\theta '_i\})$. When sampling new coordinate settings, the acceptance probability of an actual transition from the current state to the next one follows the Metropolis-Hastings rule^35,36^ as

4 $$\begin{aligned} P\left( \{r_i,\theta _i\}\rightarrow \{r_i',\theta _i'\}\right) =\left\{ \begin{array}{ll} 1 &{} \text{ if } \Delta E < 0,\\ {\mathrm e}^{-\frac{\Delta E}{T}}={\mathrm e}^{\frac{p_{\textrm{s}}(\{r'_i,\theta '_i\})-p_{\textrm{s}}(\{r_i,\theta _i\})}{T}} &{} \text{ otherwise. } \end{array} \right. \end{aligned}$$

The natural question arising at this point is how to sample from the possible arrangements of the nodes in the native disk. Given any current set of the node positions $\{r_i,\theta _i\}$, the simple choice we take for gaining a new sample $\{r'_i,\theta '_i\}$ is to displace a single node (as already illustrated in Fig. 2), where the new position for the chosen node is drawn from a uni-modal distribution centred on the current position of the node (where the details of the distribution are given in the Methods). The pseudo-code for optimising the success ratio in this approach for a network *G*(*V*, *E*) with a node set *V*, an edge set *E* and some specified initial node coordinates is given in Algorithm 1.

**Algorithm 1:**
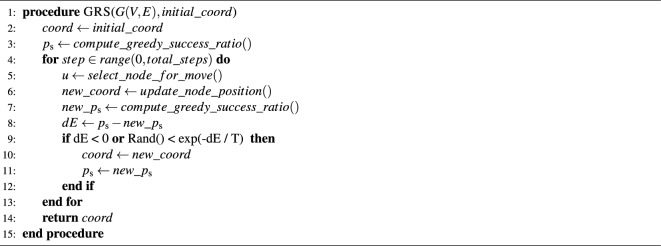
Greedy routing annealing

The overall framework for greedy routing optimisation as defined above allows several possibilities for choosing the node to be moved, e.g., we can choose uniformly at random from all the nodes, or choose according to a probability depending on some structural property (e.g., the degree), or choose according to a probability depending on the number of clogged greedy routes containing the given node, etc. The most straightforward approach is the uniform random choice, which does not require any additional information. In the following, we shall refer to the sampling procedure according to this choice as “random sampling”. In our studies, we also tested annealing procedures where the probability to be chosen was proportional to the node degree (the corresponding sampling method shall be referred to as “degree dependent sampling”), or to the number of clogged greedy paths starting from the given node (“clogged source sampling”) or to the number of clogged greedy paths targeting the given node (“clogged target sampling”).

During the annealing simulations in the present work we used a cooling scheme where the temperature was decreased as $T=T_{0}/(1+n_{\textrm{e}})$, where $T_{0}$ denotes the starting temperature and $n_{\textrm{e}}$ is equal to the number of passed epochs, with one epoch corresponding to a number of iterations equal to the number of nodes. This choice corresponds to one of the well-known cooling schemes in simulated annealing (and the code we provide allows replacing this with a user defined cooling scheme as well). The simulations were stopped uniformly after 400 epochs, allowing a fair comparison between the different networks according to the average number of displacements per node.

However, since the optimisation problem we are dealing with is analogous to the location of the ground state in a complicated energy landscape, the number of local minima can increase super linearly with the system size. Therefore, in a larger system the amount of exploration steps needed for finding a suitably good solution can grow faster than linear. Based on that, a natural alternative for the stopping criterion (made possible by the code we provide) can be formulated based on the relative improvement in $p_{\textrm{s}}$ between subsequent epochs (or over a certain range of the last epochs). Under this setting, the annealing stops if the relative improvement in $p_{\textrm{s}}$ drops below a certain threshold. Nevertheless, we still used a fixed number of epochs in the simulations as mentioned above, in order to make the preparation of the statistics describing the behaviour of various quality scores as a function of the number of epochs straight forward.

### Greedy routing optimisation of networks

**Figure 3:**
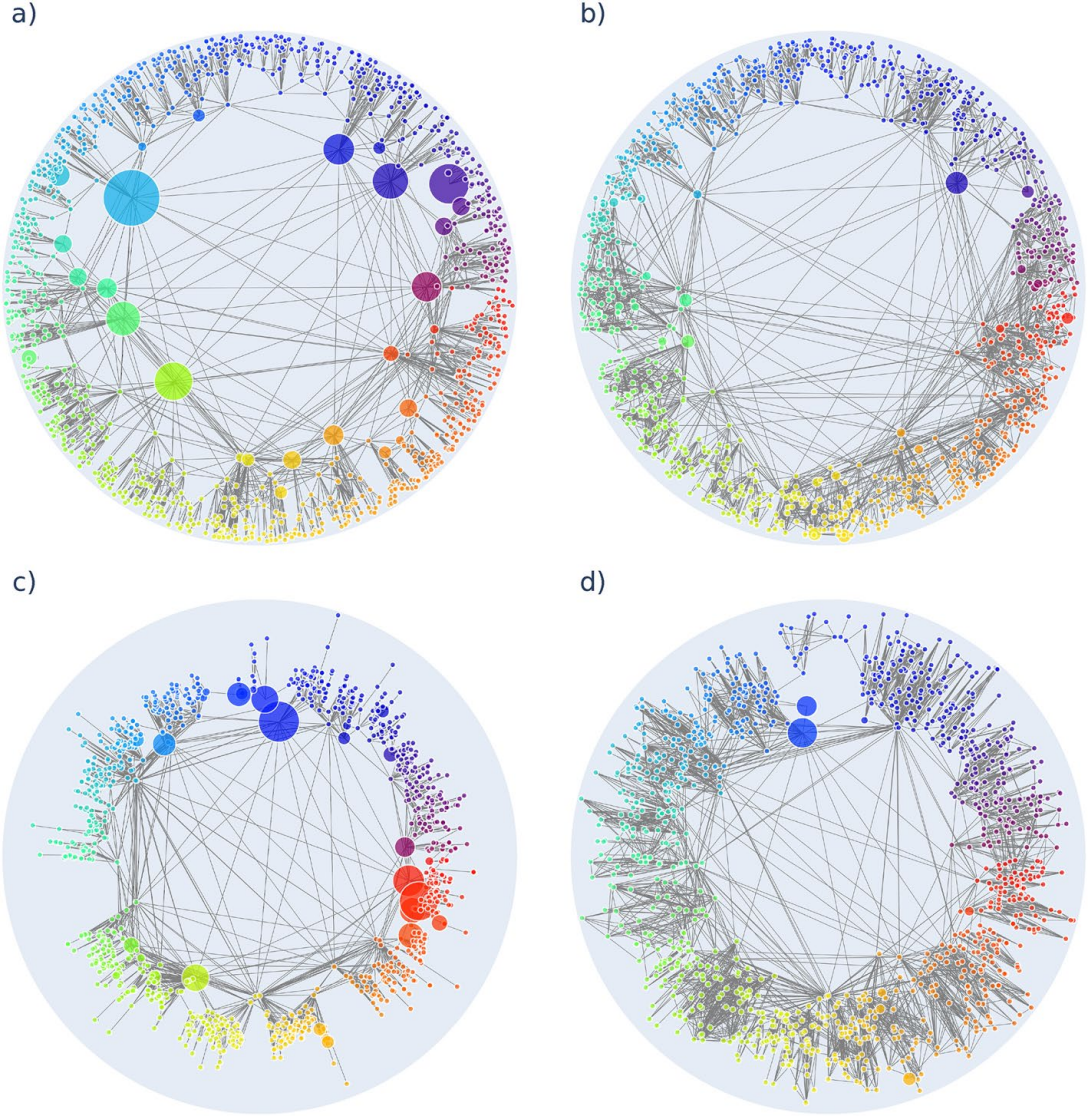
Improving the greedy routing in a PSO network. The layout of the network at the end of the network generation process according to the PSO model ($N=1024$, $m=4$, $\beta =0.5$, $T=0.1$) is shown in panel (**a**), whereas the modified layout according to the optimisation algorithm is displayed in panel (**b**). In both panels the node size is proportional to the number of greedy routes prematurely ending at a given node, whereas the node colors indicate the initial angular coordinates. In panel (**c**) we show the embedding of the same network according to Mercator^8^, where the colouring of the nodes is adapted to this new starting layout. Panel (**d**) depicts the result obtained by optimising the layout shown in panel (**c**).

Although the greedy routing optimisation procedure we described can be viewed as a hyperbolic embedding algorithm (when starting from random node positions), it is much more convenient to use it as an auxiliary method for improving embeddings obtained by other hyperbolic embedding algorithms. To illustrate the kind of results that can be expected from our annealing framework, in Fig. 3.  we show layouts of a network of $N=1024$ nodes generated by the PSO model both before and after the optimisation. The parameters of the PSO model were set to $m=4$ (controlling the average degree as $\langle k\rangle =2m$), $\beta =0.5$ (governing a gradual outward shift of the nodes during the network generation, leading to a tunable degree decay exponent $\gamma =1+\frac{1}{\beta }$), and $T=0.1$ (corresponding to a temperature-like parameter, controlling the clustering coefficient). In Fig. 3a we show the original layout of the network generated by the PSO model, where the node size is proportional to the number of unsuccessful greedy paths ending at the given node and the colouring of the nodes indicates simply their angular coordinates. In comparison, Fig. 3b displays the network after applying our optimisation framework. Apparently, the node sizes are substantially smaller compared to Fig. 3a, thus, our optimisation is indeed doing its job by modifying the node arrangement in such a way that the number of unsuccessful greedy paths is reduced. The colouring of the nodes in Fig. 3b is indicating their original angular coordinate as given in Fig. 3a and according to that, the overall layout of the network remained quite similar to the original one. In panels Fig. 3c,d we show a similar comparison between the same PSO network embedded first by the Mercator algorithm^8^ (Fig. 3c), and then optimised according to the simulated annealing procedure proposed in the present work (Fig. 3d). Here and throughout the rest of the paper, we used Mercator with default settings, omitting optional refinement steps in the embedding. Again, the node sizes are seemingly smaller in Fig. 3d compared to Fig. 3c, thus, our optimisation has improved the greedy navigability of the embedding. Meanwhile, based on the colouring of the nodes (indicating this time the angular coordinate in the Mercator embedding) the organisation of the network on a global scale has remained mostly intact.

**Figure 4:**
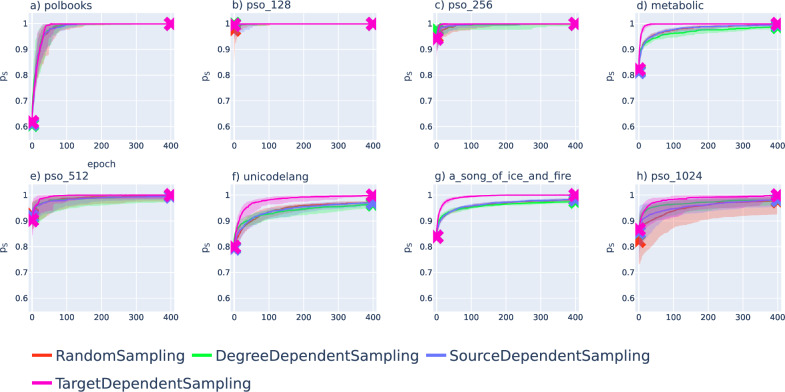
Improvement of the success ratio during the optimisation for embeddings obtained with Mercator^8^. We plot $p_{\textrm{s}}(\{r_i,\theta _i\})$ defined in (2) as a function of the number of epochs for random sampling (red), degree dependent sampling (green), clogged source dependent sampling (blue) and clogged target dependent sampling (purple). The curves correspond to the median over 20 samples and the shaded region around the curves falls between the 40$^{\textrm{th}}$ and 60$^{\textrm{th}}$ percentiles. For better visibility, we marked both the starting values (before the optimisation) and the ending values (after 400 epochs) with ’x’ symbols. The results are shown for the network of political books ($N=105$, $L=441$) in panel (**a**), for a PSO network with $N=128$ nodes and $L=618$ links in panel (**b**), a PSO network with $N=256$ nodes and $L=1178$ links in panel (**c**), the metabolic network ($N=453$, $L=2025$) in panel (**d**), a PSO network with $N=512$ nodes and $L=2264$ links in panel (**e**), the unicodelang network ($N=858$, $L=1245$) in panel (**f**), the network between fictional characters ($N=796$, $L=2823$) in panel (**g**) and a PSO network with $N=1024$ nodes and $L=4402$ links in panel (**h**).

We tested the performance of our optimisation approach on several synthetic and real networks, including PSO networks of size $N=128$, $N=256$, $N=512$ and $N=1024$, a co-purchasing network of political books^37^ with $N=105$ nodes and $L=441$ links, a metabolic network^38^ of $N=453$ nodes and $L=2025$ links, a bipartite language network^39^ containing countries and spoken languages $N=858$ nodes (10 disconnected nodes were discarded) and $L=1245$ links and the fictional social network^40^ of the characters appearing in the fantasy series “A Song of Ice and Fire” with $N=796$ nodes and $L=2823$ links. The real networks were obtained from^41^. In Fig. 4 we show the results by plotting the success ratio calculated according to (2) as a function of the number of epochs when the optimisation was started on embeddings obtained with Mercator^8^.

**Figure 5:**
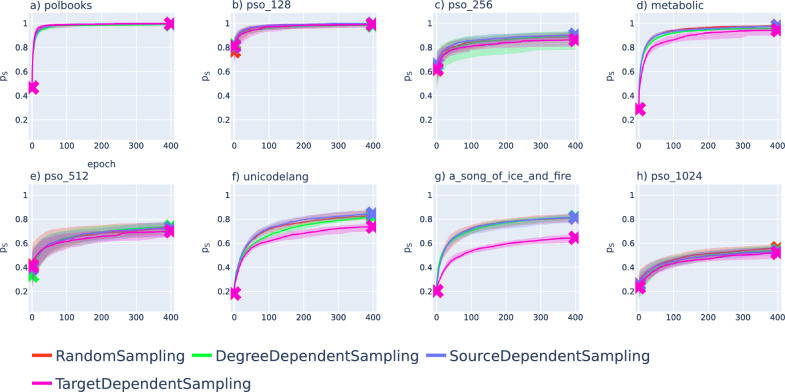
Improvement of the success ratio during the optimisation for embeddings obtained with hyperbolic ISOMAP^24^. The $p_{\textrm{s}}(\{r_i,\theta _i\})$ defined in (2) is plotted as a function of the number of epochs for random sampling (red), degree dependent sampling (green), clogged source dependent sampling (blue) and clogged target dependent sampling (purple). The curves indicate the median over 20 samples (where the starting and ending values are marked by ’x’ symbols) and the shaded region around the curves is spanning between the 40$^{\textrm{th}}$ and 60$^{\textrm{th}}$ percentiles. The results are shown for the network of political books ($N=105$, $L=441$) in panel (**a**), for a PSO network with $N=128$ nodes and $L=618$ links in panel (**b**), a PSO network with $N=256$ nodes and $L=1178$ links in panel (**c**), the metabolic network ($N=453$, $L=2025$) in panel (**d**), a PSO network with $N=512$ nodes and $L=2264$ links in panel (**e**), the unicodelang network ($N=858$, $L=1245$) in panel (**f**), the network between fictional characters ($N=796$, $L=2823$) in panel (**g**) and a PSO network with $N=1024$ nodes and $L=4402$ links in panel (**h**).

According to Fig. 4., the fraction of successful greedy paths is increasing in all networks during the optimisation, and in some cases, we can even achieve $p_{\textrm{s}}(\{r_i,\theta _i\})=1$, meaning that all greedy paths become successful due to our optimisation. Naturally, both the $p_{\textrm{s}}(\{r_i,\theta _i\})$ at the end of the optimisation and the relative performance of the different annealing schemes vary over the studied networks. In Fig. 5. we show the improvement in $p_{\textrm{s}}$ observed when the optimisation was started from embeddings of the same networks obtained with hyperbolic ISOMAP^24^. Similarly to Fig. 4., the success ratio is increasing for all networks under the optimisation. However, since $p_{\textrm{s}}$ in the initial embedding is usually lower compared to the same score in the initial Mercator embeddings in Fig. 4., the value at the end of the optimisation (terminated after 400 epochs similarly to the case of Mercator embeddings) can also fall behind the results seen in Fig. 4. Nevertheless, the relatively low initial success ratio allows for greater relative improvement by the optimisation, as we can observe e.g., in the case of the metabolic network (Fig. 5d), the unicodelang network (Fig. 5f) and the network of fictional characters (Fig. 5g).

By comparing the results for different systems, it seems that the annealing scheme using target dependent sampling outperformed the other annealing schemes when starting the optimisation from Mercator embeddings in the case of Fig. 4d,f–g (the metabolic network, the unicodelang network and the network between fictional characters). In contrast, according to Fig. 5d,f–g, when embedding the same networks with hyperbolic ISOMAP, the target dependent sampling seems to fall behind the other alternative annealing schemes according to the speed of increase in the success ratio.

**Figure 6:**
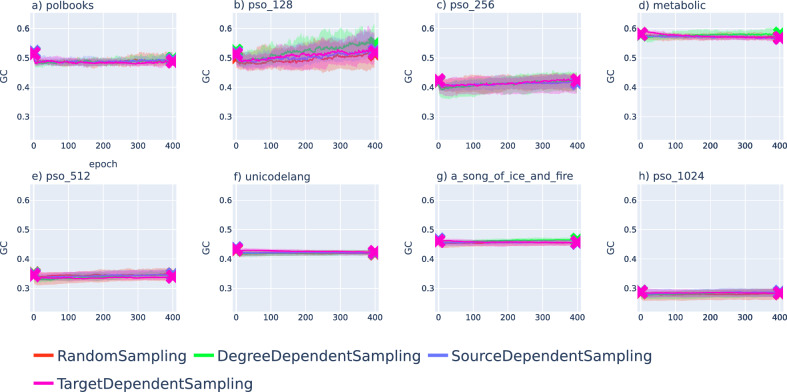
Change in the geometrical congruence under the optimisation of embeddings obtained with Mercator^8^. We show the GC defined in (5) as a function of the number of epochs for the same networks (initially embedded with Mercator^8^) as in Fig. 4, where the colour of the curves encode the annealing scheme, the value of the curve shows the median over 20 samples (where the starting and ending values are marked by ’x’ symbols) and the shaded region around the curves is spanning between the 40$^{\textrm{th}}$ and 60$^{\textrm{th}}$ percentiles. The results are shown for the network of political books in panel (**a**), for a PSO network with $N=128$ nodes in panel (**b**), a PSO network with $N=256$ in panel (**c**), the metabolic network in panel (**d**), a PSO network with $N=512$ nodes in panel (**e**), the unicodelang network in panel (**f**), the network between fictional characters in panel (**g**) and a PSO network with $N=1024$ nodes in panel (**h**).

**Figure 7:**
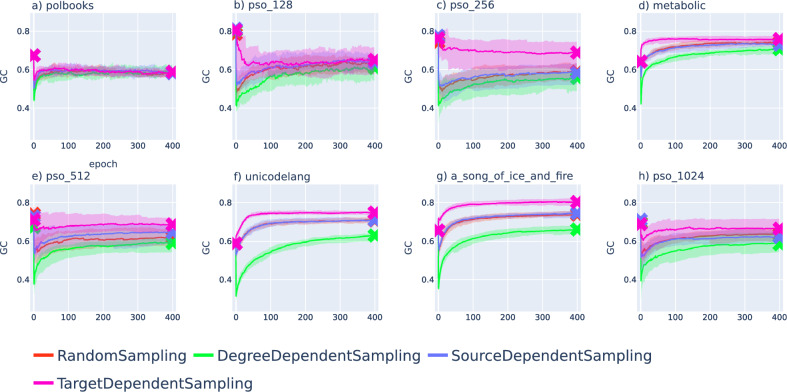
Change in the geometrical congruence under the optimisation of embeddings obtained with hyperbolic ISOMAP^24^. The GC is plotted as a function of the number of epochs for the same networks (initially embedded with hyperbolic ISOMAP^24^) as in Fig.5, where the colour of the curves encode the annealing scheme, the value of the curve indicates the median over 20 samples (where the starting and ending values are marked by ’x’ symbols) and the shaded region around the curves is falling between the 40$^{\textrm{th}}$ and 60$^{\textrm{th}}$ percentiles. The results are shown for the network of political books in panel (**a**), for a PSO network with $N=128$ nodes in panel (**b**), a PSO network with $N=256$ in panel (**c**), the metabolic network in panel (**d**), a PSO network with $N=512$ nodes in panel (**e**), the unicodelang network in panel (**f**), the network between fictional characters in panel (**g**) and a PSO network with $N=1024$ nodes in panel (**h**).

Since our optimisation scheme is specifically tailored for optimising the success ratio, it is natural to ask what happens to other alternative quality measures during the simulated annealing. In Figs. 6, 7 we show the geometrical congruence^33^, measuring the similarity between the geodesic distances and the projected topological distances governed by the networks structure, where the precise definition of this score is given by Eq. (5) in the Methods.

The results show a mixed picture, as in some cases the GC-score is slightly smaller after the optimisation, whereas in other cases the annealing terminates with a higher GC-score value compared to the initial embedding. Furthermore, the curves can show a non-monotonous behaviour, having either a minimum or a maximum in the studied interval. Nevertheless, we would like to point out that the overall change in the GC-score (when comparing the starting value and the value at the end of the optimisation) is minor in most of the cases.

We provide more results on the proposed optimisation framework in the Supplementary Information. In Sect. S2 we examine the behaviour of both the success ratio and the GC-score when the simulated annealing is started from a random embedding for the same networks that are studied here. According to the results, full navigability (where the success ratio is reaching $p_{\textrm{s}}=1$) can be achieved in this case as well for some of the studied networks. However, in most cases the success ratios at the end of the optimisation are slightly below the values we observed when starting the annealing from embeddings obtained with Mercator^8^ and are roughly at the same level we can observe in experiments started from embeddings according to the hyperbolic ISOMAP^24^ method. In parallel, according to Fig. S3., major improvement can be observed in the geometrical congruence as well for most of the networks.

In Sect. S3 in the Supplementary Information we study the effect of our annealing framework on further quality scores, such as the mapping accuracy^42^, the area under the receiver operating characteristic curve and the area under the precision-recall curve in graph reconstruction^43,44^, the greedy routing score^24^ as defined in Eq.(1) and the greedy routing efficiency^33^. According to the results, the mapping accuracy (Sect.S3.1) and the quality scores related to graph reconstruction (Sect.S3.2) decrease when starting the optimisation from embeddings obtained with Mercator^8^. This can be viewed as a sort of “cost” we have to pay for achieving the improvement in the success ratio shown in Fig. 4. In case of annealing experiments starting from hyperbolic ISOMAP^24^ embeddings the picture is more mixed: the above quality scores (that are not related to greedy routing) either stagnate or slightly decrease for most networks, but in some examples we can also observe a small increase instead.

The scores related to greedy routing (studied in Sect.S3.3) either remained close to their original value, occasionally with a slight decay, or improved when starting the simulated annealing from an embedding obtained with Mercator^8^. In contrast, these scores showed an increasing nature under the annealing experiments started from embeddings generated by hyperbolic ISOMAP^24^ for all studied networks.

The behaviour of the same scores was also studied in annealing experiments started from random embeddings, where we observed (in most cases major) improvement in all score values over the iterations. Hence, the embeddings our annealing framework finds are advantageous from the point of view of a wide range of quality measures compared to random node coordinates.

When combining the figures related to the different quality scores from both the main paper and Supplementary Information it is apparent that the different sampling methods have lead to different performance in a number of examples. A plausible explanation for this is that our annealing framework based on single node relocation is likely to explore only a limited fraction of the possible configuration space, and in certain cases the different sampling methods drive the system to distinct “valleys” (local minima) in this abstract space. Since the degree-based sampling is one of the sampling methods that surpassed the others in several examples, in Sect. S4 in the Supplementary Information we examined whether the displacement of hubs play an important role in cases where it seems to be more successful compared to the other sampling methods. Related to that we carried out experiments where a part of the nodes (selected based on degree) were fixed during the optimisation. According to the results, hub position has a strong effect on embedding quality when starting the optimisation from random initial node positions, but not when starting from embeddings generated by Mercator. The likely reason behind this is that Mercator places hubs to positions that are already very close to optimal, and for such initial configuration, it is sufficient to displace the lower degree nodes only.

In order to provide an overview of the gains and losses we can observe in the different quality scores, in Sect. S5 of the Supplementary Information we display violin plots showing the distribution of the score values observed at the end of the optimisation processes. Here we include all four sampling methods as well as the results for experiments with fixed hubs during the annealing.

A further question studied in the Supplementary Information is to what extend do the statistical properties of graphs re-generated from the embeddings match with those of the original networks. In Sect. S6 in the Supplementary Information we analyse graphs obtained by drawing between node pairs according to probabilities dictated by the $\mathbb {S}^1/\mathbb {H}^2$ model^8^. According to the results, the degree distribution and the average clustering coefficient of these reconstructed graphs match with that of the original networks quite well for Mercator^8^ embeddings, and compared to that with a slightly decreased accuracy for the optimised layouts. This indicates that the annealing process can also affect these fundamental statistics of the re-constructed graphs to some extent, and thereby, the decreased similarity compared to Mercator^8^ embeddings is yet a further cost we have to pay for the possible gain in greedy routing.

## Discussion

The navigability of networks in the hyperbolic space is a topic of fundamental interest ^4,10,22,31,33^. Already at the introduction of the first hyperbolic network models, one of the noted advantages of the graphs generated by these approaches was that the geodesic paths seemed to be aligned with the topological shortest paths, enabling an efficient greedy routing protocol for navigation in the network ^4,10^. Nevertheless, in a recent work some concerns were raised regarding the congruence between hyperbolic networks and their underlying geometry ^33^, where it was shown that in some cases, the greedy routing may lead to unsuccessful paths between a considerable fraction of the node pairs.

Related to the above topic, in the present paper we introduced a simulated annealing framework for improving the greedy navigability of networks embedded in the hyperbolic space. According to the results, our approach is able to increase the success ratio for both synthetic graphs generated by hyperbolic network models and real networks embedded into the hyperbolic space by some graph embedding technique, reaching in some examples the maximum score, corresponding to layouts where all greedy paths are successful in reaching their targets. Although in theory, the algorithm can be used as an embedding method on its own, in practice it is more useful to use it as an auxiliary procedure that can improve the output of other hyperbolic embedding methods due to the well-known high computation cost of simulated annealing methods.

Besides the success ratio, we have also monitored the change of several other quality measures, including the geometrical congruence^33^, the mapping accuracy^42^, the area under the receiver operating characteristic curve and the area under the precision-recall curve in graph reconstruction^43,44^, the greedy routing score^24^ and the greedy routing efficiency^33^. As expected, the scores related to greedy routing usually change in the positive direction under the optimisation even when starting the simulated annealing from a high quality embedding such as the output by Mercator^8^. In the meantime, the quality measures that are more independent from the success ratio such as the mapping accuracy or the scores measuring the performance in graph reconstruction can show a slightly decreasing tendency if the score of the initial embedding is high. The geometrical congruence showed a mixed behaviour, sometimes increasing, in other cases decreasing over the iterations. Nevertheless, the losses observed in the various measures during the experiments starting from embeddings obtained with Mercator were usually minor.

The behaviour of the above quality scores shows a somewhat different picture when starting the optimisation from embeddings obtained with the hyperbolic ISOMAP^24^ method. Under this circumstance the quality indicators related to greedy routing where always improved by the annealing procedure, and in some cases a part of the scores not related to greedy paths were also increasing. In parallel, all studied quality scores showed an increasing tendency when starting the optimisation from a uniformly random node coordinates, usually achieving a significant improvement at the end of the process.

In conclusion, the simulated annealing framework we propose is a general approach for improving the navigability of hyperbolic networks. Although here we focused on increasing the fraction of successful paths, with minor changes made to the energy function, the method could also be used for optimising with respect to other quality scores instead. Our results showed that this approach was capable of maximising the success ratio in some examples, hence, similar optimisation algorithms aimed at improving the layout of hyperbolic networks with respect to alternative other measures have a great potential to be effective as well.

## Methods

### Calculating the success ratio

Since the calculation of the success ratio, $p_{\textrm{s}}$, has to be carried out a large number of times, it is necessary to design an efficient algorithm for doing so. Although various algorithms can be defined that differ in their details, the time complexity is roughly $\mathcal {O}(N^2 \log N + N^2 \langle k\rangle )$ in all cases, where *N* is the number of nodes and $\langle k\rangle$ denotes the average degree of the network. Our solution builds on two main parts. First, for each (directed) source and target pair we identify the neighbour that is next in the greedy routing path in $\mathcal {O}(N^2 \langle k\rangle )$ time. Second, we implement the propagation along the greedy paths in $\mathcal {O}(N^2 \log N)$ by iteratively applying a path halving algorithm $log_2 N$ times. This many iterations are enough for the worst case scenario, that is when a successful greedy routing path exists that visits each node. The path halving sweeps can be calculated very fast, especially when vectorised arithmetics are available. The pseudo-code of this procedure is given in Algorithm 2.

**Algorithm 2:**
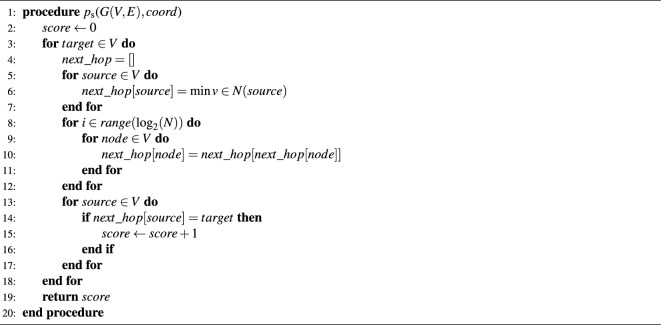
Greedy success ratio

### Sampling the target position for displacement

**Figure 8:**
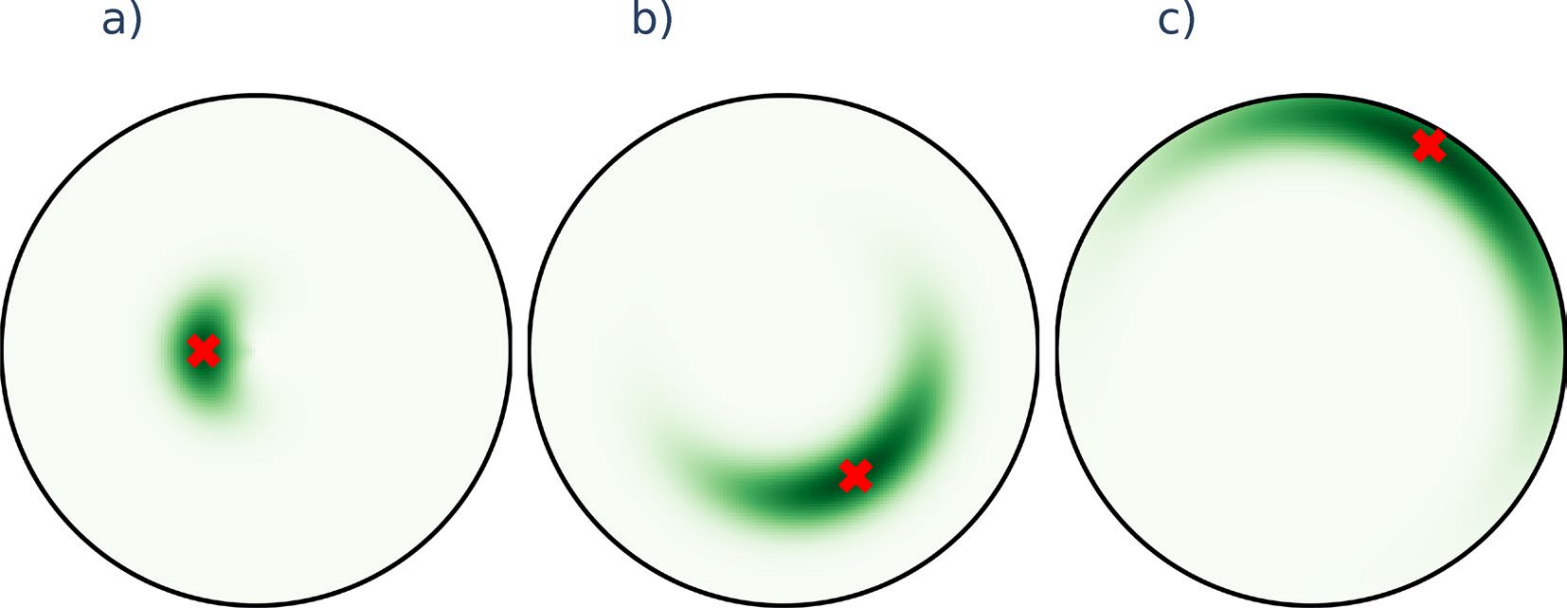
Distribution of the possible new positions for a node. The green heat map indicates the probability density and the current position of the chosen node is shown by the red marker. A network of size $N=128$ was used for generating the panels, where the chosen node is close to the disk center in panel (**a**), at medium range from the disk center in panel (**b**) and close to the disk periphery (far from the center) in panel (**c**).

In every iteration, after selecting the node we try to displace, we also need to specify the possible new position for the selected node. This position was obtained by independently sampling a new angular coordinate from a normal distribution centered on the angular coordinate of the chosen node and a new radial coordinate from a truncated normal distribution centered on the radial coordinate of the chosen node, where the distribution was restricted to the [0, *R*] interval with *R* denoting the disk radius of the network. Illustrations of the resulting distribution are provided in Fig. 8.

Although a sampling distribution where the level lines of the density form hyperbolic circles around the chosen node might seem as a more natural choice, according to our experience the sampling distribution proposed here yields a faster increase in the success ratio over the iterations. The likely reason for this is that when the chosen node has a relatively large radial coordinate (such as e.g., in the case of Fig. 8c) our sampling distribution allows the exploration of a much larger angular region, which seems to help finding more suitable new positions.

### The geometric congruence

The geometric congruence was introduced in Ref^33^ as a general measure for quantifying the alignment between the network topology and an underlying geometry. By assuming that we can evaluate the distance between any node pair, the geometric congruence is formulated as

5 $$\begin{aligned} {\mathrm GC}(\{r_i, \theta _i\}) = \frac{2}{N (N-1) - L} \sum _{i =1}^{N} \sum _{\begin{array}{c} j=1 \\ j \notin N(i) \end{array}}^{i-1} \frac{DIST(i, j)}{PTSP(i, j)}, \end{aligned}$$

where *DIST*(*i*, *j*) is the distance between node *i* and *j* according to the geometry, which in our case becomes the distance along the geodesic of the hyperbolic disk; and *PTSP*(*i*, *j*) is the projected topological shortest path, that is the sum of distances along a topological shortest path (or the average of these sums if multiple topological shortest paths exist) starting on *i* and ending on *j*. Note that the summing runs over only the nonadjacent node pairs, since *j* cannot be a member in the neighbour set of node *i* (denoted by *N*(*i*) in the formula).

### Supplementary Information

Supplementary Information.

## Data Availability

All data generated during the current study are available from the corresponding author upon request. The “polbooks” network was compiled and hosted by V. Krebs. Currently, it is available from several network dataset sources such as the KONECT project at http://www.orgnet.com. The metabolic network is available from Ref.^38^. The “unicodelang” network was compiled from the data available at the Territory-Language Information page of the unicode.org project and accessed via the KONECT project at https://www.unicode.org/cldr/cldr-aux/charts/25/supplemental/territory_language_information.html. The “a song of ice and fire” network was compiled and hosted by A. Beveridge at https://github.com/mathbeveridge/asoiaf.

## Code availability

Our code is available at the following link: https://github.com/bsulyok/gra.
