# Supplementary material for: Greedy routing optimisation in hyperbolic networks
Source: Sci Rep. 2023 Dec 27;13:23026. doi: 10.1038/s41598-023-50244-8 (PMC10754836; doi:10.1038/s41598-023-50244-8)
Supplement: Supplementary file 1 — Supplementary Information. [file 41598_2023_50244_MOESM1_ESM.pdf]

Greedy routing optimisation in hyperbolic networks – Supplementary Information

Bendegúz Sulyok[1] **and Gergely Palla**[1,2]

[1]Dept. of Biological Physics, Eötvös Loránd University, H-1117 Budapest, Pázmány P. stny. 1/A, Hungary
[2]Data-Driven Health Division of National Laboratory for Health Security, Health Services Management Training Centre, Semmelweis University, H-1125, Kútvölgyi út 2, Budapest, Hungary
*gergely.palla@emk.semmelweis.hu

S1 Possible change in the p_s under single node reallocation

We have shown in Fig. 2. in the main paper that before the start of the optimisation, the area of the target region where the relocation would result in a positive change in p_s is significantly larger compared to the area of a similar region at the end of the optimisation for a particular node in the studied network. Here we show that this is true for the majority of the nodes by extending the analysis to all of the nodes in the same network. Related to that, in Fig.S1. we show the average relative size of the target region where the relocation would increase p_s as a function of the number of epochs. The obtained curves show a decreasing tendency both when starting the optimisation from random coordinates (Fig.S1a), from an embedding obtained with hyperbolic ISOMAP[1] (Fig.S1b) or from an embedding obtained with Mercator[2] (Fig.S1c).

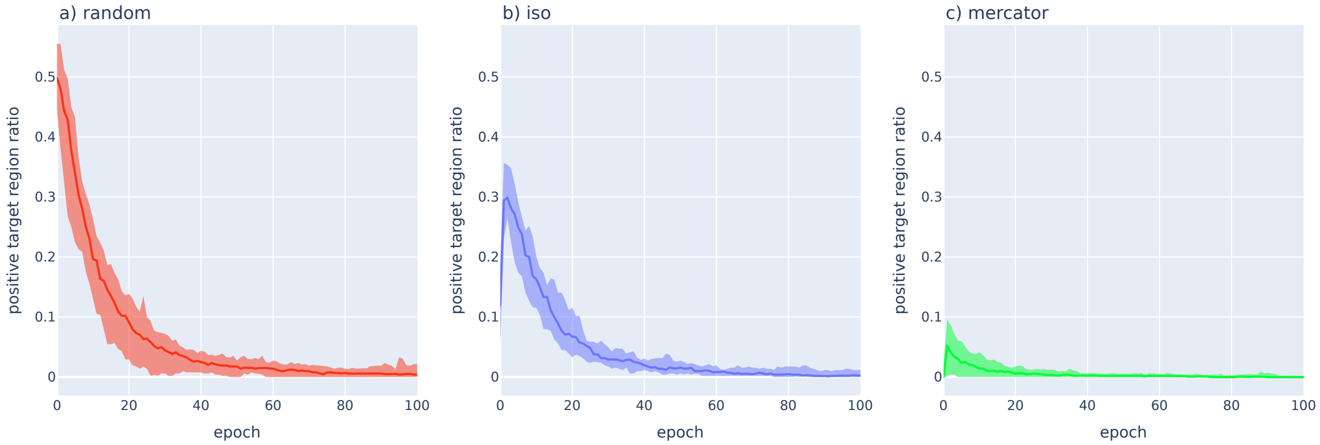

Figure S1. Relative size of the target region for relocation with a positive change in p_s. After each epoch during the annealing we considered all possible single-node relocation to target positions distributed uniformly, and examined whether the given relocation would increase or decrease the p_s. The fraction of target positions with a positive change in p_s is shown as a function of the number of epochs for annealing procedures starting from either random initial node coordinates (panel a), or an embedding obtained with hyperbolic Isomap[1] (panel b), or an embedding obtained with Mercator[2] (panel c). The curves indicate the median value, and the shaded region around the curves is spanning between the 45% and the 55% percentile.

S2 Optimisation starting from random coordinates

We have also run experiments where the initial embedding of the networks was obtained by distributing the nodes uniformly at random in a native disk with radius equal to the radius of a PSO network with the same size. When applied in this manner, our method can be viewed as a full-fledged hyperbolic embedding algorithm. In Fig. S2. we show the change in the success ratio as a function of the number of epochs during the optimisation for the same networks that are studied in the main paper. According to the results, p_s undergoes a major improvement for all studied networks, reaching even the possible maximal score for the polbooks network (Fig. S2a), the PSO network with 128 nodes (Fig. S2b) and the metabolic network (Fig. S2d). This means that starting from a random initial state, our algorithm was able to achieve state where all greedy paths become successful.

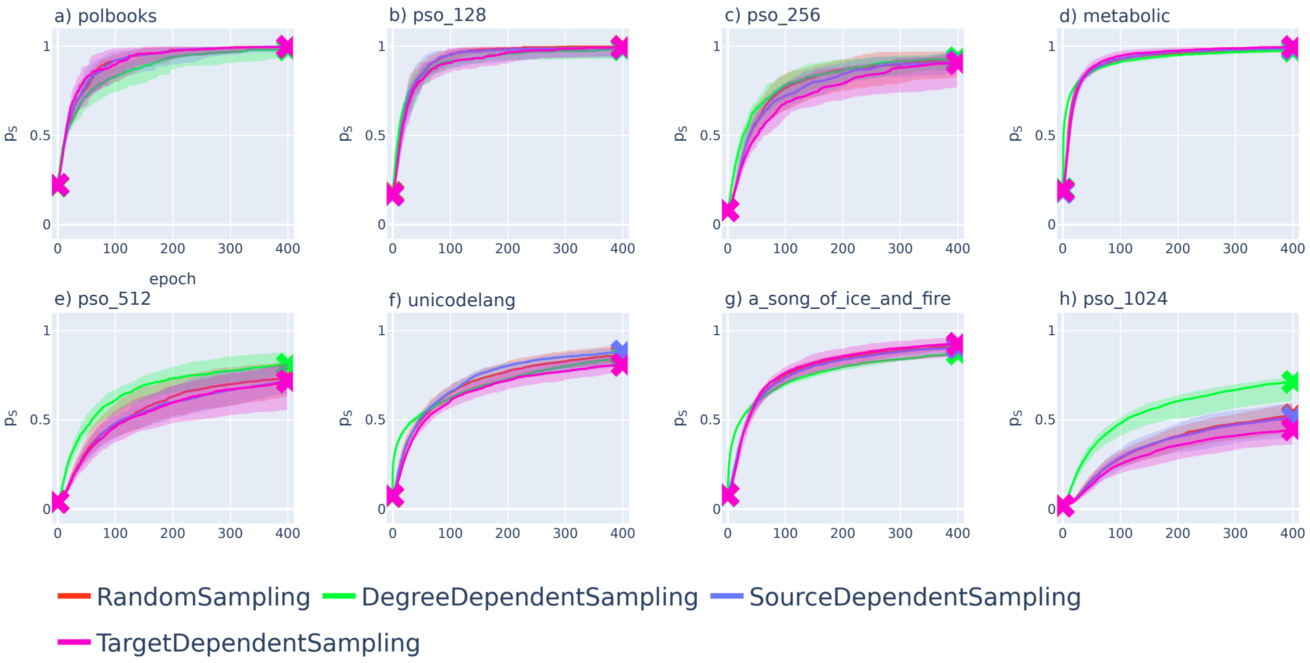

Figure S2. Improvement of the success ratio when starting the optimisation from a random initial embedding. We plot $p_s(\{r_i, \theta_i\})$ defined in Eq.(2) in the main paper where the curve indicates the median over 20 instances as a function of the number of epochs (where the starting and ending values are marked by 'x' symbols). The colour of the curves indicates the annealing scheme and the shaded region around the curves falls between the 40th and 60th percentiles. The results are shown for the network of political books in panel (a), for a PSO network with $N = 128$ nodes in panel (b), a PSO network with $N = 256$ nodes in panel (c), the metabolic network in panel (d), a PSO network with $N = 512$ nodes in panel (e), the unicodelang network in panel (f), the network between fictional characters in panel (g) and a PSO network with $N = 1024$ nodes in panel (h).

In Fig. S3. we show the geometrical congruence as a function of the number of epochs during the same optimisation procedures. In contrast with Fig. 5. in the main paper, the GC-score shows an improving tendency for all networks, and the magnitude of the change is considerably larger (e.g., the largest increase was observed for the network between fictional character, where GC changed from GC=0.321 to GC=0.480 over the optimisation). Interestingly, the implementation that uses the degree-based sampling seems to strongly outperform the other alternatives for some of the networks in the study regarding this score.

S3 Behaviour of further quality scores during the GS optimisation

We recorded the change in several other quality scores during our numerical experiments with the proposed greedy routing optimisation procedure. In the following subsections we detail the results in terms of these measures both when starting the annealing procedure from random initial coordinates and when optimising the embedding obtained with Mercator[2].

S3.1 The mapping accuracy

The mapping accuracy is intended to measure the similarity between the geometrical distance according to the node coordinates and the shortest path length based on the network structure and is defined simply as the Spearman's correlation coefficient between these two quantities measured over all node pairs[3].

In Fig. S4. we show the mapping accuracy for the studied networks when starting the optimisation from node coordinates generated by Mercator[2], whereas Figs. S5-S6. display the same results for hyperbolic ISOMAP embedding[1] and random initial coordinates, respectively. Since Mercator is usually producing embeddings with good quality measures and our optimisation is focusing on a different quality score, the mapping accuracy shows a decreasing tendency for all networks in Fig. S4, and the magnitude of the change can be quite significant. Hence, the loss in the mapping accuracy can be viewed as a price we have to pay when optimising the a Mercator based embedding with respect to the success ratio.

The situation is somewhat different in the case of the hyperbolic ISOMAP embedding[1] as shown in Fig.S5. Although the mapping accuracy is lower at the end of the optimisation compared to the starting value for most of the studied networks, in

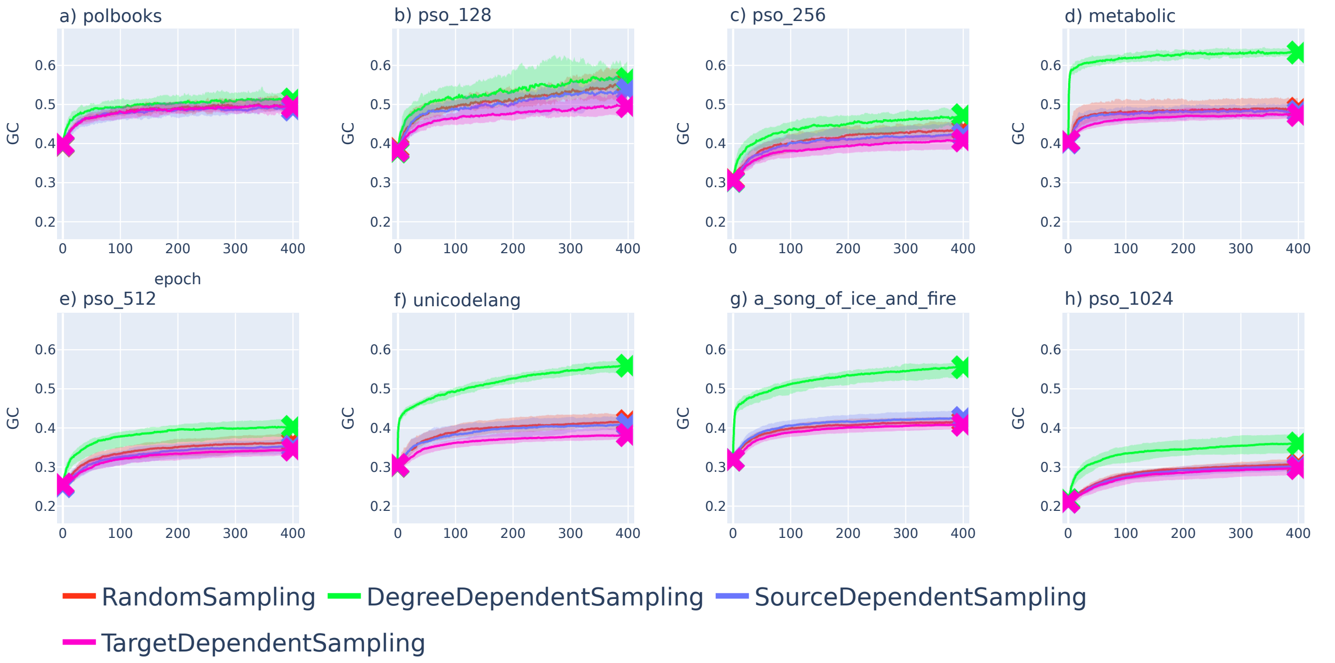

Figure S3. Change in the geometrical congruence when the optimisation is started from a random embedding. We plot median GC$(\{r_i, \theta_i\})$ defined in Eq.(6) in the main paper over 20 samples as a function of the number of epochs (where the starting and ending values are marked by 'x' symbols). The colour of the curves indicates the annealing scheme and the shaded region around the curves falls between the 40th and 60th percentiles. The results are shown for the network of political books in panel (a), for a PSO network with $N = 128$ nodes in panel (b), a PSO network with $N = 256$ nodes in panel (c), the metabolic network in panel (d), a PSO network with $N = 512$ nodes in panel (e), the unicodelang network in panel (f), the network between fictional characters in panel (g) and a PSO network with $N = 1024$ nodes in panel (h).

the case of the unicodelang network it is actually the other way round, the optimisation of the p_s also increased the mapping accuracy by a small magnitude. Furthermore, in most panels we can observe a sudden drop in the mapping accuracy under the first few epochs which is followed by a slow increase and then a saturation in the curve. The plausible explanation for this behaviour is that the optimisation drives the network from the initial local optimum to another one.

Finally, according to Fig. S6., the mapping accuracy tends to be either increasing, or remains more or less constant when we start the simulated annealing from a random initial embedding.

S3.2 Quality scores related to graph reconstruction

The graph reconstruction problem can be viewed as a task designed to examine to what extent is it possible to distinguish between connected and un-connected node pairs based on the geometric distance between their endpoints. Intuitively, we may imagine deleting all links from the embedded network, and then trying to reconstruct the graph by connecting L number of node pairs based on the connection probability given by the hyperbolic coordinates (where L is equal to the initially existing number of links). Since the connection probability is a monotonously decreasing function of the distance in hyperbolic networks, in practice this is equivalent to first ranking the node pairs according to their hyperbolic distance, and then connecting the L node pairs that appear at the top of the list.

In a more general setting, the number of "reconstructed" links does not have to match the actually existing number of links in the system, and instead it can serve as a parameter that can be changed starting from 0 up to the maximum possible number of links given by $N(N-1)/2$ in a network with N. In such a process, the receiver operating characteristic curve (ROC) is defined by plotting the ratio between the number of correctly reconstructed links and the number of existing links in the original network as a function of the ratio between the number of all reconstructed links (both correct and "false positive") and the maximum number of possible links[4,5]. The ROC always starts at $[0,0]$ and ends at $[1,1]$, and for random prediction takes the form of a simple straight line between these endpoints. To summarise the behaviour of the receiver operating characteristic curve it is a standard practice to take the area under the ROC, which is usually denoted by AUROC $\in [0,1]$, as was done in previous studies of graph reconstruction and link prediction[5,6]. (The AUROC value of a simple random predictor takes the value of AUROC=$1/2$).

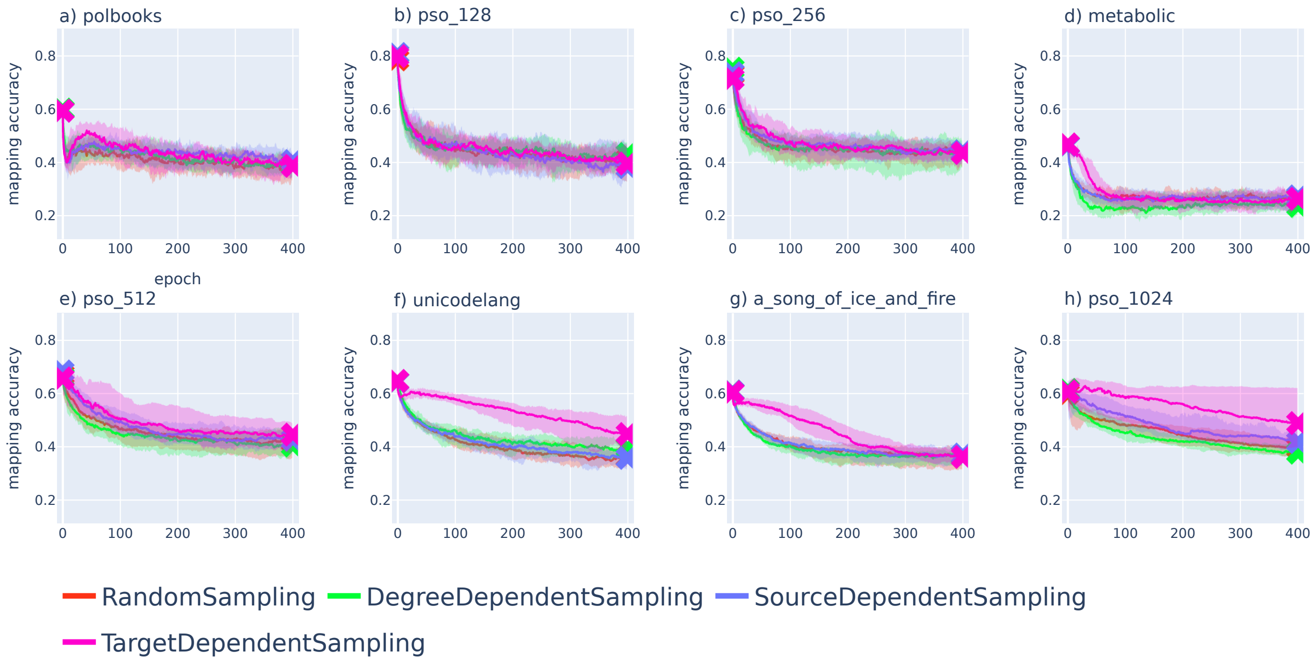

Figure S4. The median mapping accuracy over 20 instances as a function of the number of epochs during the optimisation when the initial embedding was obtained with Mercator[2]. The colour indicates the annealing scheme, the shaded region around the curves falls between the 40th and 60th percentiles and the network is indicated in the panel title.

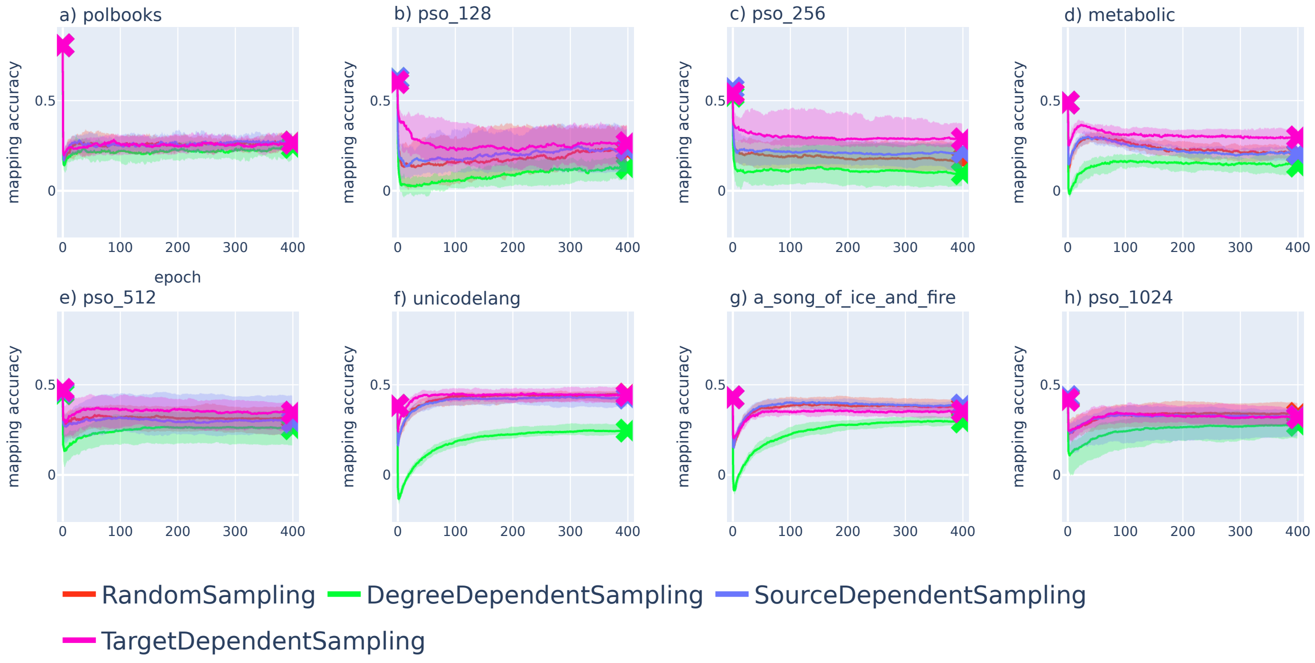

Figure S5. The median mapping accuracy over 20 instances as a function of the number of epochs during the optimisation when the initial embedding was obtained with hyperbolic ISOMAP[1]. The colour indicates the annealing scheme, the starting and ending values are marked by 'x' symbols, the shaded region around the curves falls between the 40th and 60th percentiles and the network is indicated in the panel title.

In Fig. S7. we show the AUROC values observed when optimising embeddings obtained with Mercator[2], whereas Figs. S8-S9. display the results when we start the annealing procedure from hyperbolic ISOMAP[1] and random embeddings, respectively.

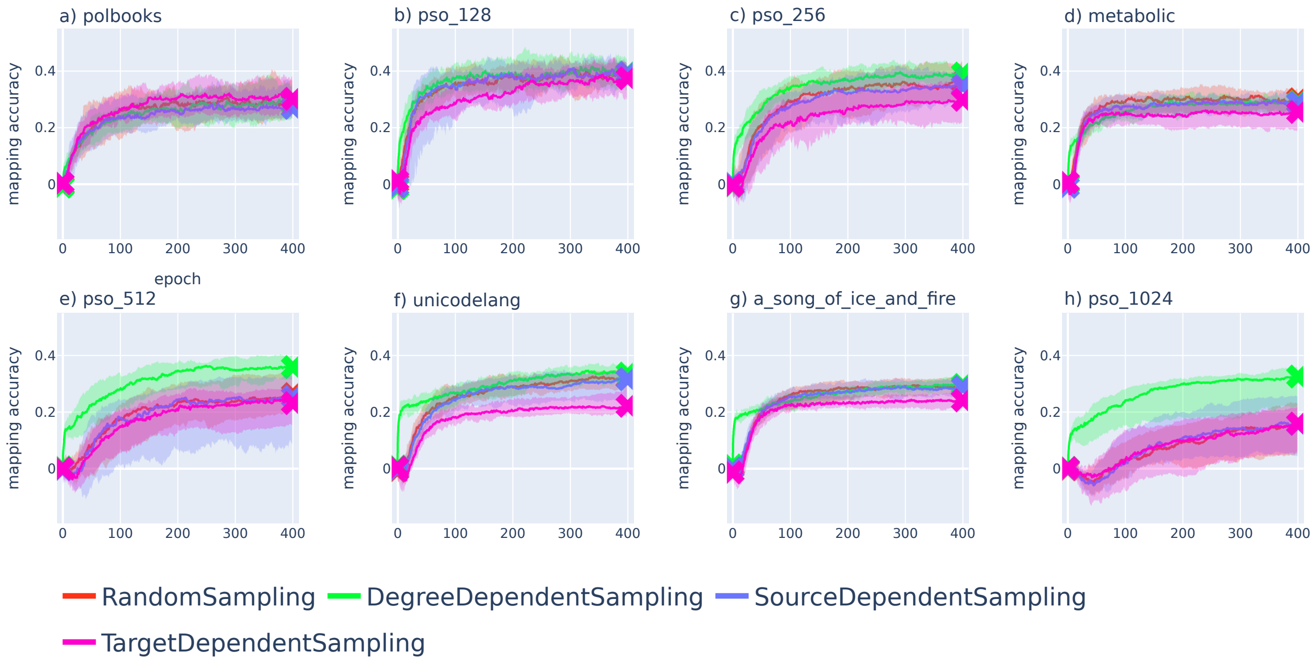

Figure S6. The median mapping accuracy over 20 instances as a function of the number of epochs when the optimisation starts from a random embedding. The colour indicates the annealing scheme, the starting and ending values are marked by 'x' symbols, the shaded region around the curves falls between the 40th and 60th percentiles and the network is indicated in the panel title.

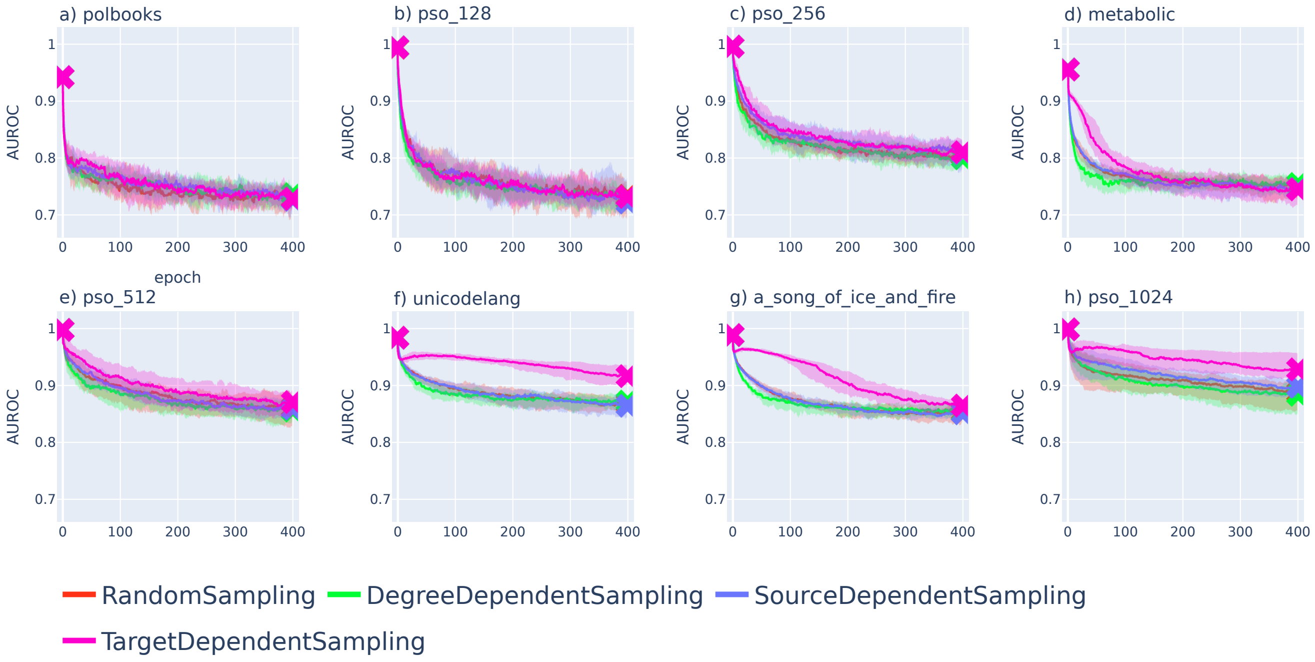

Figure S7. The median AUROC over 20 instances as a function of the number of epochs when the optimisation starts from an embedding obtained with Mercator[2]. The colour indicates the annealing scheme, the starting and ending values are marked by 'x' symbols, the shaded region around the curves falls between the 40th and 60th percentiles and the network is indicated in the panel title.

Similarly to the mapping accuracy in Fig. S4., the AUROC shows a decreasing tendency for all networks in Fig. S7. This again

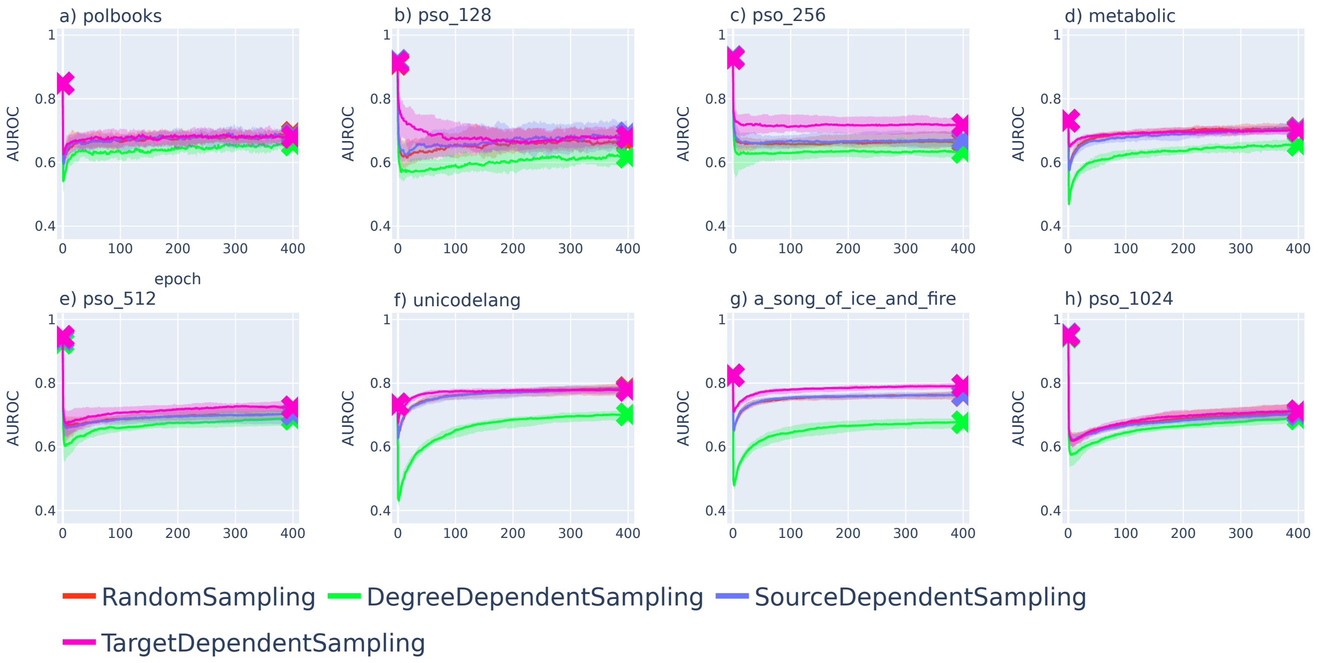

Figure S8. The median AUROC over 20 instances as a function of the number of epochs when the optimisation starts from an embedding obtained with hyperbolic ISOMAP[1]. The colour indicates the annealing scheme, the starting and ending values are marked by 'x' symbols, the shaded region around the curves falls between the 40th and 60th percentiles and the network is indicated in the panel title.

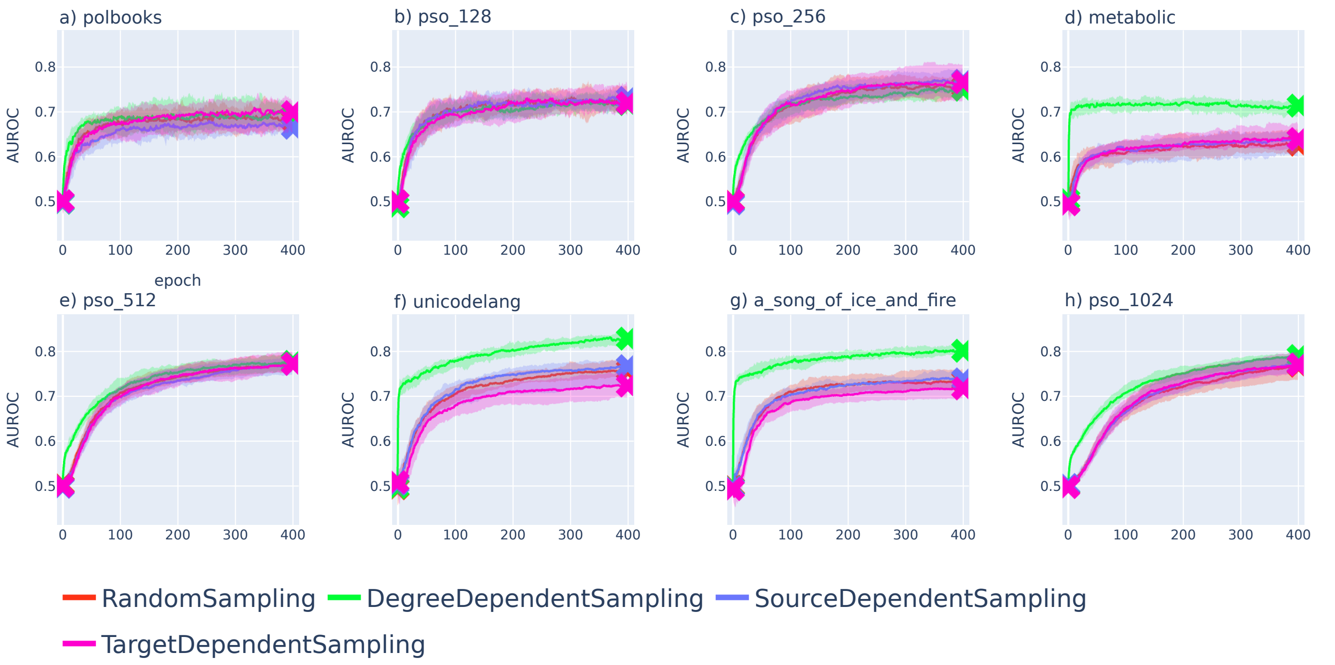

Figure S9. The median AUROC over 20 instances as a function of the number of epochs when the optimisation starts from a random embedding. The colour indicates the annealing scheme, the starting and ending values are marked by 'x' symbols, the shaded region around the curves falls between the 40th and 60th percentiles and the network is indicated in the panel title.

shows that Mercator[2] generates embeddings with high quality scores, and as our algorithm is optimising solely for the success

ratio, the value of other quality indicators is likely to decrease when applying our framework on the output by Mercator. In contrast, the curves have almost always an increasing nature in Fig. S9., indicating that compared to a fully random layout that is agnostic to the geometry, our simulated annealing procedure can achieve a better configuration even according to AUROC value, in spite that it is not designed to optimise this score. The results for optimisations starting from a hyperbolic ISOMAP[1] embedding in Fig. S8. are similar to Fig. S5.: In the case of the unicodelang network the optimisation slightly increased the AUROC score, whereas for the other networks it had the opposite effect, although the overall change was in most cases only minor in magnitude.

A further quality of interest in graph reconstruction is given by the precision, defined as the fraction of the correctly predicted existing links among all reconstructed links[6]. Similarly to the ROC, we can also plot the precision as a function of the ratio between the number of restored links and the number of all possible node pairs, resulting in the so-called precision-recall curve. For a random predictor, this curve is corresponding to a constant curve at a value given by $\frac{2L}{N(N-1)}$, where L is the number of links and N is the number of nodes in the original network. The area under the precision-recall curve AUPR $\in (0,1]$ provides a further important measure that can be used for quantifying the quality of a network embedding[6].

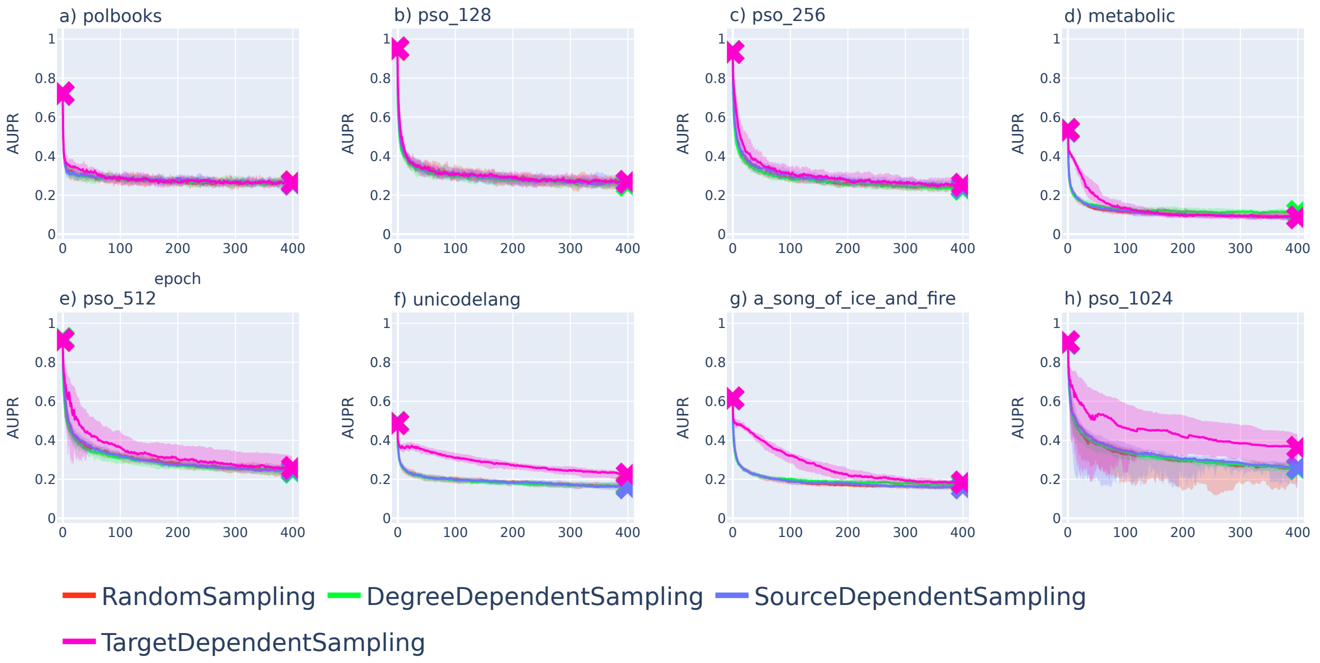

Figure S10. The median AUPR over 20 instances as a function of the number of epochs when the optimisation starts from an embedding obtained with Mercator[2]. The colour indicates the annealing scheme, the starting and ending values are marked by 'x' symbols, the shaded region around the curves falls between the 40th and 60th percentilea and the network is indicated in the panel title.

In Fig. S10. we display the AUPR when starting the simulated annealing from embeddings obtained with Mercator[2]. Similarly to the AUROC score, we can observe a significant decrease in all of the cases, showing that our optimisation can have disadvantageous side affects when approaching the quality of an embedding from multiple perspectives. In Fig. S11. we show the analogous results for the AUPR when the optimisation was started from embeddings obtained with hyperbolic ISOMAP[1]. Here the picture is mixed, where for some networks such as the PSO graphs of different sizes we see a decreasing tendency and in parallel, for other systems such as the unicodelang network or the network between fictional characters we can observe a slightly increasing tendency. This indicates that for hyperbolic ISOMAP embeddings our optimisation framework focusing on the success rate can have a positive effect also on the AUPR. The AUPR results for optimisation experiments starting from random initial coordinates are presented in Fig. S12. These plots show increasing AUPR curves, achieving a notable improvement in some of the cases. We note that similarly to the results related to the greedy congruence in Fig. S3., the different sampling methods may lead to different performance in terms of the AUROC and AUPR scores as well in some of the networks.

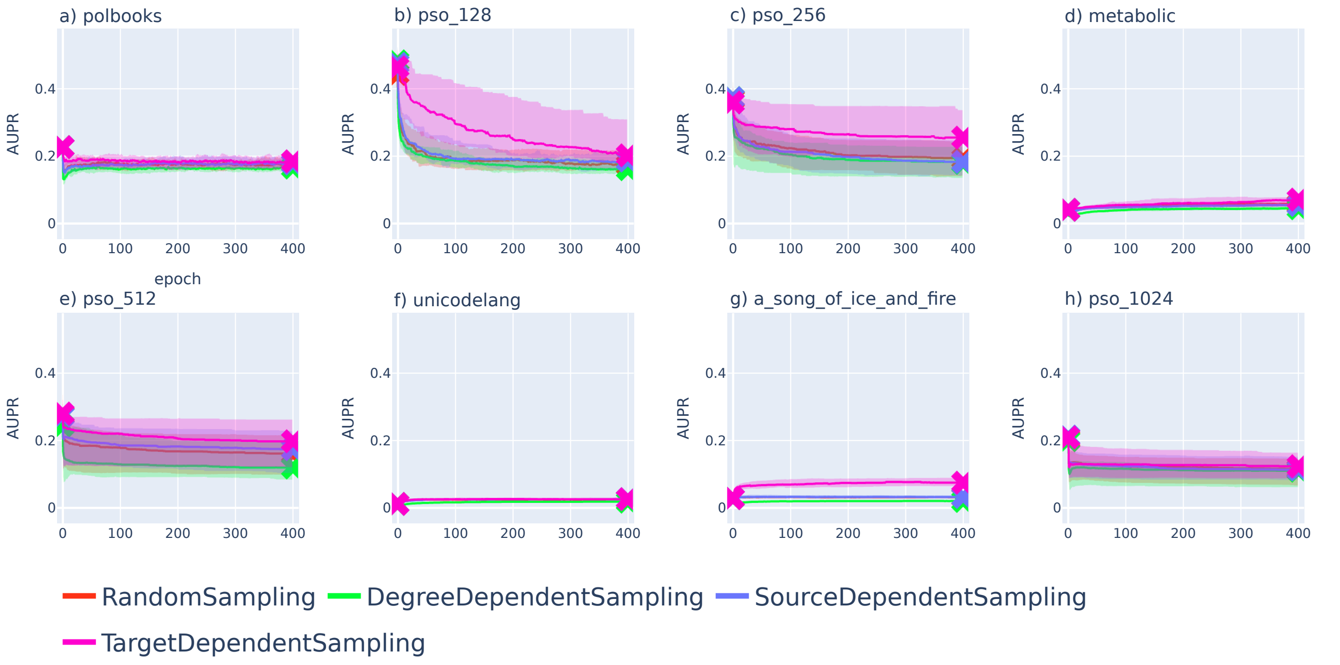

Figure S11. **The median AUPR over 20 instances as a function of the number of epochs when the optimisation starts from an embedding obtained with hyperbolic ISOMAP[1].** The colour indicates the annealing scheme, the starting and ending values are marked by 'x' symbols, the shaded region around the curves falls between the 40th and 60th percentilea and the network is indicated in the panel title.

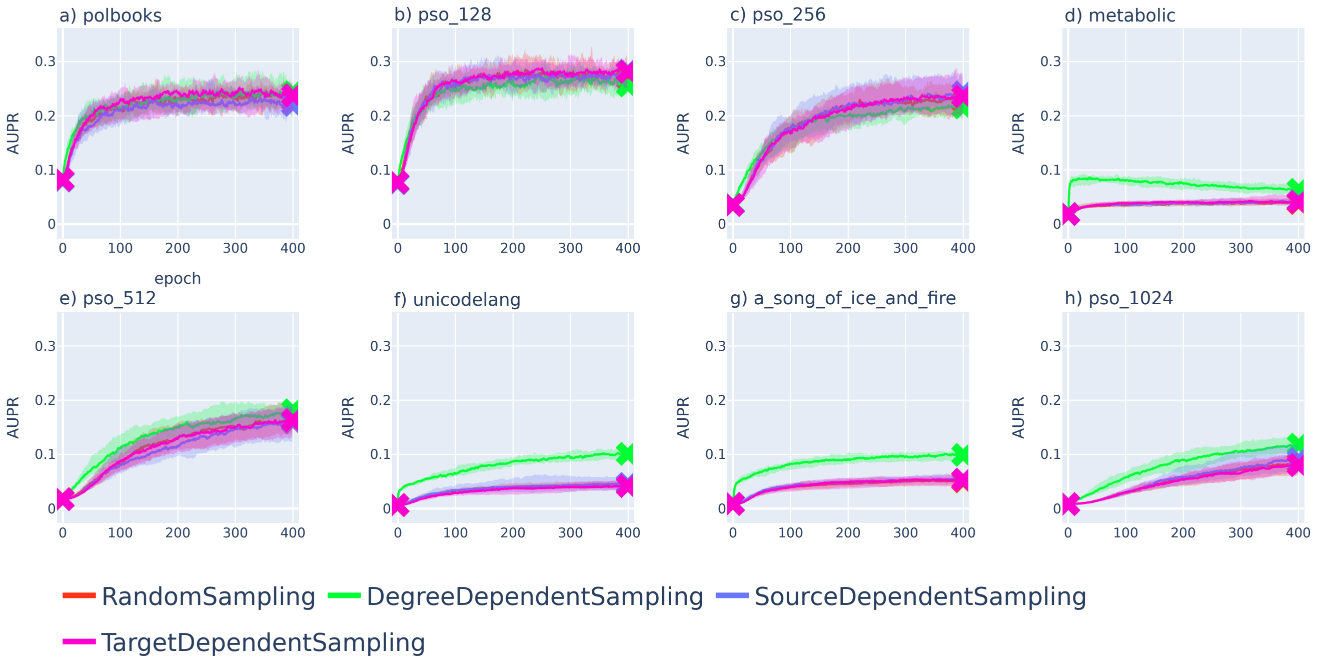

Figure S12. **The median AUPR over 20 instances as a function of the number of epochs when the optimisation starts from a random embedding.** The colour indicates the annealing scheme, the starting and ending values are marked by 'x' symbols, the shaded region around the curves falls between the 40th and 60th percentiles and the network is indicated in the panel title.

S3.3 Quality scores related to greedy routing

Besides the success ratio studied in the main paper, further quality measures can be defined for quantifying the greedy navigability of networks embedded in geometric spaces. An important example is given by the greedy routing score[1], as defined

in Eq.(1) in the main paper. Instead of focusing only on the fraction of the successful greedy paths, this measure incorporates information about the lengths of the paths as well. In Fig. S13. we show the behaviour of this score when the simulated annealing scheme starts from an embedding obtained with Mercator[2]. Interestingly, for the studied real networks the GR-score is improving during the optimisation, whereas in the case of the PSO networks it is either constant, or becomes constant after a small drop at the beginning of the simulated annealing process. In parallel, according to Figs. S14-S15, all curves show an

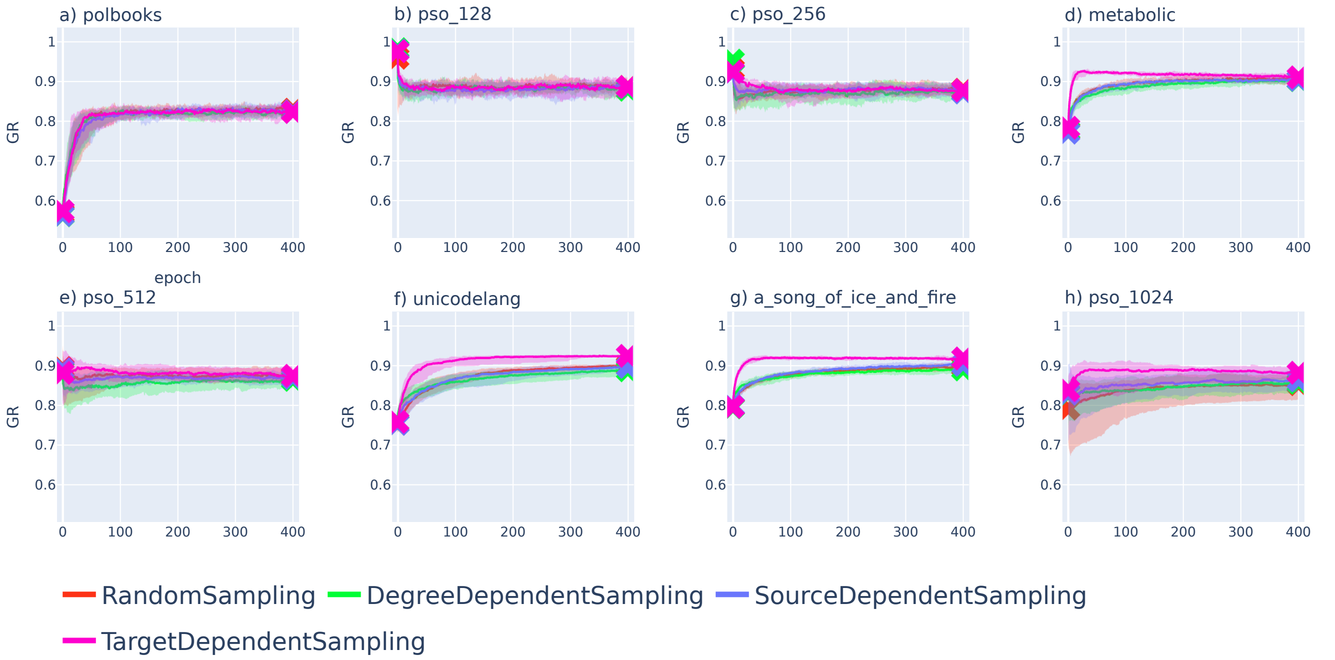

Figure S13. The median of the greedy routing score, GR, over 20 instances as a function of the number of epochs when the optimisation starts from an embedding obtained with Mercator[2]). The colour of the curves encode the annealing scheme, the starting and ending values are marked by 'x' symbols, the shaded region around the curves falls between the 40th and 60th percentiles and the network is indicated in the panel title.

increasing GR-score when the optimisation is started from the output of hyperbolic ISOMAP[1] or a random embedding, where in the latter case a major improvement can be observed for all networks during the simulated annealing process. The likely reason behind this is that the GR-score and p_s are closely related, and when optimising a random initial state with respect to p_s, most of the implemented displacements increase the GR-score as well.

In addition, a third quality score that was proposed to quantify the greedy navigability of a network is given by the greedy routing efficiency[7], comparing the geometric distances and the projected greedy routing paths. This measure can be formulated as

$$GE(\{r_i,\theta_i\}) = \frac{1}{N(N-1)-L}\sum_{i=1}^{N}\sum_{\substack{j=1\\ j\neq i\\ j\notin N(i)}}^{N}\frac{DIST(i,j)}{PGRP(i,j)}, \tag{S1}$$

where the summation runs over all nonadjacent pair of nodes, $DIST(i,j)$ denotes the geometric distance between i and j, $PGRP(i,j)$ is corresponding to the length of the projected greedy routing path between the same pair of nodes, N gives the total number of nodes and L stands for the number links in the network.

In Fig. S16. we present the results for GE in simulated annealing experiments starting from embeddings obtained with Mercator[2], in Fig. S17. we show the same results obtained when starting from embeddings according to the hyperbolic ISOMAP[1], whereas Fig. S18. depicts the analogous results obtained for random initial embeddings. The curves are showing an increasing tendency in all experiments, and a considerable improvement can be observed in most of the cases over the iterations. This means that our optimisation framework usually finds spatial configurations that are more advantageous not only from the point of view of the success of the greedy paths, but also in terms of the efficiency. A likely reason for this effect is that unsuccessful paths have a zero contribution to the sum in Eq. (S1) and making them successful is increasing the GE.

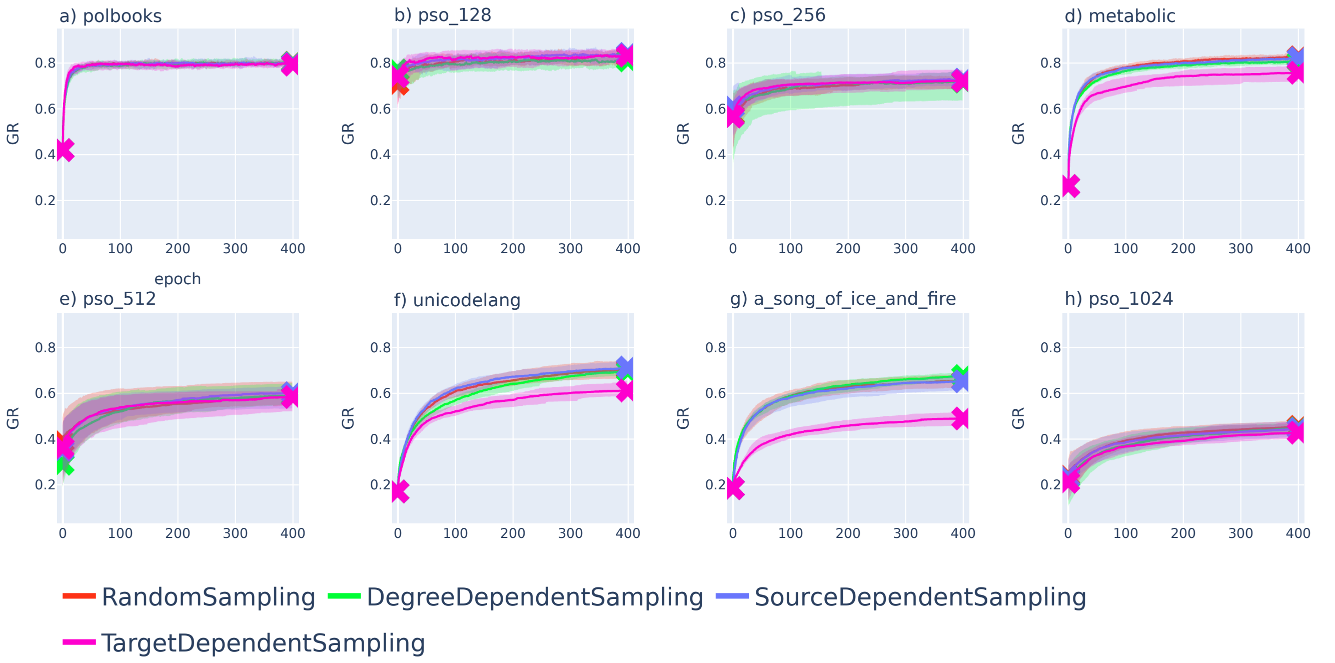

Figure S14. The median of the greedy routing score, GR, over 20 instances as a function of the number of epochs when the optimisation starts from an embedding obtained with hyperbolic ISOMAP[1]). The colour of the curves encode the annealing scheme, the starting and ending values are marked by 'x' symbols, the shaded region around the curves falls between the 40th and 60th percentiles and the network is indicated in the panel title.

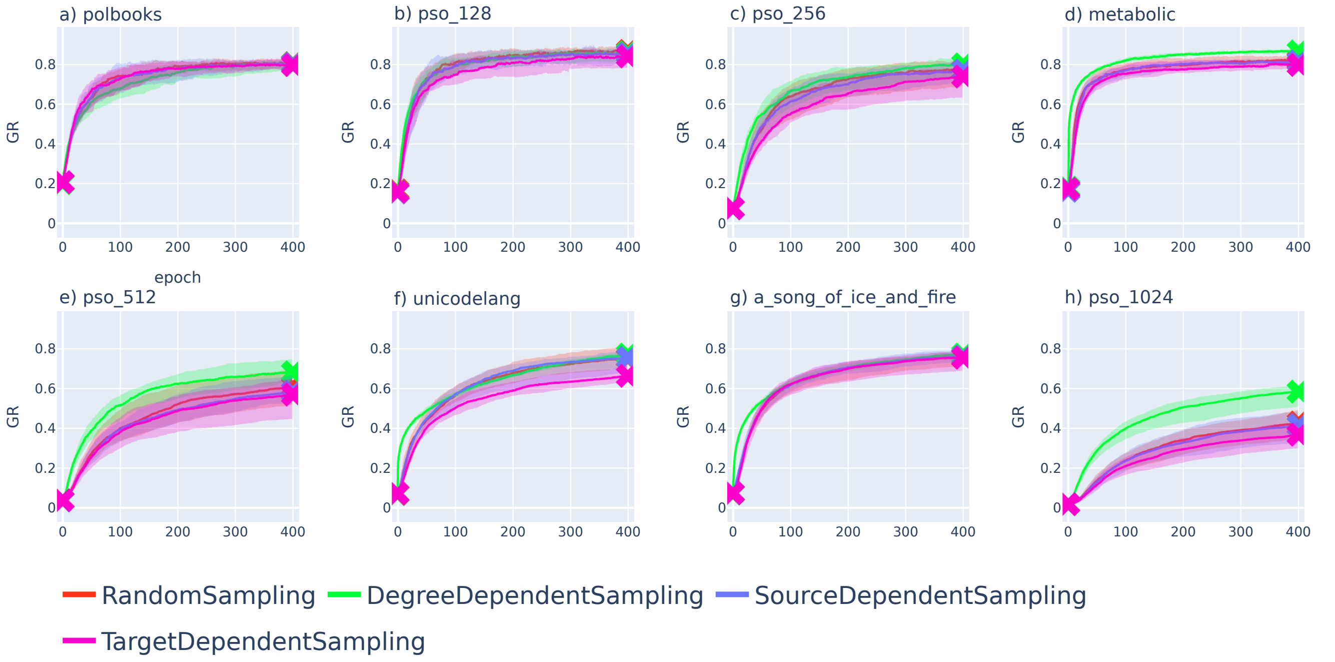

Figure S15. The median GR over 20 instances as a function of the number of epochs when the optimisation starts from a random embedding. The colour indicates the annealing scheme, the starting and ending values are marked by 'x' symbols, the shaded region around the curves falls between the 40th and 60th percentiles and the network is indicated in the panel title.

Finally, when comparing the performance results for the different sampling methods in terms of the scores related to greedy routing, again, clear separation of the curves can be observed for some of the networks. By taking together Figs. S3.,S9., S12.

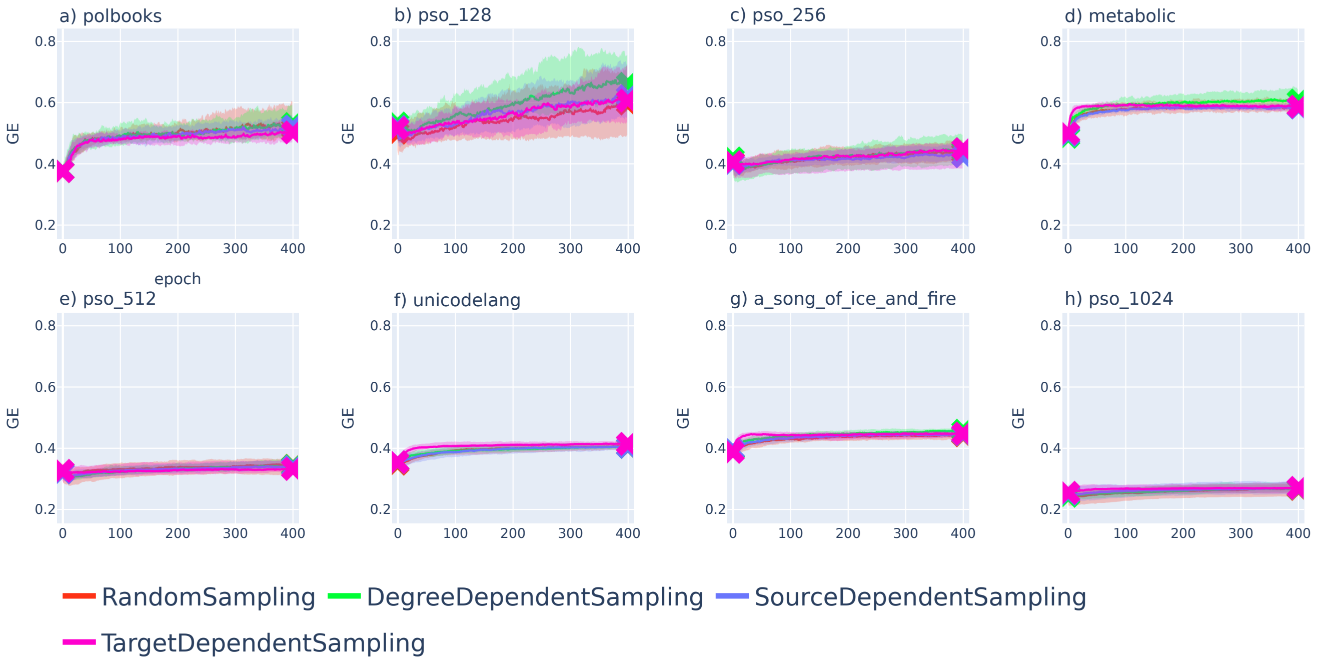

Figure S16. The median of GE over 20 instances as a function of the number of epochs when the optimisation starts from an embedding obtained with Mercator[2]). The colour of the curves encode the annealing scheme, the starting and ending values are marked by 'x' symbols, the shaded region around the curves falls between the 40th and 60th percentiles and the network is indicated in the panel title.

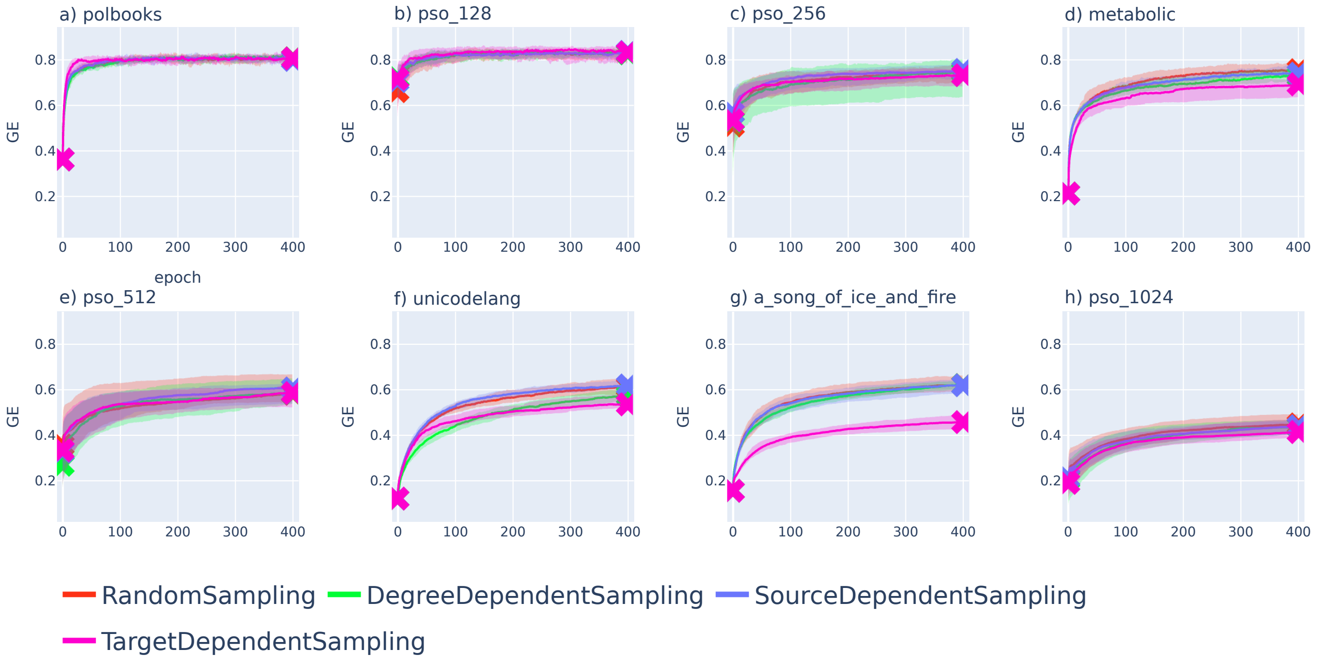

Figure S17. The median of GE over 20 instances as a function of the number of epochs when the optimisation starts from an embedding obtained with hyperbolic ISOMAP[1]). The colour of the curves encode the annealing scheme, the starting and ending values are marked by 'x' symbols, the shaded region around the curves falls between the 40th and 60th percentiles and the network is indicated in the panel title.

and Fig. S18., it seems that the degree based sampling can achieve somewhat better results according to some of the quality

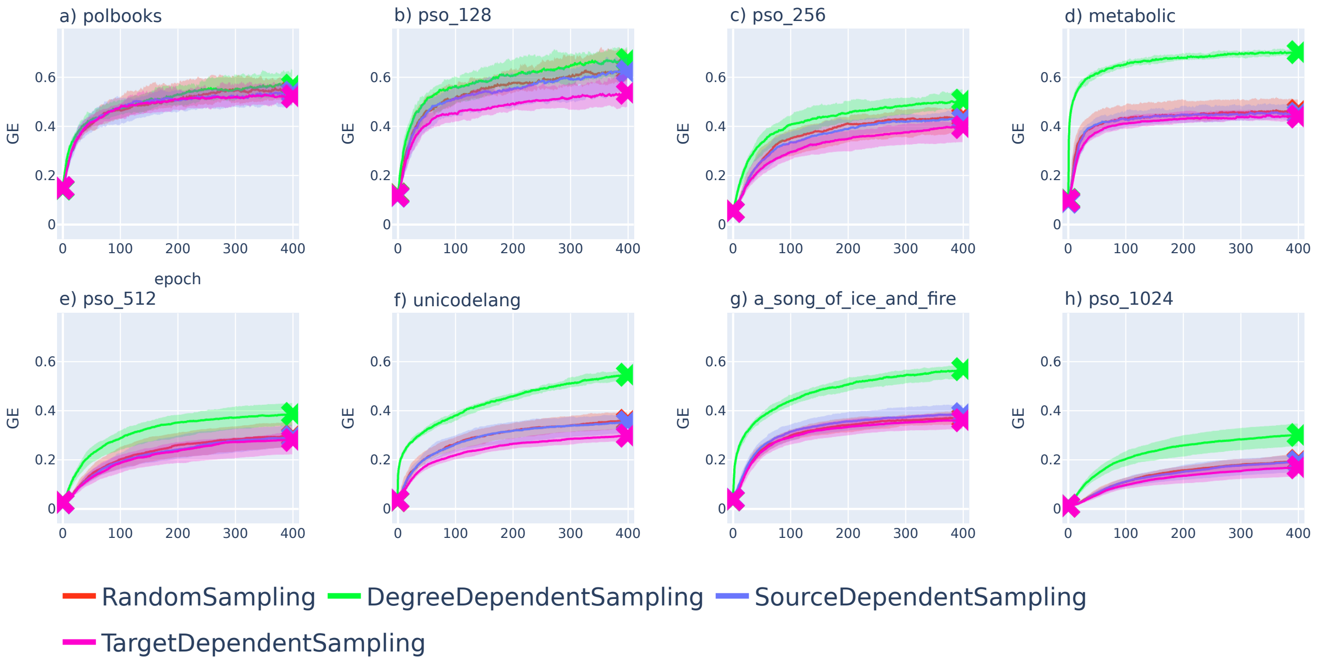

Figure S18. The median GE over 20 instances as a function of the number of epochs when the optimisation starts from a random embedding. The colour indicates the annealing scheme, the starting and ending values are marked by 'x' symbols, the shaded region around the curves falls between the 40th and 60th percentiles and the network is indicated in the panel title.

scores for the metabolic, unicodelang and fictional character networks when the optimisation is started from random initial coordinates. In contrast, according to Figs. S5., S8. and S11., when starting from an embedding according to the hyperbolic ISOMAP[1], it is the target-based sampling that seems to provide a faster increase for some of the scores for the same networks. These effects are likely to be the result of a particular interplay between the specific structure of these networks, the annealing framework and the nature of the given quality measure.

S4 Annealing with partially fixed coordinates

According to the results discussed in the main paper and also in previous sections of the Supplementary Information, the degree based sampling of the nodes during the optimisation can surpass other sampling methods by large according to some of the quality scores in a number of cases. To investigate this effect further, we implemented annealing experiments where the position of nodes falling in user specified degree ranges was fixed, and only the rest of the network was allowed to be reorganised.

More specifically, during these experiments we fixed the position of the top 5% of the nodes according to the degree, corresponding to the "hubs", where the sampling of the nodes was random otherwise. For these studies we used only Mercator and random embedding as the initial state for the annealing procedure, and left out ISOMAP embeddings for simplicity.

In Figs. S19-S20. we show the optimisation results for the success rate when starting the procedure from Mercator and random embeddings, respectively. These figures are basically repetitions of Fig.4. in the main paper and Fig.S2., where a new sampling method (with fixed hub positions, indicated by the gray colour) has been added. According to the results in Figs.S19-S20, the performance of the optimisation procedure is strongly affected by the fixed hub positions in the case of a random initial embedding, but not to a large extent for a Mercator embedding. The likely reason behind this is that the hubs are already placed in their optimal positions for most Mercator embeddings, and thus, there is no need to relocate them in that case.

In terms of the further quality scores, the results for the geometrical congruence are shown in Figs. S21-S22., for the mapping accuracy in Figs.S23-S24., for the AUROC in Figs. S27-S28., for the AUPR in Figs. S27-S28., for the GR in Figs. S29-S30. and for the GE in Figs. S31-S32. These show a similar behaviour compared to the success rate in that the quality score curves for the sampling with fixed hubs and complete random sampling are nearly identical when starting the optimisation from Mercator embeddings, whereas for random initial node positions, the curves for annealing with fixed hubs is usually below the curve of any other sampling method.

The above results indicate that the position of the hubs can strongly affect the evolution of the different quality scores during the optimisation procedure, since by fixing only 5% of the nodes, the quality scores measured at termination can drop by

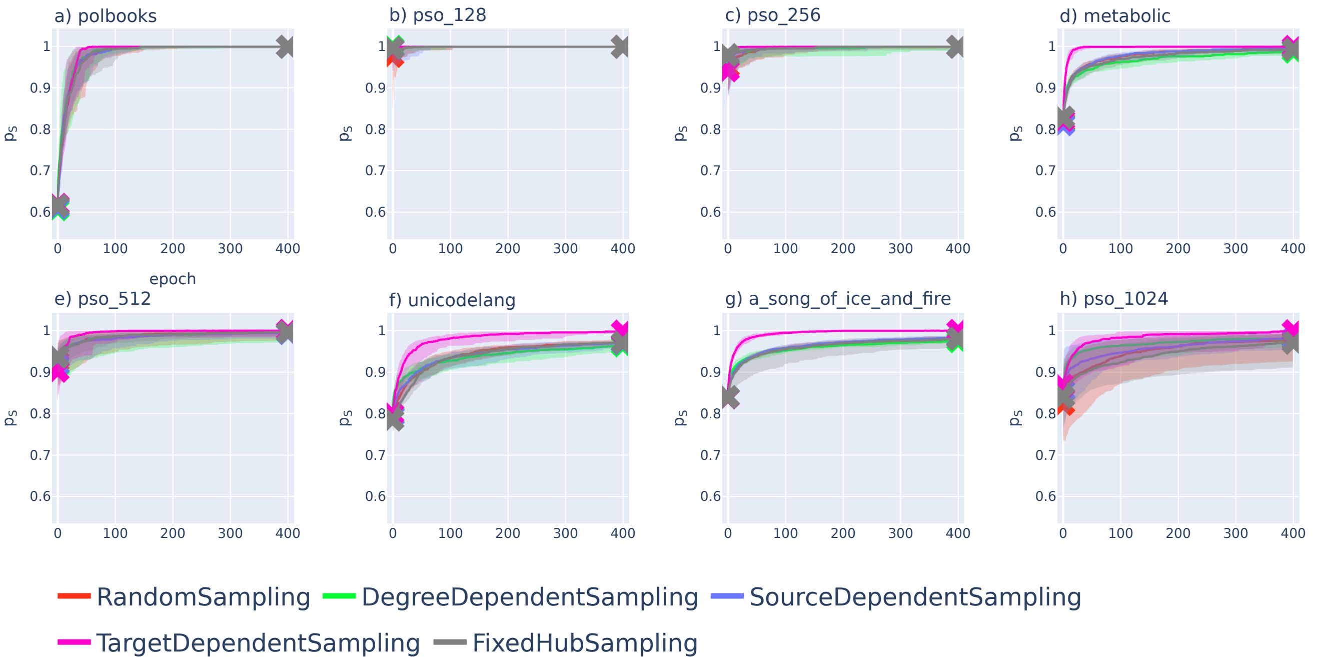

Figure S19. Improvement of p_s for Mercator embeddings with fixed hubs. We plot the median over 20 instances for the success rate as a function of the number of epochs, where the results for sampling with fixed hubs is shown in gray. The starting and ending values are marked by 'x' symbols and the shaded region around the curves falls between the 40th and 60th percentiles.

15-20% in some cases. However, this aspect is mostly relevant for random initial embedding, where hubs are placed at random. In contrast, Mercator seems to place the hubs optimally in the sense that their fixation does not really effect the quality achieved by the optimisation.

S5 Violin plots for the quality measures

In this Section we also provide an alternative visualisation for the quality scores measured during the optimisation procedure using violin plots. By taking the value of any studied quality measure at the end of the optimisation procedure in the separate experiments, we can define an empirical probability distribution. When displaying this distribution vertically (using a kernel density technique), the resulting form usually resembles to (a part of) the body of a violin. For comparison, in each plot we also show the distribution of the studied quality measure at the beginning of the optimisation procedure. This way it becomes clear from the plots to what extent did the different quality measures change during the annealing.

In Figs.S33-S39. we show p_s, the GC, the mapping accuracy, the AUROC, the AUPR, the GR and the GE for optimisation procedures starting from Mercator[2] embeddings. Similarly, in Figs.S40-S46. we display the same scores for optimisation procedures starting from hyperbolic ISOMAP[1] embeddings. Please note that since this is a fully deterministic embedding, for real networks the distribution of any quality measure at the beginning of the optimisation falls onto a single point. (In the case of PSO networks, the different samples of the generated networks allow the formation of a distribution also for the initial quality score values). Finally, in Figs.S47-S53. we show the analogous results for random embeddings.

S6 Reconstructed graphs from the optimised embedding

A further interesting question is whether different statistical properties of graphs generated based on the embeddings match that of the original networks which were embedded. Here we examine this in a framework similar to the graph reconstruction studies mentioned in Sect.S3.2.

Since Mercator is embedding according to the $\mathbb{S}^1/\mathbb{H}^2$ model[2] (also called as the Random Hyperbolic Graph, RHG), it is natural to define linking probabilities according to this approach. Here for a node pair i and j with coordinates (r_i, θ_i) and

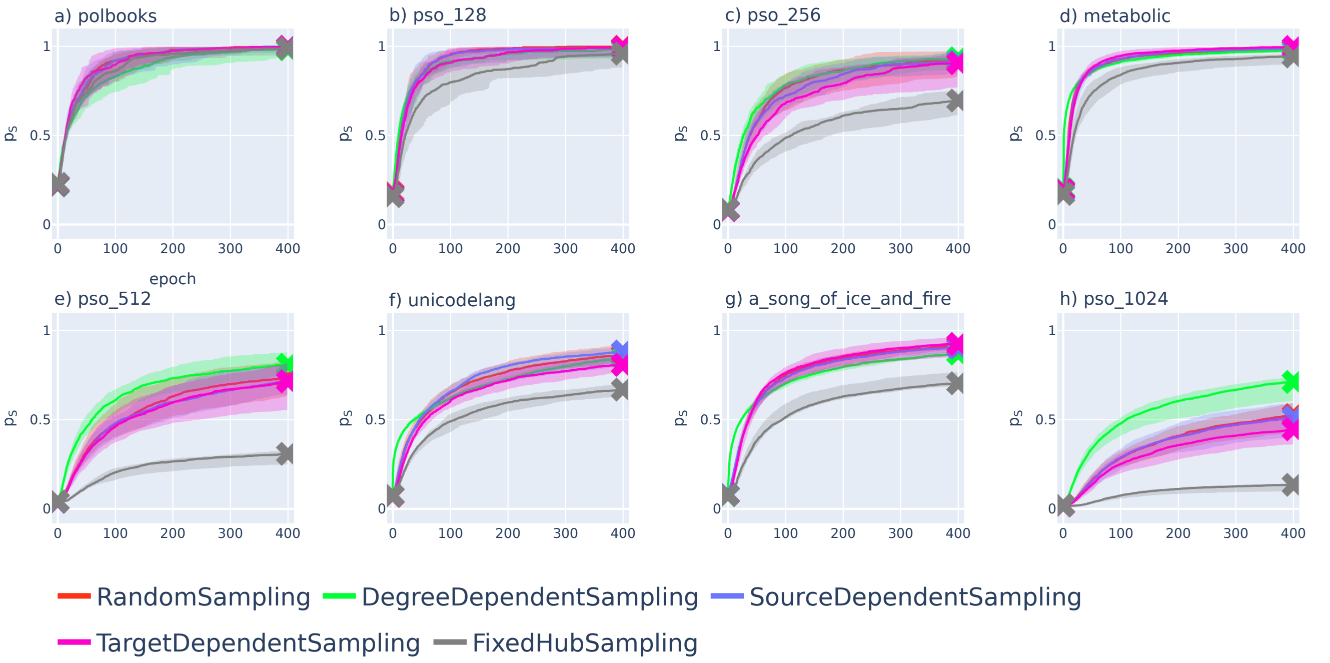

Figure S20. Improvement of p_s for random embeddings with fixed hubs. We plot the median over 20 instances for the success rate as a function of the number of epochs, where the results for sampling with fixed hubs is shown in gray. The starting and ending values are marked by 'x' symbols and the shaded region around the curves falls between the 40th and 60th percentiles.

(r_j, θ_j) the connection probability is formulated with the help of hidden degrees κ_i and κ_j as[2]

$$p_{ij} = \frac{1}{1 + \left(\frac{N \cdot \Delta\theta_{ij}}{2\pi \cdot \mu \cdot \kappa_i \cdot \kappa_j}\right)^{\alpha}}, \tag{S2}$$

where α is a model parameter, $\Delta\theta_{ij} = \pi - |\pi - |\theta_i - \theta_j||$ is the angular distance between the nodes, the constant $\mu = \frac{\alpha}{2\pi\langle k\rangle} \cdot \sin\left(\frac{\pi}{\alpha}\right)$ is controlled by the average degree $\langle k\rangle$. The hidden degree κ_i is coupled with the radial coordinate of node i according to

$$r_i = \hat{R} - 2\ln\left(\frac{\kappa_i}{\kappa_0}\right), \tag{S3}$$

where $\hat{R} = 2\ln\left(\frac{N}{\mu\pi\kappa_0^2}\right)$ with the κ_0 corresponding to the minimal possible hidden degree. Since Mercator[2] provides information also on the parameters of the fitted $\mathbb{S}^1/\mathbb{H}^2$ model aside the node coordinates, for each studied network, we used the parameters obtained from the output of Mercator during the reconstruction process.

We applied the same procedure also for optimised embeddings that were started from coordinates obtained from Mercator. The argument behind this is that in most cases our algorithm implements only minor modifications on Mercator embeddings (as illustrated e.g., in Fig.3c-d in the main paper), hence it seems reasonable to treat these embeddings as 'distorted' Mercator embeddings, and therefore, generate links again according to the $\mathbb{S}^1/\mathbb{H}^2$ model.

In Fig.S54. we compare the degree distribution of the studied networks (shown in red) with the degree distribution of the reconstructed graphs according to the Mercator embedding (coloured green) and according to the embedding obtained from the annealing procedure that started from the Mercator embedding (coloured ble). In general, the $p(k)$ of the reconstructed graphs based on Mercator embeddings follows the original degree distribution quite closely for real-world networks. In the case of PSO networks, we can observe some deviation that seems to increase slightly with the system size. In the case of networks in the bottom row of Fig.S54 (i.e., Figs.S54e-h), the $p(k)$ for the graphs reconstructed from optimised embeddings is very similar to the $p(k)$ of reconstructions based on Mercator embeddings, thus, for these networks the optimisation procedure had only a minor effect on the $p(k)$ we can gain from reconstructed networks. On the other hand, for networks in the top-row of Fig.S54

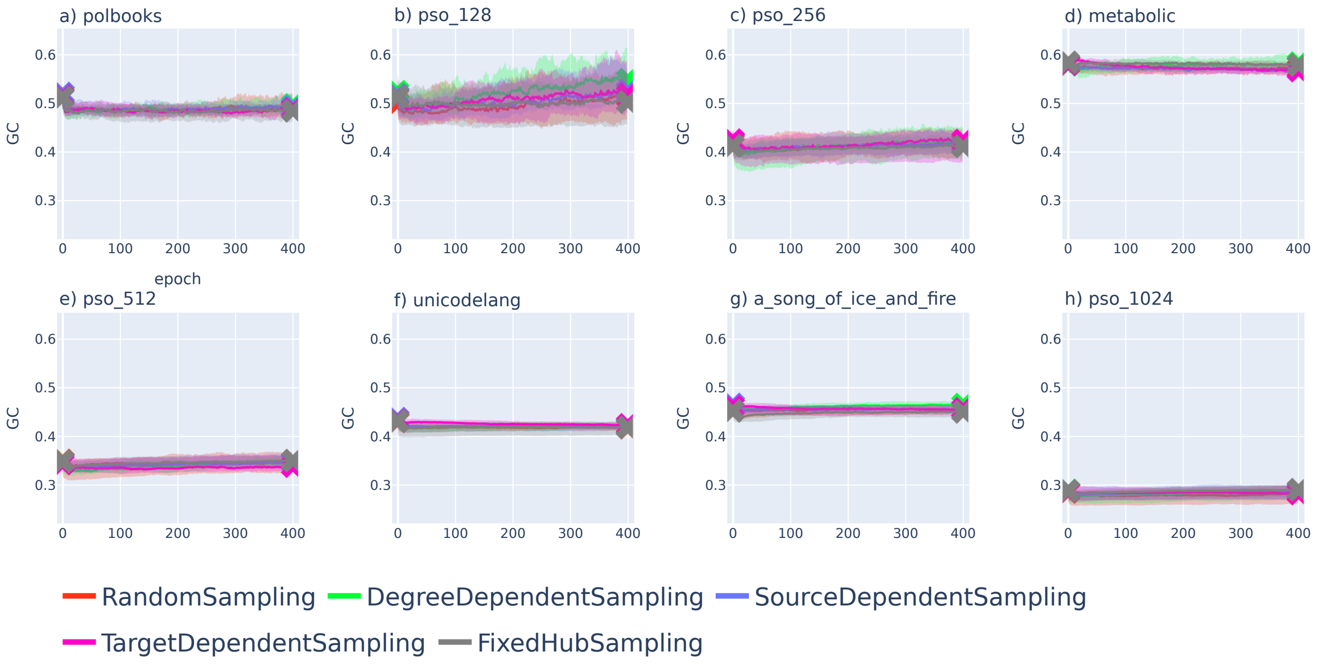

Figure S21. Change in the geometrical congruence for Mercator embeddings with fixed hubs. We plot the median over 20 instances for the success rate as a function of the number of epochs, where the results for sampling with fixed hubs is shown in gray. The starting and ending values are marked by 'x' symbols and the shaded region around the curves falls between the 40th and 60th percentiles.

(i.e., Figs.S54a-d), the $p(k)$ for the reconstructed networks based on the optimised embeddings is clearly further away from the original $p(k)$ compared to reconstructions based on Mercator embeddings.

In Table S1 we show the average clustering coefficient, $\langle C \rangle$ for the same networks that are studied in Fig.S54. The average deviation between $\langle C \rangle$ measured in the original networks and in the reconstructed graphs based on Mercator embeddings is 0.070, which shows that these reconstructions are quite close to the original networks from this aspect. In parallel, the average of the absolute difference in $\langle C \rangle$ when comparing the original networks with reconstructions using optimised coordinates is about 0.092. This is still quite low, nevertheless, we can see that the reconstructions based on the optimised coordinates slightly under perform the reconstructions based on the original Mercator embeddings.

In conclusion, the degree distribution and average clustering coefficient of the graphs reconstructed from Mercator-based embeddings match with that of the original networks quite well. For the reconstructed graphs based on the optimised coordinates, these statistical indicators are somewhat farther away from the original values, however, in most cases they still seem to be in an acceptable range. This effect of distancing from the original degree distribution and average clustering coefficient is yet another cost of the optimisation.

References

1. Muscoloni, A., Thomas, J. M., Ciucci, S., Bianconi, G. & Cannistraci, C. V. Machine learning meets complex networks via coalescent embedding in the hyperbolic space. *Nat. Commun.* **8**, 1615, DOI: 10.1038/s41467-017-01825-5 (2017).

2. García-Pérez, G., Allard, A., Serrano, M. Á. & Boguñá, M. Mercator: uncovering faithful hyperbolic embeddings of complex networks. *New J. Phys.* **21**, 123033, DOI: 10.1088/1367-2630/ab57d2 (2019).

3. Zhang, Y.-J., Yang, K.-C. & Radicchi, F. Systematic comparison of graph embedding methods in practical tasks. *Phys. Rev. E* **104**, 044315, DOI: 10.1103/PhysRevE.104.044315 (2021).

4. Hanley, J. A. & McNeil, B. J. The meaning and use of the area under a receiver operating characteristic (ROC) curve. *Radiology* **143**, 29–36 (1982).

5. Clauset, A., Moore, C. & Newman, M. E. J. Hierarchical structure and the prediction of missing links in networks. *Nature* **453**, 98–101, DOI: 10.1038/nature06830 (2008).

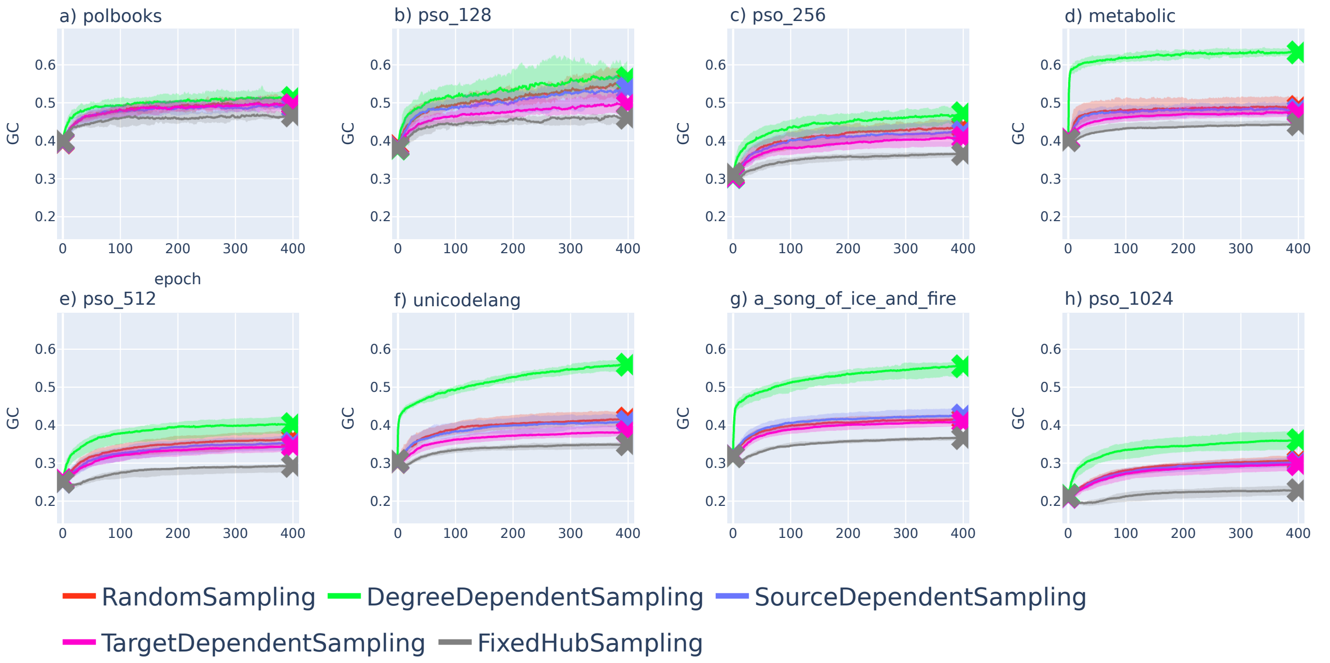

Figure S22. **Change in the GC for random embeddings with fixed hubs.** We plot the median over 20 instances for the success rate as a function of the number of epochs, where the results for sampling with fixed hubs is shown in gray. The starting and ending values are marked by 'x' symbols and the shaded region around the curves falls between the 40th and 60th percentiles.

6. Kitsak, M., Voitalov, I. & Krioukov, D. Link prediction with hyperbolic geometry. *Phys. Rev. Res.* **2**, 043113, DOI: 10.1103/PhysRevResearch.2.043113 (2020).

7. Cannistraci, C. V. & Muscoloni, A. Geometrical congruence, greedy navigability and myopic transfer in complex networks and brain connectomes. *Nat. Commun.* **13**, 7308, DOI: 10.1038/s41467-022-34634-6 (2022).

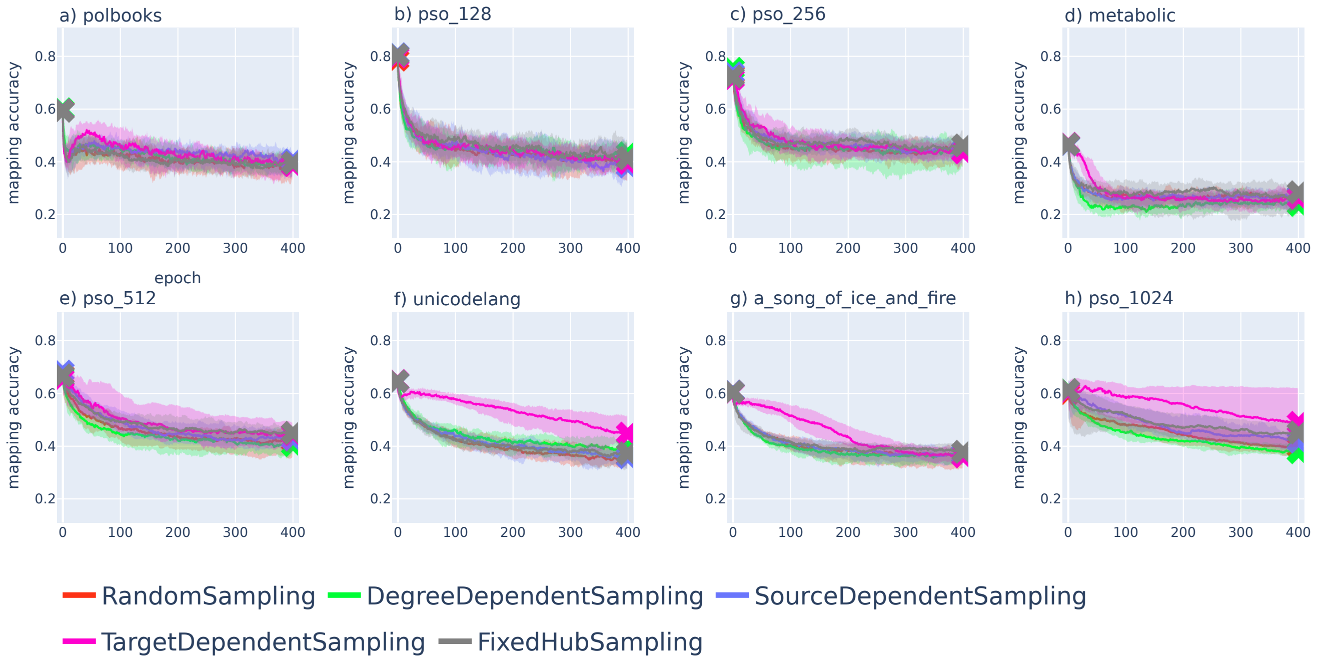

Figure S23. Change in the mapping accuracy for Mercator embeddings with fixed hubs. We plot the median over 20 instances for the success rate as a function of the number of epochs, where the results for sampling with fixed hubs is shown in gray. The starting and ending values are marked by 'x' symbols and the shaded region around the curves falls between the 40th and 60th percentiles.

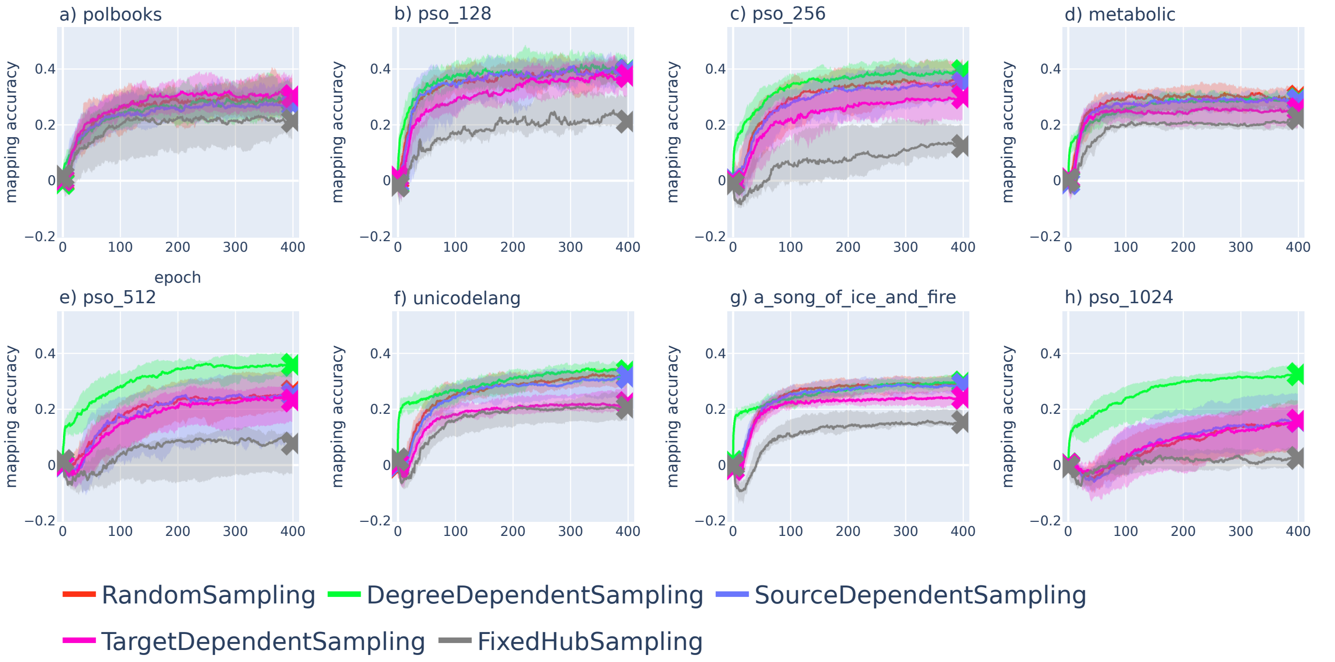

Figure S24. Change in the mapping accuracy for random embeddings with fixed hubs. We plot the median over 20 instances for the success rate as a function of the number of epochs, where the results for sampling with fixed hubs is shown in gray. The starting and ending values are marked by 'x' symbols and the shaded region around the curves falls between the 40th and 60th percentiles.

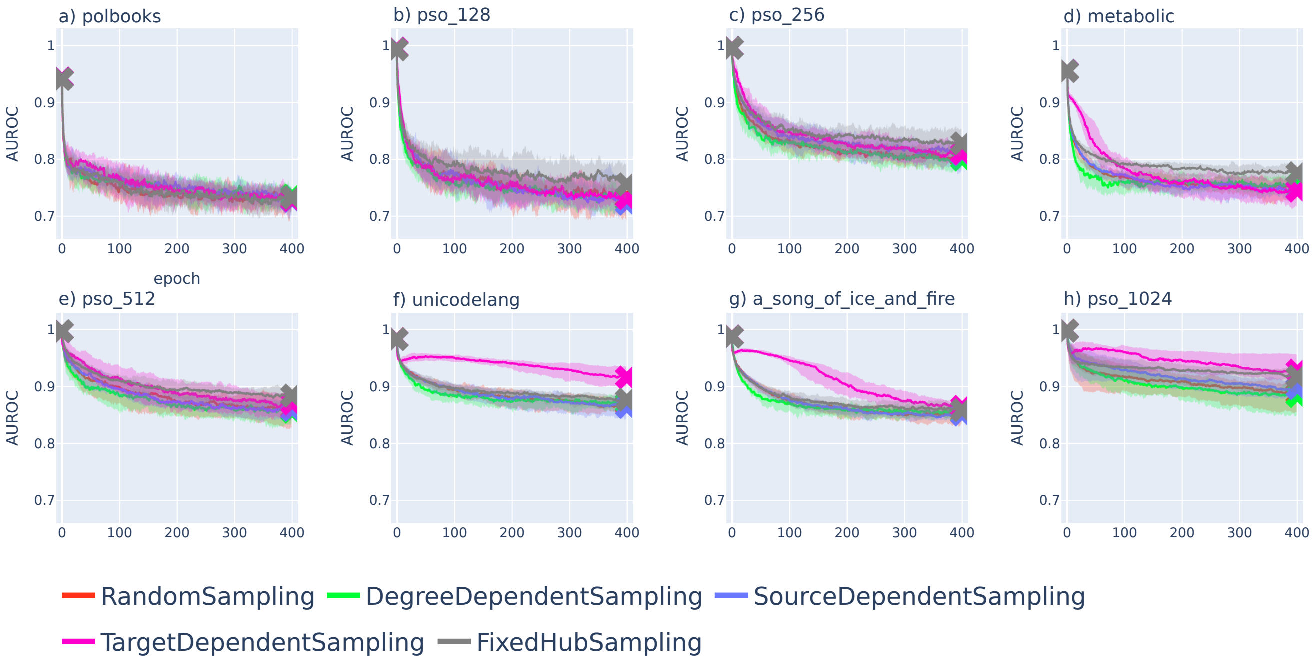

Figure S25. The AUROC for Mercator embeddings with fixed hubs. We plot the median over 20 instances for the success rate as a function of the number of epochs, where the results for sampling with fixed hubs is shown in gray. The starting and ending values are marked by 'x' symbols and the shaded region around the curves falls between the 40th and 60th percentiles.

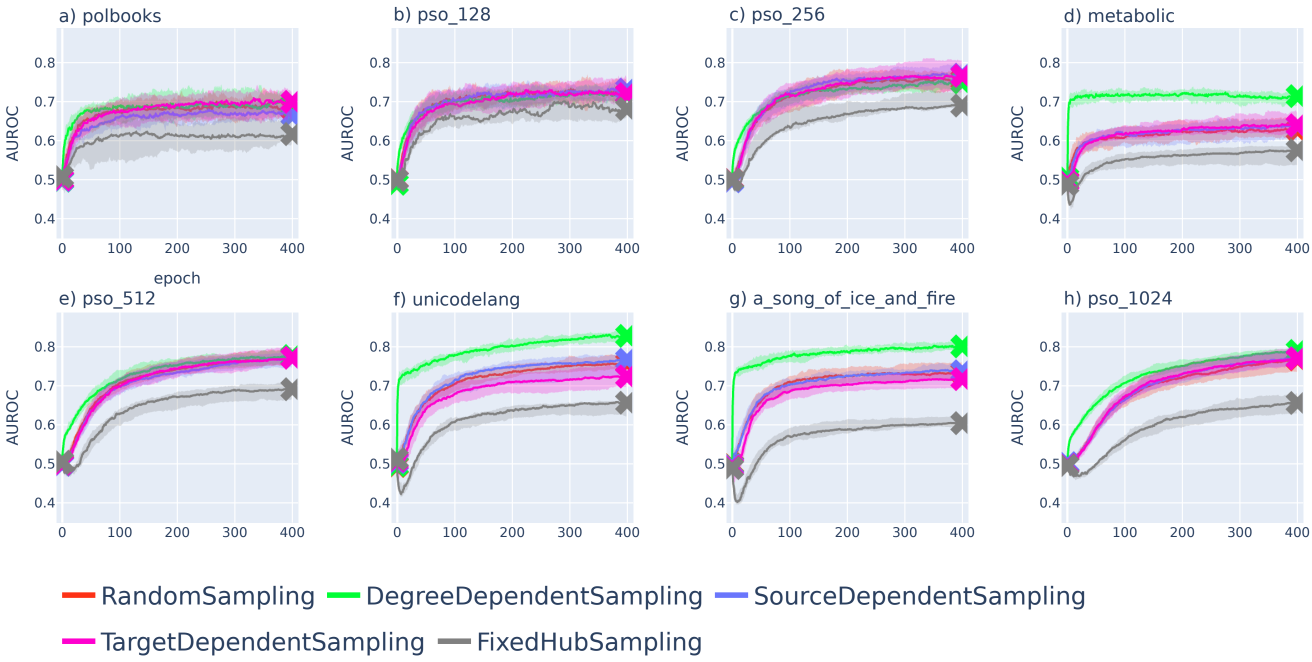

Figure S26. The AUROC for random embeddings with fixed hubs. We plot the median over 20 instances for the success rate as a function of the number of epochs, where the results for sampling with fixed hubs is shown in gray. The starting and ending values are marked by 'x' symbols and the shaded region around the curves falls between the 40th and 60th percentiles.

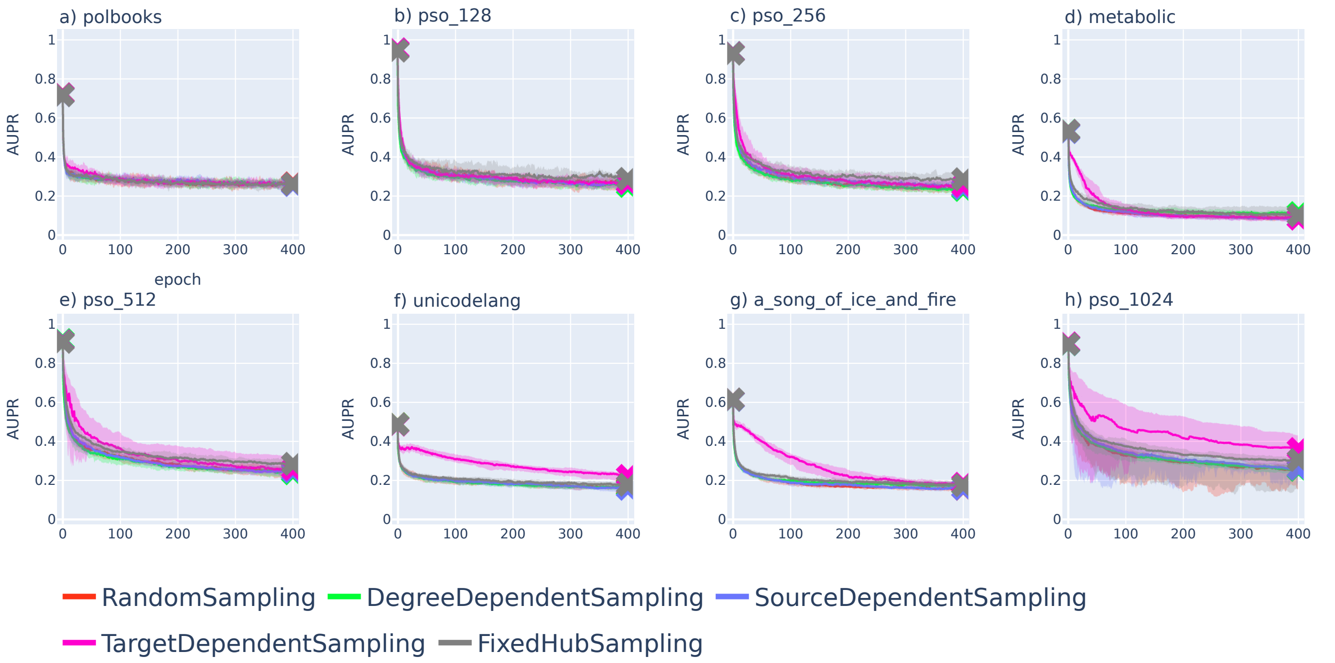

Figure S27. The AUPR for Mercator embeddings with fixed hubs. We plot the median over 20 instances for the success rate as a function of the number of epochs, where the results for sampling with fixed hubs is shown in gray. The starting and ending values are marked by 'x' symbols and the shaded region around the curves falls between the 40th and 60th percentiles.

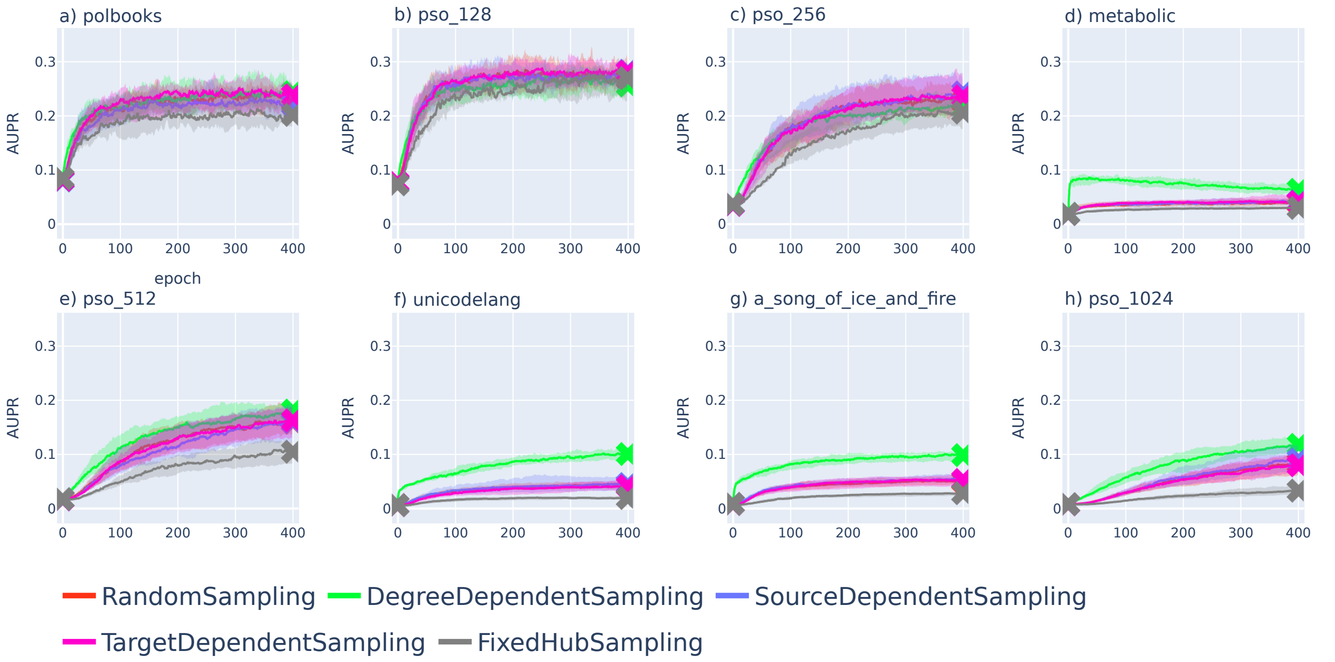

Figure S28. The AUPR for random embeddings with fixed hubs. We plot the median over 20 instances for the success rate as a function of the number of epochs, where the results for sampling with fixed hubs is shown in gray. The starting and ending values are marked by 'x' symbols and the shaded region around the curves falls between the 40th and 60th percentiles.

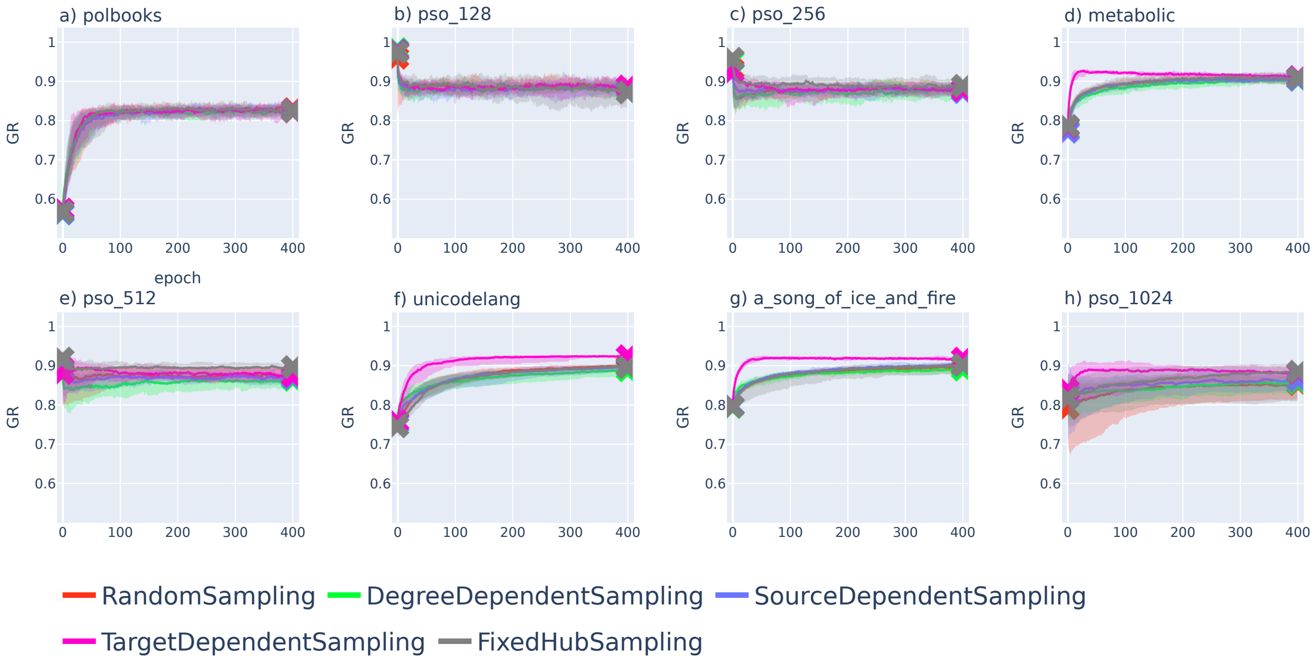

Figure S29. Change in the greedy routing score for Mercator embeddings with fixed hubs. We plot the median over 20 instances for the success rate as a function of the number of epochs, where the results for sampling with fixed hubs is shown in gray. The starting and ending values are marked by ’x’ symbols and the shaded region around the curves falls between the 40th and 60th percentiles.

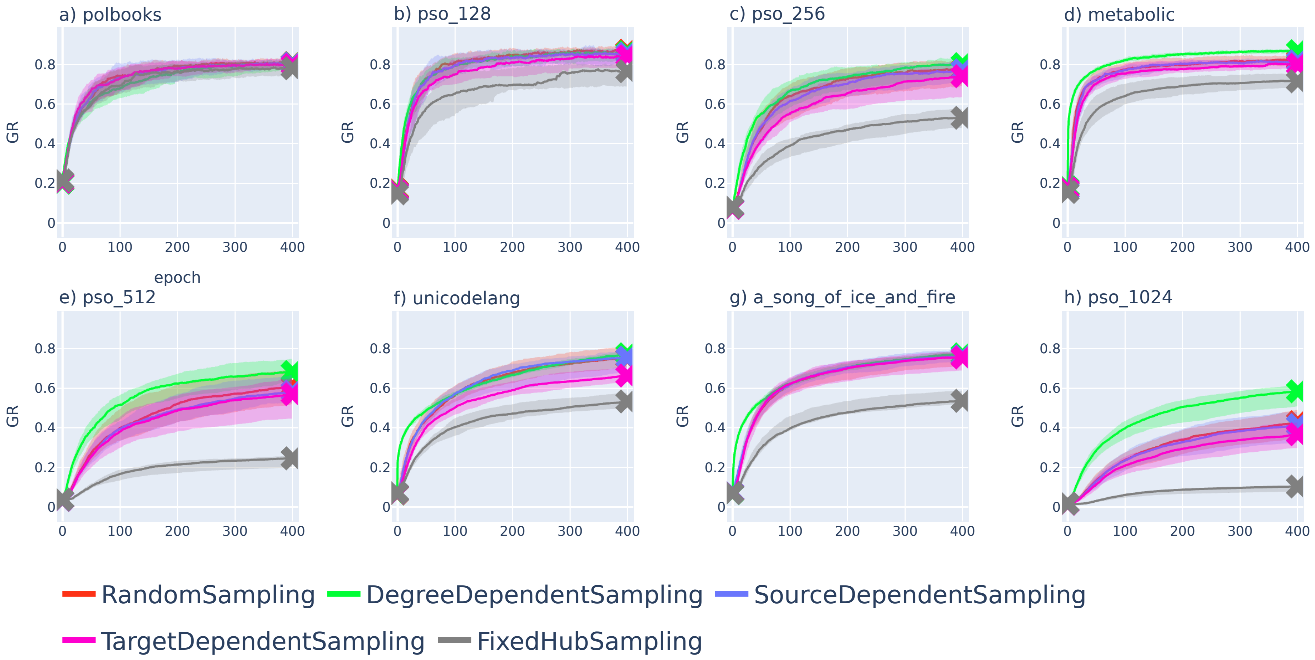

Figure S30. Change in the GR for random embeddings with fixed hubs. We plot the median over 20 instances for the success rate as a function of the number of epochs, where the results for sampling with fixed hubs is shown in gray. The starting and ending values are marked by ’x’ symbols and the shaded region around the curves falls between the 40th and 60th percentiles.

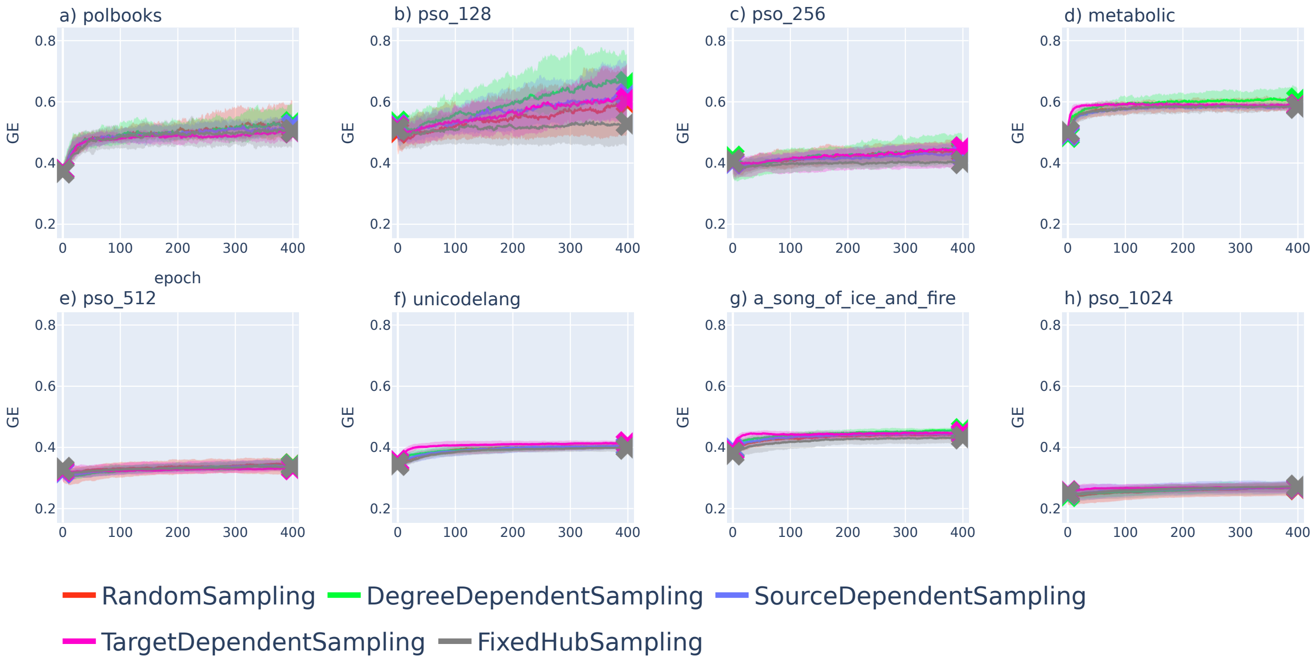

Figure S31. **Change in the greedy routing efficiency for Mercator embeddings with fixed hubs.** We plot the median over 20 instances for the success rate as a function of the number of epochs, where the results for sampling with fixed hubs is shown in gray. The starting and ending values are marked by 'x' symbols and the shaded region around the curves falls between the 40th and 60th percentiles.

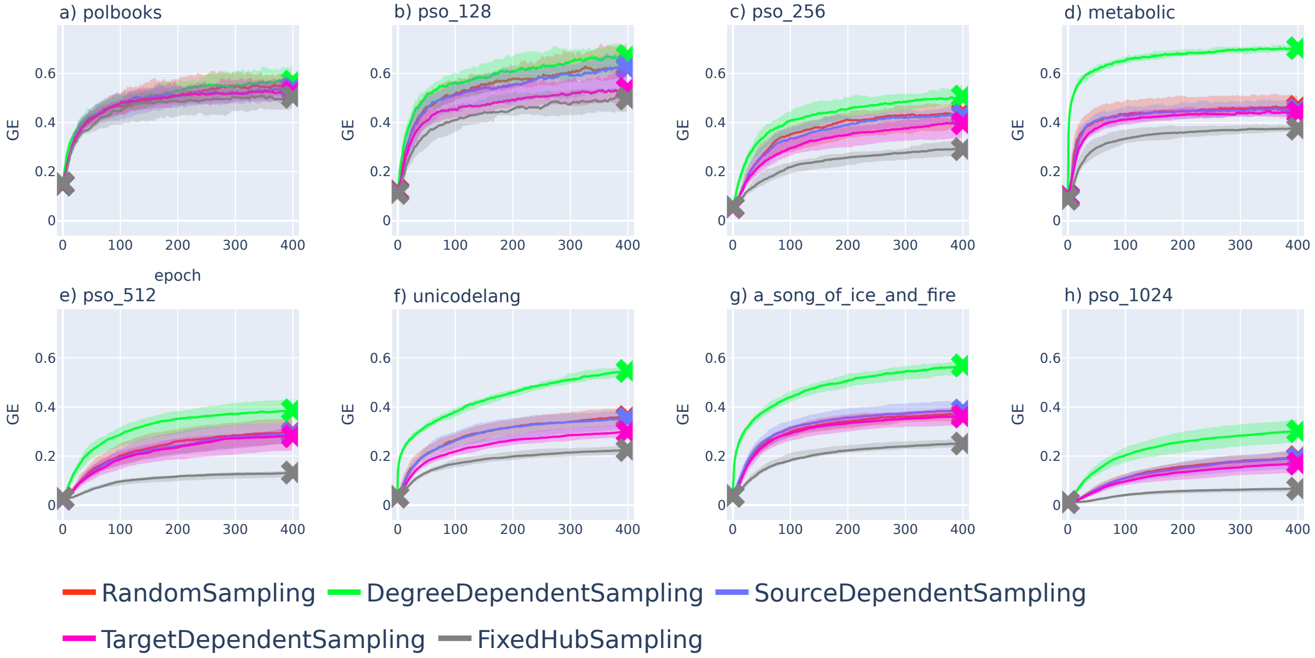

Figure S32. **Change in the GE for random embeddings with fixed hubs.** We plot the median over 20 instances for the success rate as a function of the number of epochs, where the results for sampling with fixed hubs is shown in gray. The starting and ending values are marked by 'x' symbols and the shaded region around the curves falls between the 40th and 60th percentiles.

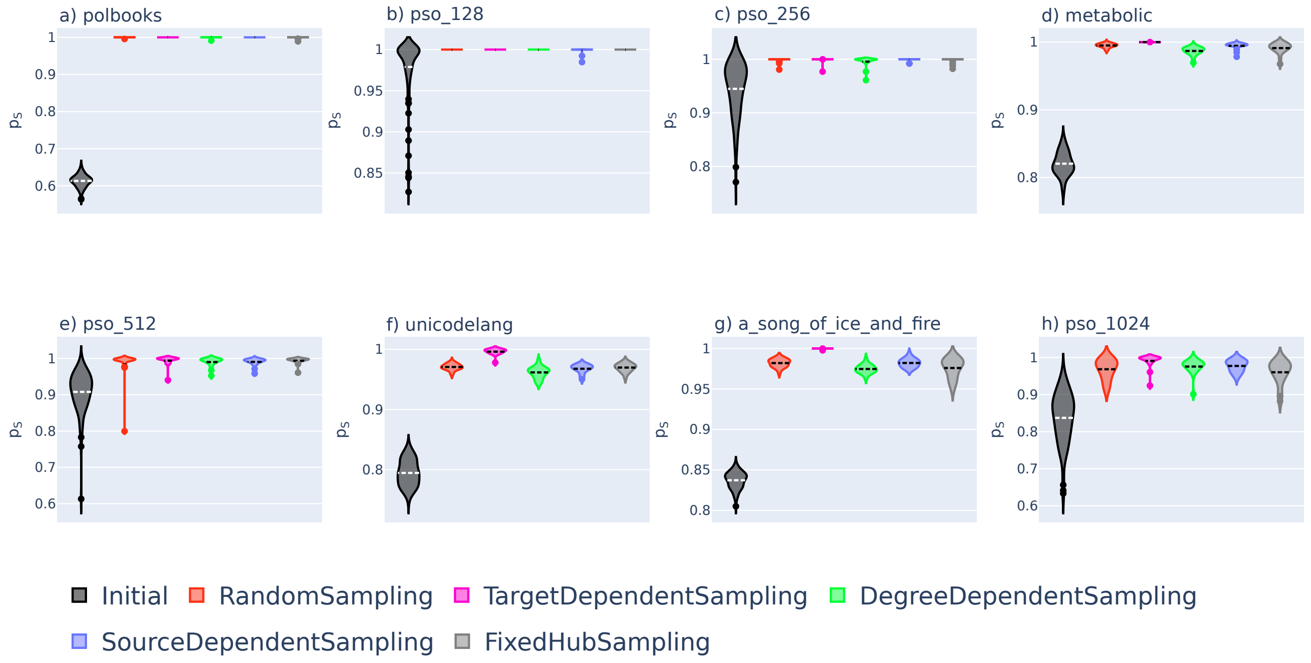

Figure S33. Violin plot for p_s at the end of the optimisation in the case of Mercator embeddings. We show the distribution of the p_s values at the end of the optimisation for random sampling (red), target dependent sampling (purple), degree dependent sampling (green), source dependent sampling (blue) and also for annealing with fixed hubs (grey). For comparison, the distribution before the optimisation is also shown in black.

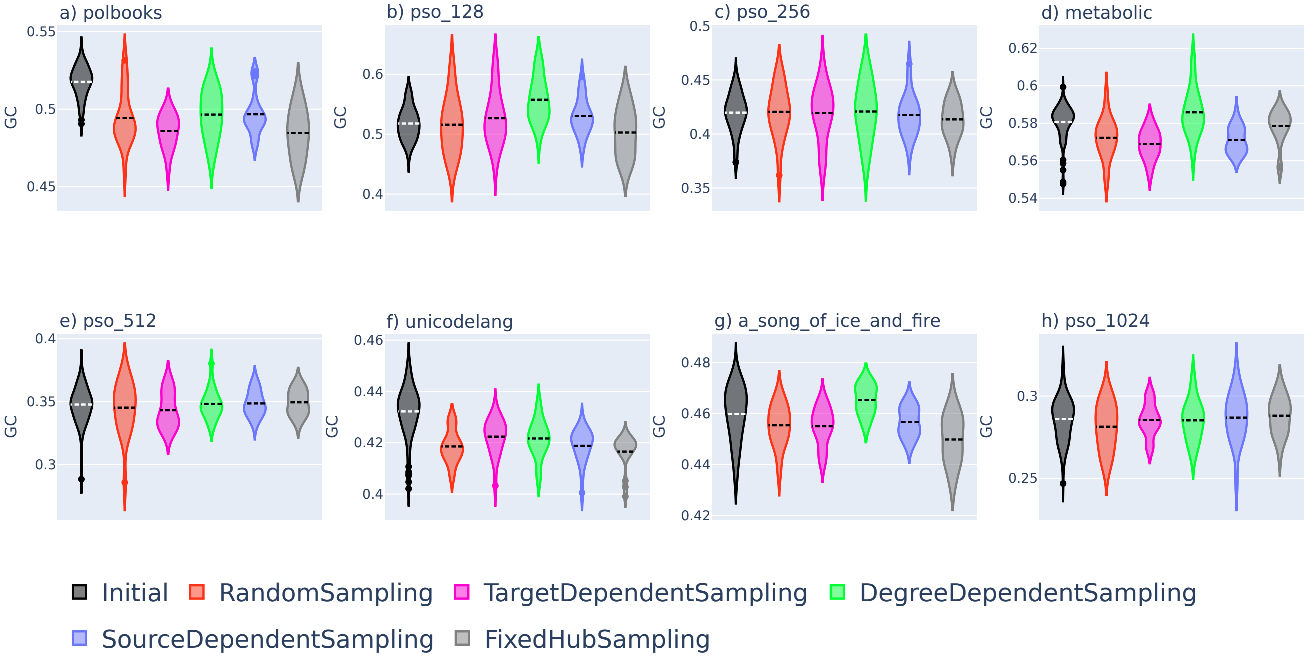

Figure S34. Violin plot for the geometrical congruence at the end of the optimisation in the case of Mercator embeddings. We show the distribution of the GC values at the end of the optimisation for random sampling (red), target dependent sampling (purple), degree dependent sampling (green), source dependent sampling (blue) and also for annealing with fixed hubs (grey). For comparison, the distribution before the optimisation is also shown in black.

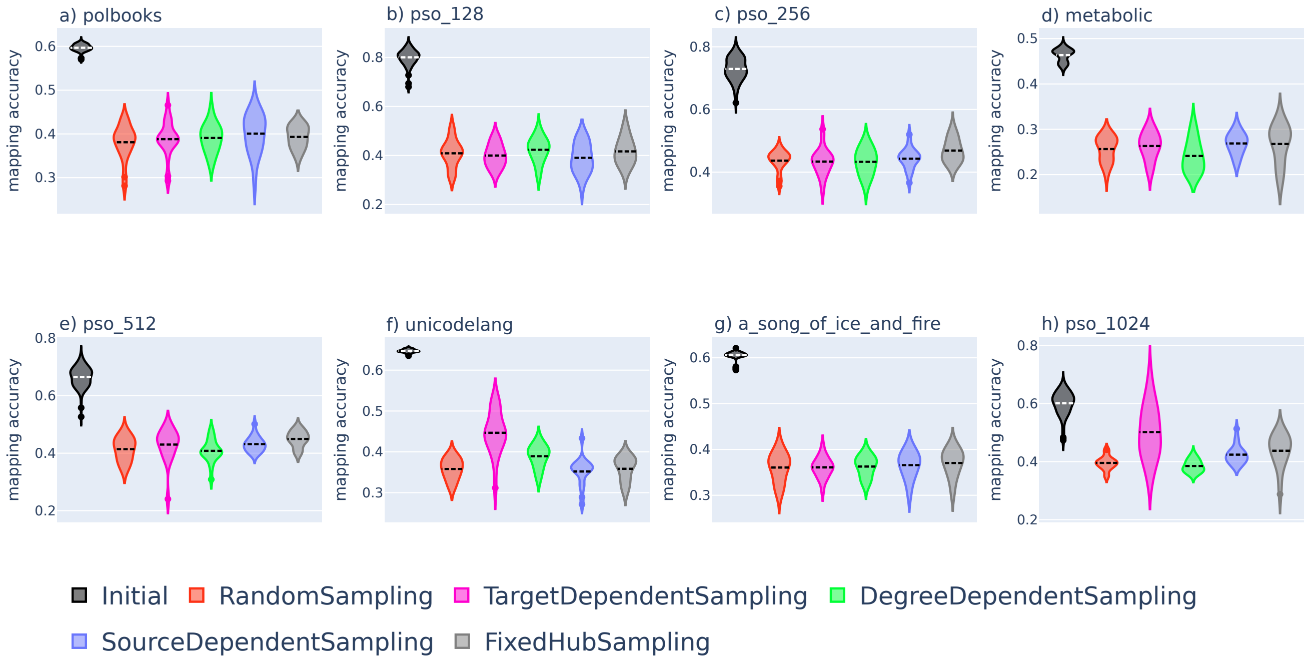

Figure S35. Violin plot for the mapping accuracy at the end of the optimisation in the case of Mercator embeddings. We show the distribution of the mapping accuracy values at the end of the optimisation for random sampling (red), target dependent sampling (purple), degree dependent sampling (green), source dependent sampling (blue) and also for annealing with fixed hubs (grey). For comparison, the distribution before the optimisation is also shown in black.

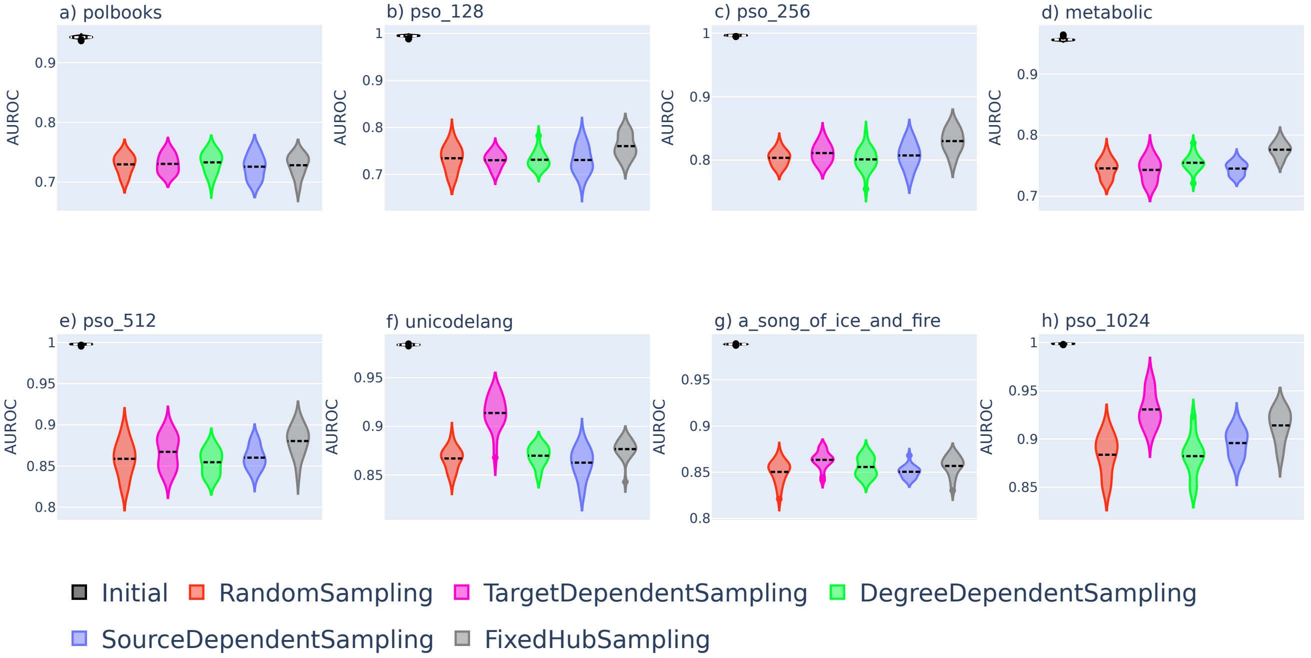

Figure S36. Violin plot for the AUROC at the end of the optimisation in the case of Mercator embeddings. We show the distribution of the AUROC values at the end of the optimisation for random sampling (red), target dependent sampling (purple), degree dependent sampling (green), source dependent sampling (blue) and also for annealing with fixed hubs (grey). For comparison, the distribution before the optimisation is also shown in black.

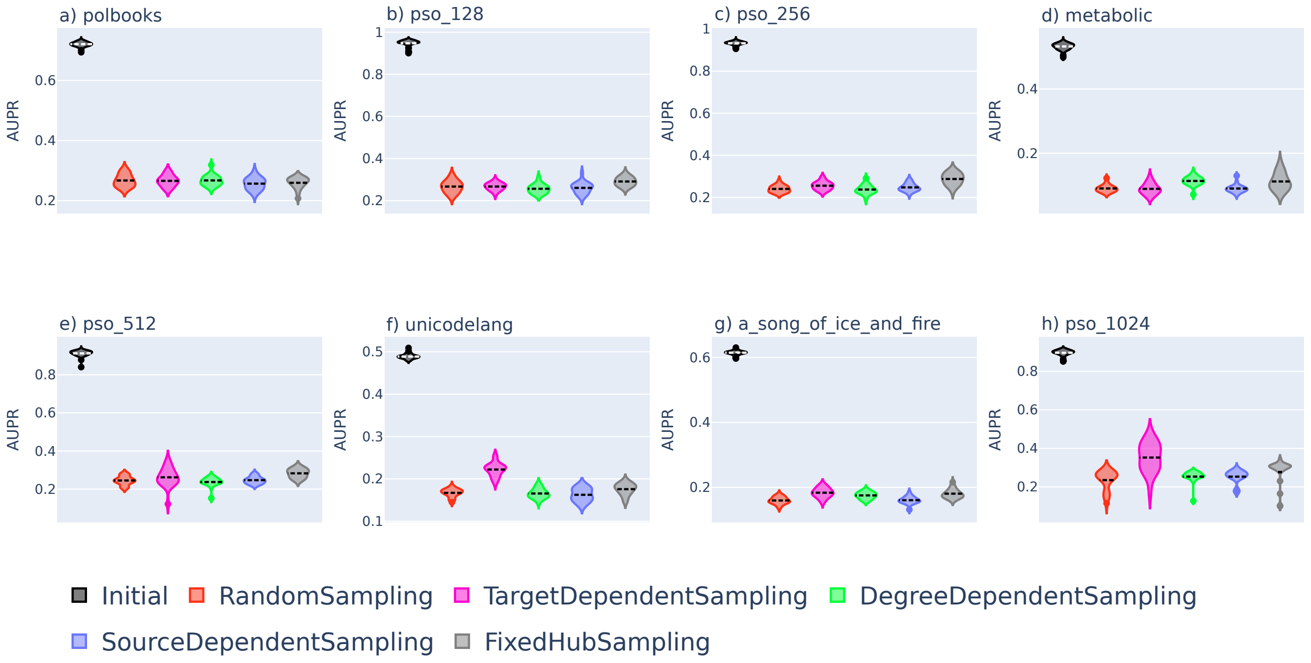

Figure S37. Violin plot for the AUPR at the end of the optimisation in the case of Mercator embeddings. We show the distribution of the AUPR values at the end of the optimisation for random sampling (red), target dependent sampling (purple), degree dependent sampling (green), source dependent sampling (blue) and also for annealing with fixed hubs (grey). For comparison, the distribution before the optimisation is also shown in black.

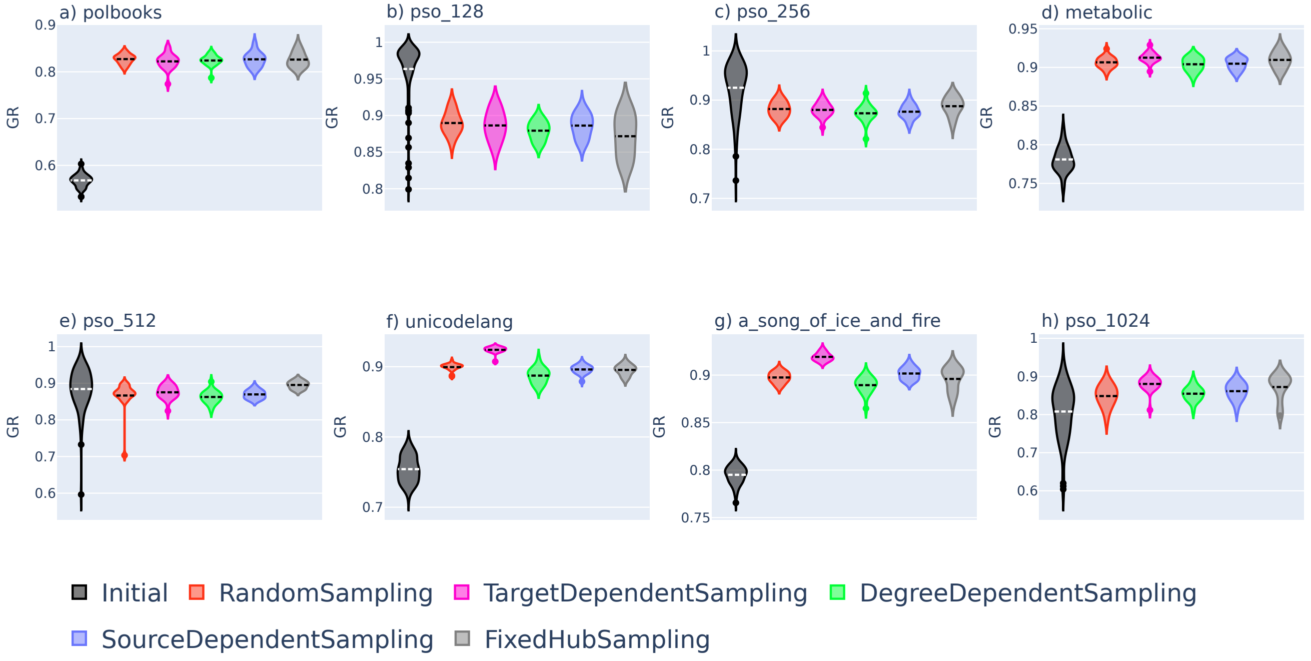

Figure S38. Violin plot for the greedy routing score at the end of the optimisation in the case of Mercator embeddings. We show the distribution of the GR values at the end of the optimisation for random sampling (red), target dependent sampling (purple), degree dependent sampling (green), source dependent sampling (blue) and also for annealing with fixed hubs (grey). For comparison, the distribution before the optimisation is also shown in black.

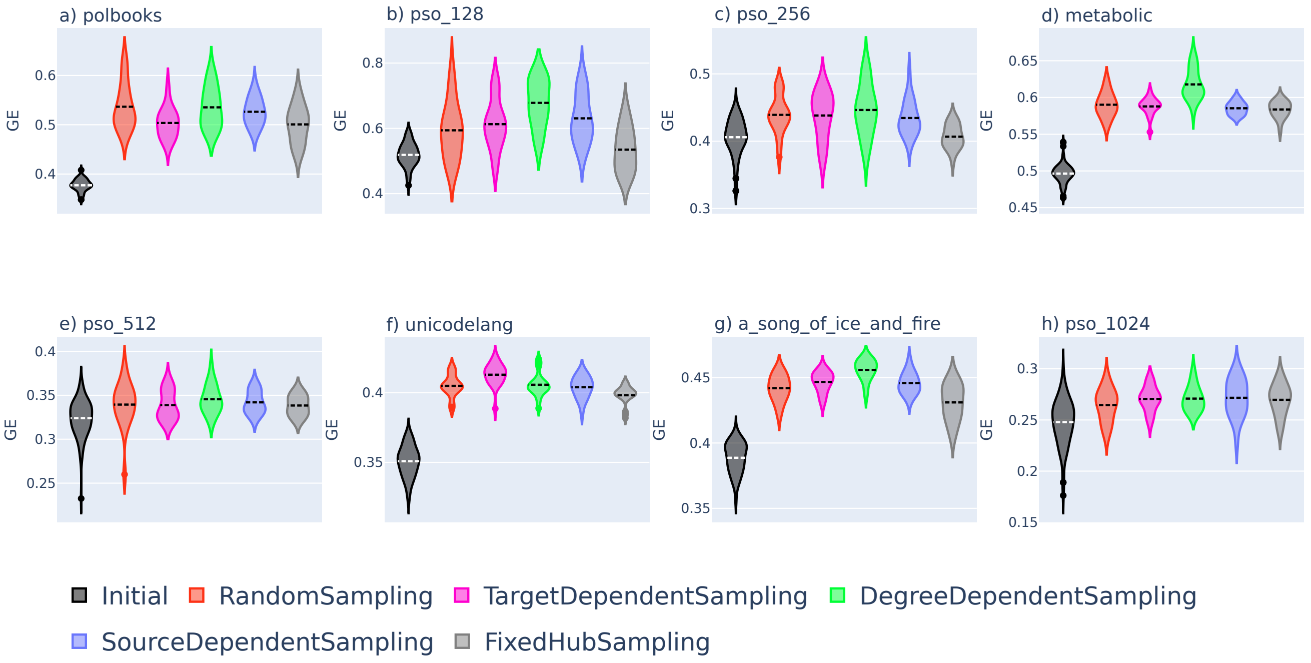

Figure S39. Violin plot for the greedy routing efficiency at the end of the optimisation in the case of Mercator embeddings. We show the distribution of the GE values at the end of the optimisation for random sampling (red), target dependent sampling (purple), degree dependent sampling (green), source dependent sampling (blue) and also for annealing with fixed hubs (grey). For comparison, the distribution before the optimisation is also shown in black.

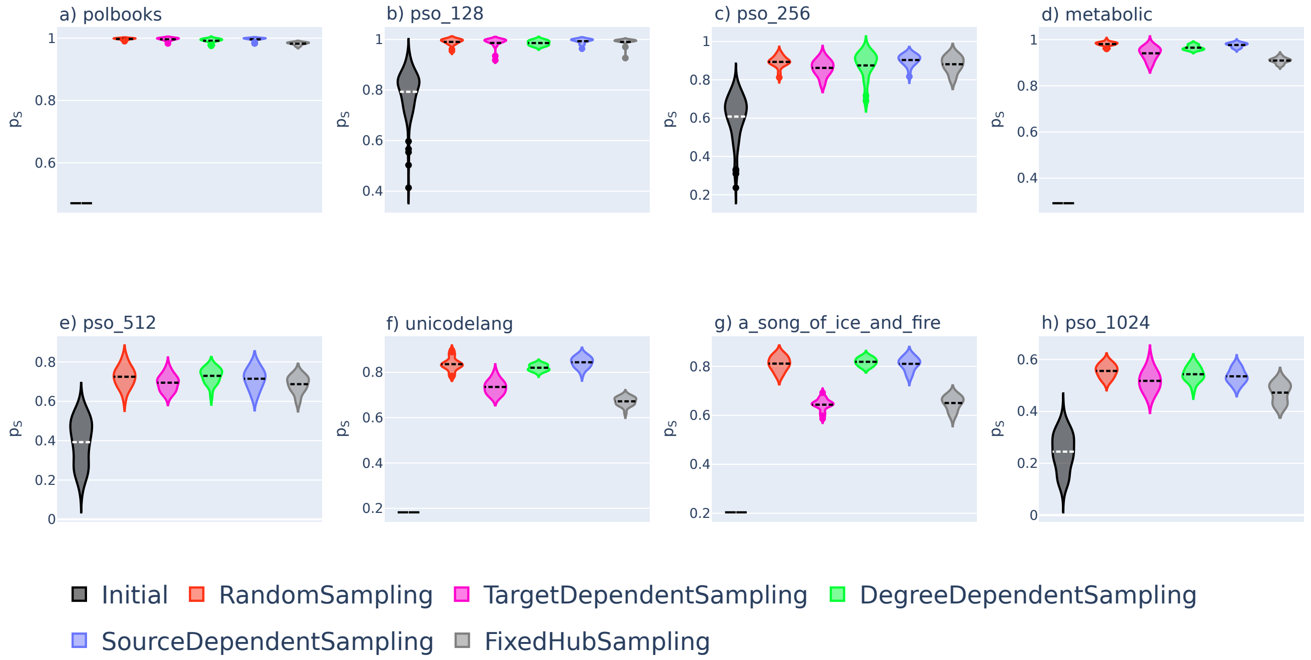

Figure S40. Violin plot for p_s at the end of the optimisation in the case of hyperbolic ISOMAP embeddings. We show the distribution of the p_s values at the end of the optimisation for random sampling (red), target dependent sampling (purple), degree dependent sampling (green), source dependent sampling (blue) and also for annealing with fixed hubs (grey). For comparison, the distribution before the optimisation is also shown in black.

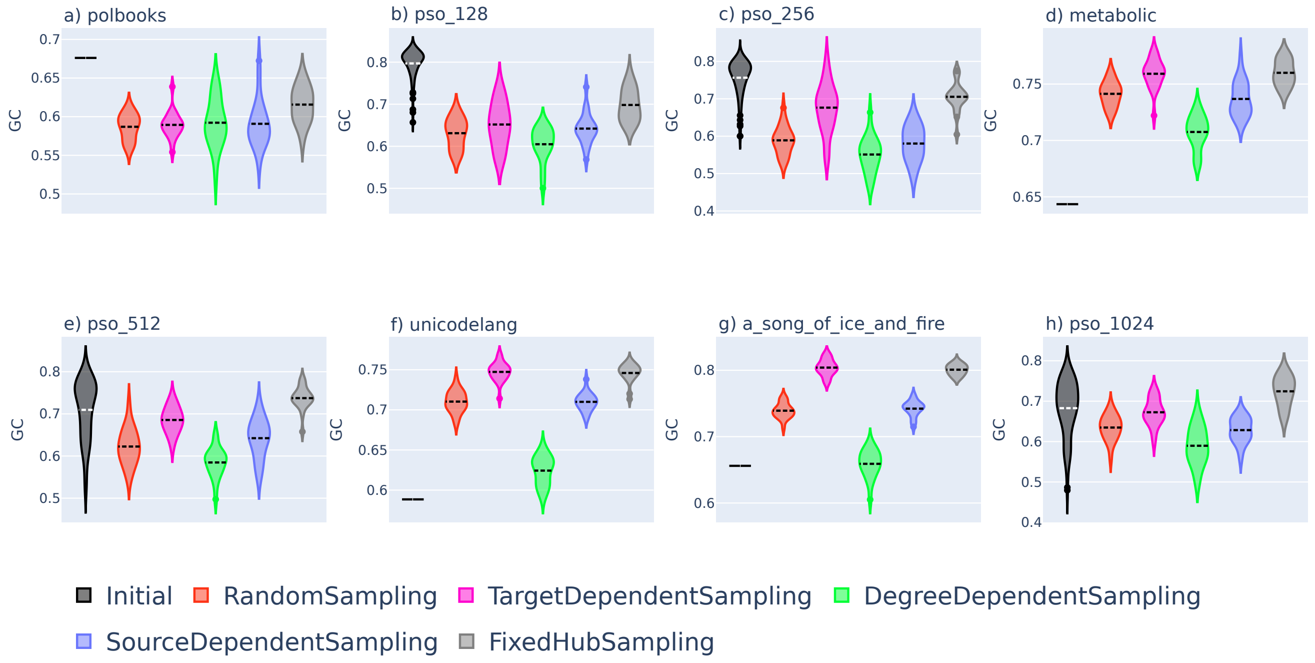

Figure S41. Violin plot for the geometrical congruence at the end of the optimisation in the case of hyperbolic ISOMAP embeddings. We show the distribution of the GC values at the end of the optimisation for random sampling (red), target dependent sampling (purple), degree dependent sampling (green), source dependent sampling (blue) and also for annealing with fixed hubs (grey). For comparison, the distribution before the optimisation is also shown in black.

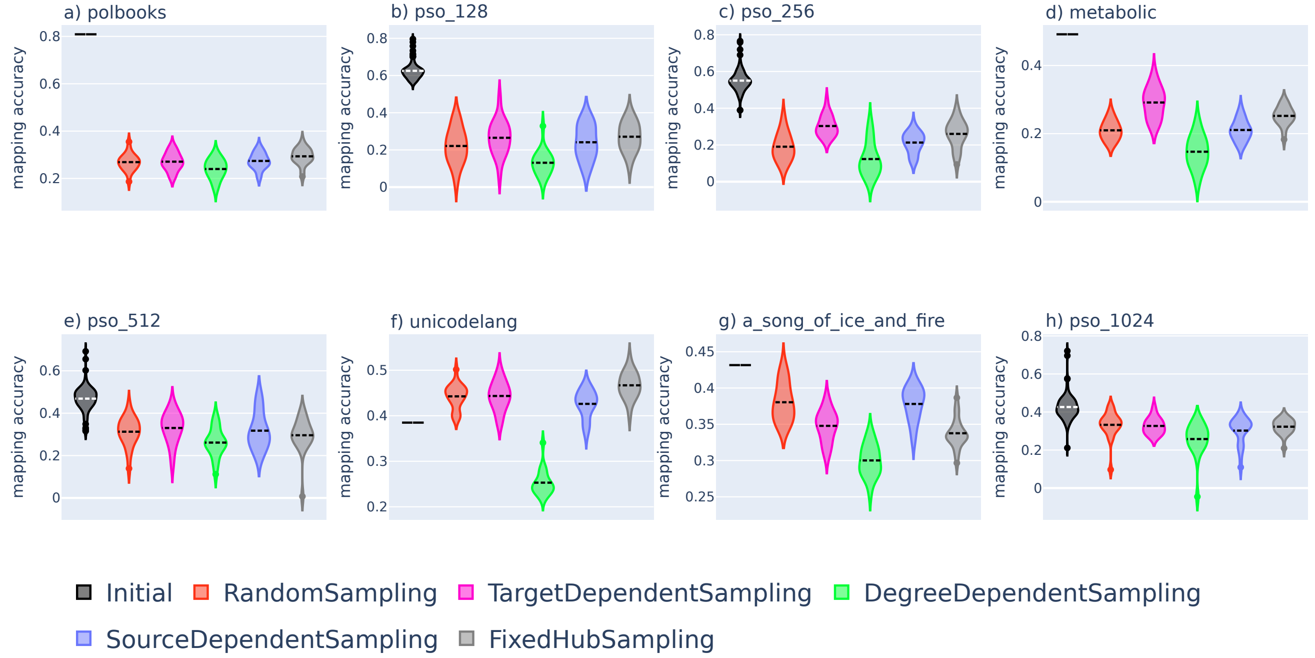

Figure S42. Violin plot for the mapping accuracy at the end of the optimisation in the case of hyperbolic ISOMAP embeddings. We show the distribution of the mapping accuracy values at the end of the optimisation for random sampling (red), target dependent sampling (purple), degree dependent sampling (green), source dependent sampling (blue) and also for annealing with fixed hubs (grey). For comparison, the distribution before the optimisation is also shown in black.

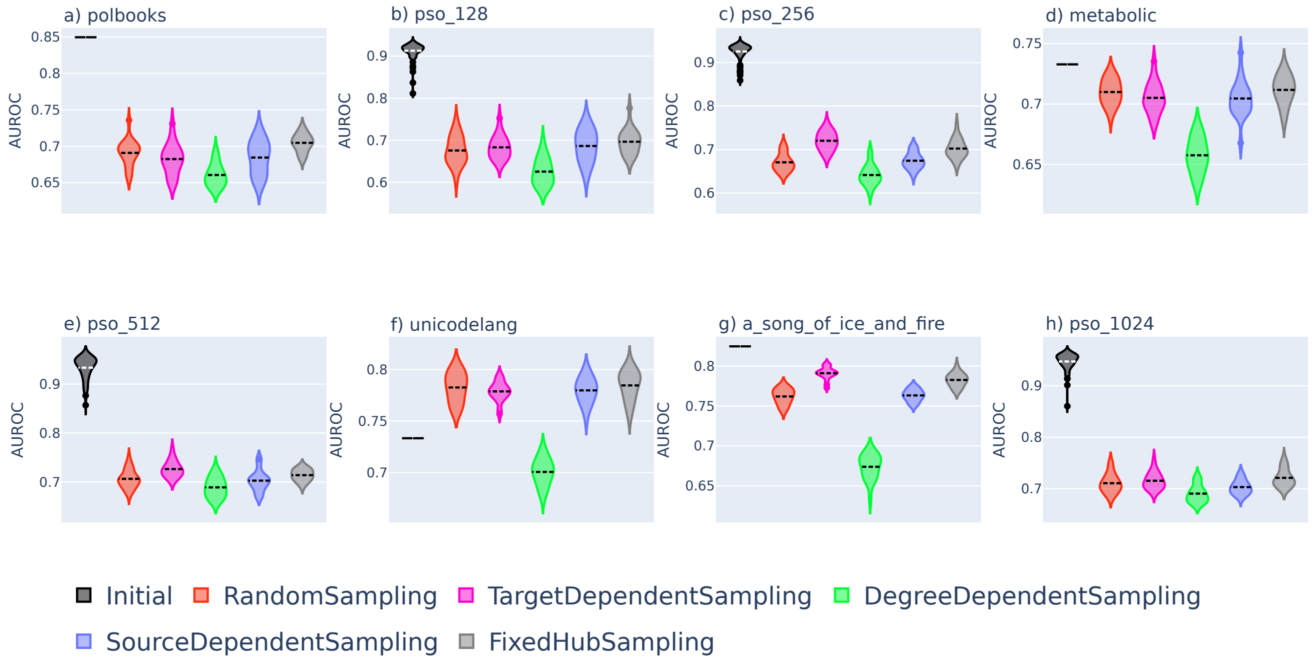

Figure S43. Violin plot for the AUROC at the end of the optimisation in the case of hyperbolic ISOMAP embeddings. We show the distribution of the AUROC values at the end of the optimisation for random sampling (red), target dependent sampling (purple), degree dependent sampling (green), source dependent sampling (blue) and also for annealing with fixed hubs (grey). For comparison, the distribution before the optimisation is also shown in black.

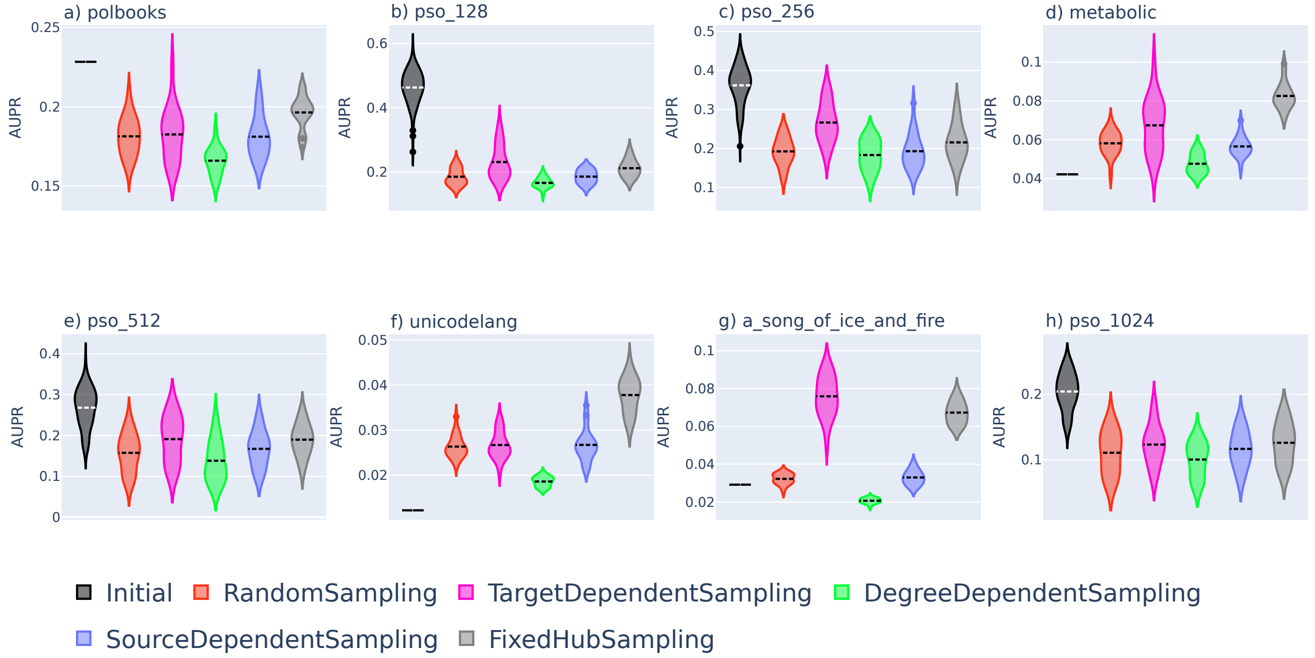

Figure S44. Violin plot for the AUPR at the end of the optimisation in the case of hyperbolic ISOMAP embeddings. We show the distribution of the AUPR values at the end of the optimisation for random sampling (red), target dependent sampling (purple), degree dependent sampling (green), source dependent sampling (blue) and also for annealing with fixed hubs (grey). For comparison, the distribution before the optimisation is also shown in black.

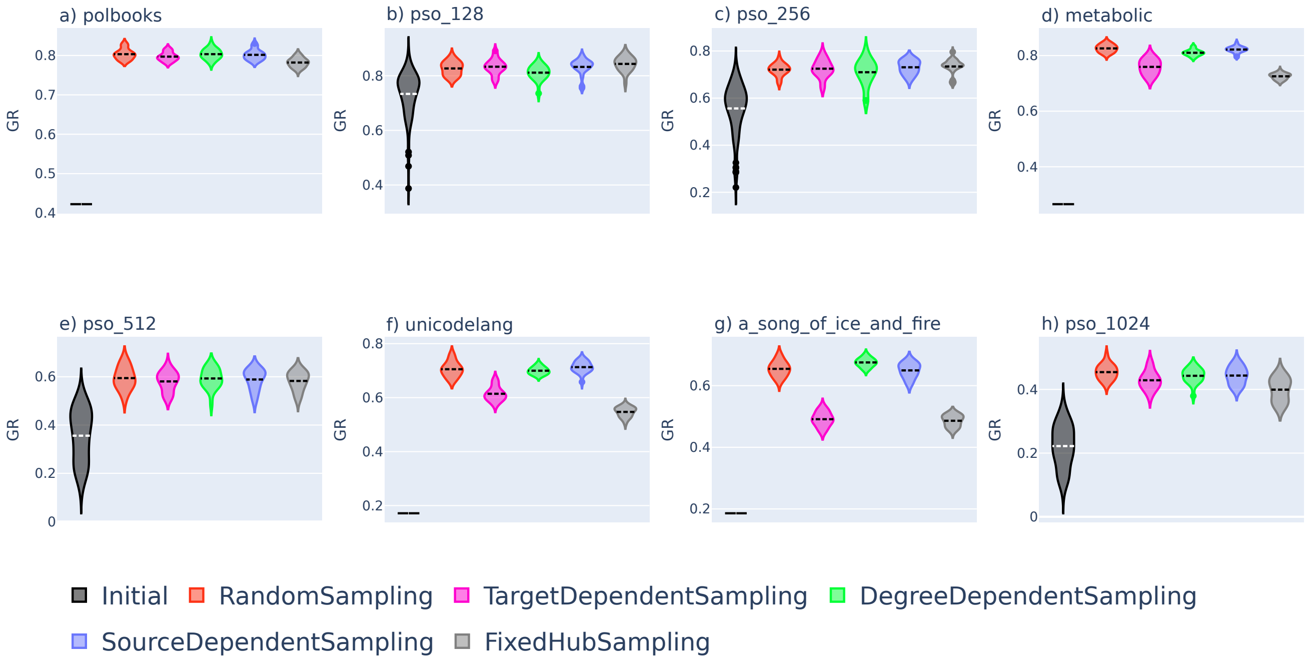

Figure S45. Violin plot for the greedy routing score at the end of the optimisation in the case of hyperbolic ISOMAP embeddings. We show the distribution of the GR values at the end of the optimisation for random sampling (red), target dependent sampling (purple), degree dependent sampling (green), source dependent sampling (blue) and also for annealing with fixed hubs (grey). For comparison, the distribution before the optimisation is also shown in black.

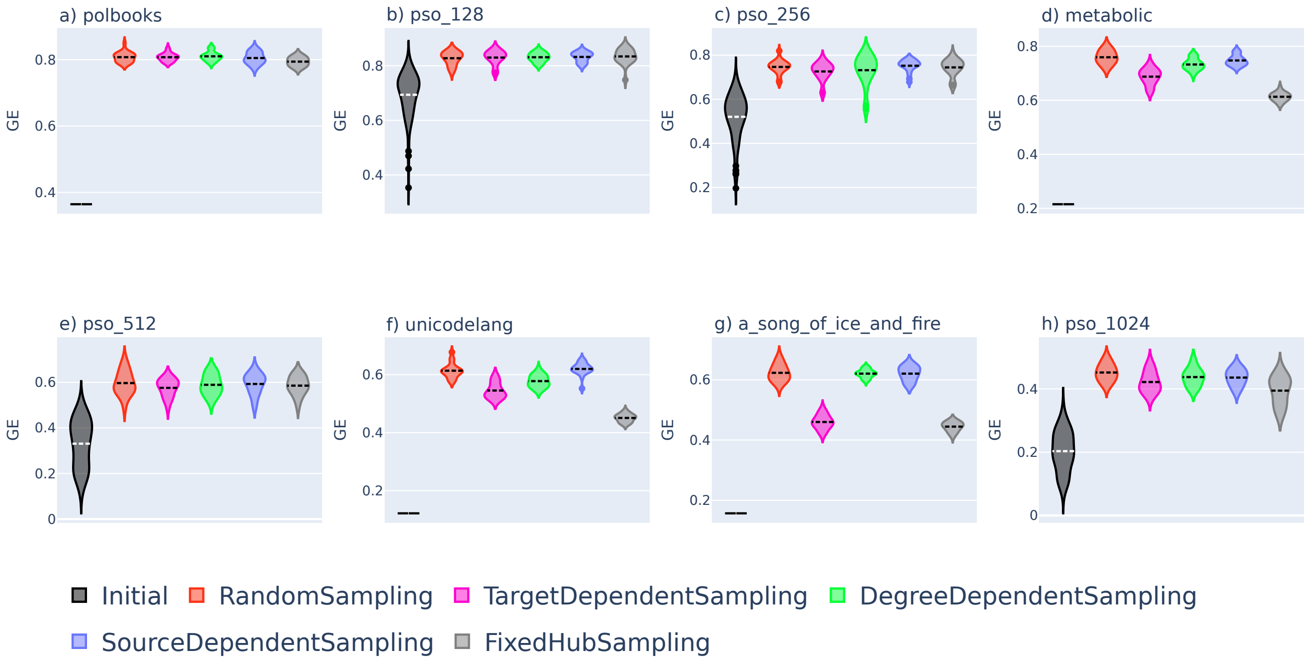

Figure S46. Violin plot for the greedy routing efficiency at the end of the optimisation in the case of hyperbolic ISOMAP embeddings. We show the distribution of the GE values at the end of the optimisation for random sampling (red), target dependent sampling (purple), degree dependent sampling (green), source dependent sampling (blue) and also for annealing with fixed hubs (grey). For comparison, the distribution before the optimisation is also shown in black.

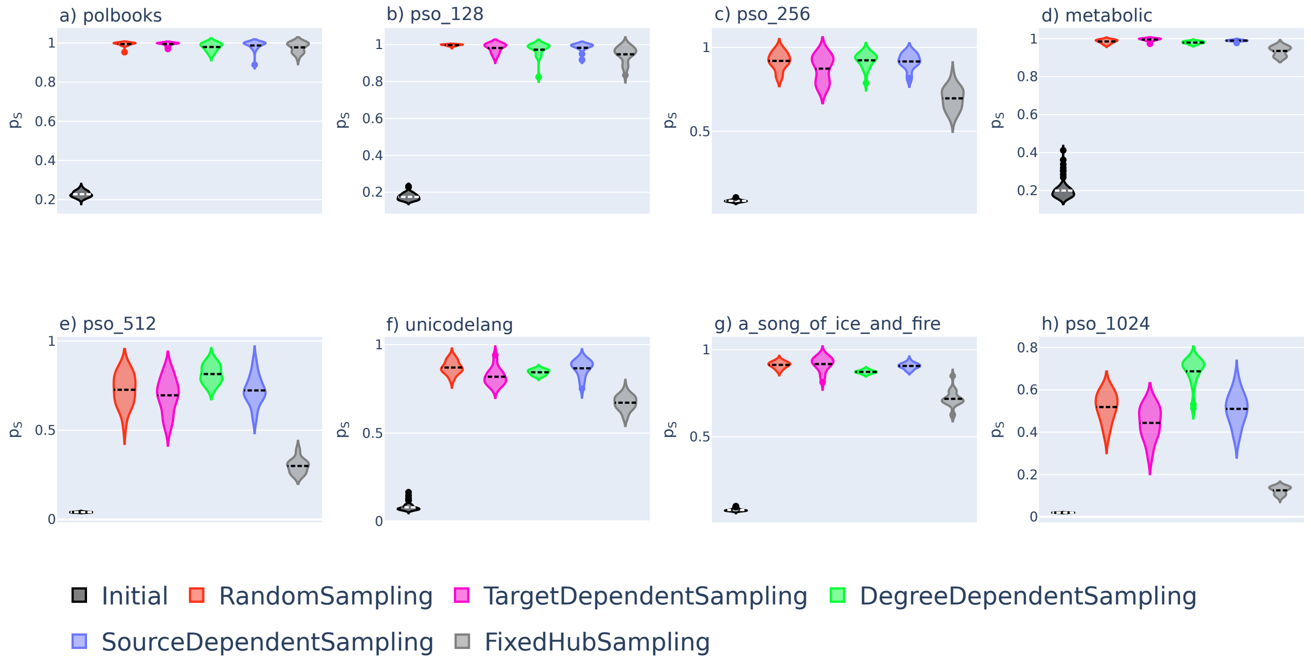

Figure S47. **Violin plot for p_s at the end of the optimisation in the case of random embeddings.** We show the distribution of the p_s values at the end of the optimisation for random sampling (red), target dependent sampling (purple), degree dependent sampling (green), source dependent sampling (blue) and also for annealing with fixed hubs (grey). For comparison, the distribution before the optimisation is also shown in black.

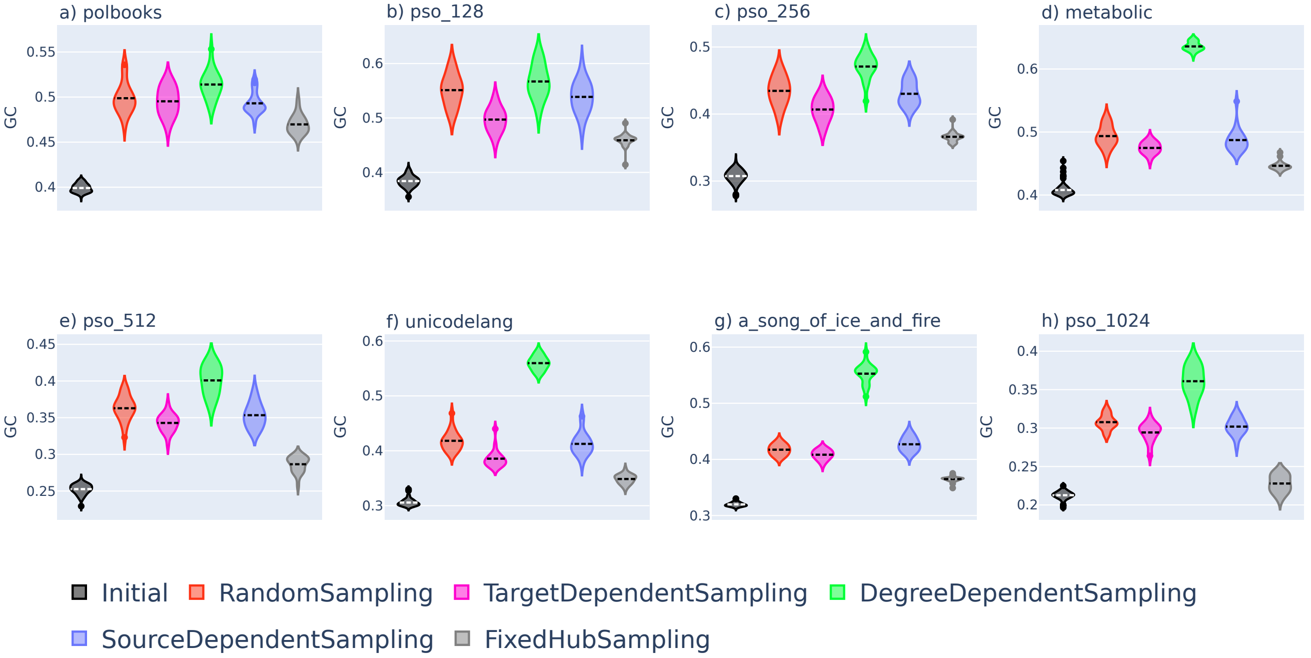

Figure S48. **Violin plot for the geometrical congruence at the end of the optimisation in the case of random embeddings.** We show the distribution of the GC values at the end of the optimisation for random sampling (red), target dependent sampling (purple), degree dependent sampling (green), source dependent sampling (blue) and also for annealing with fixed hubs (grey). For comparison, the distribution before the optimisation is also shown in black.

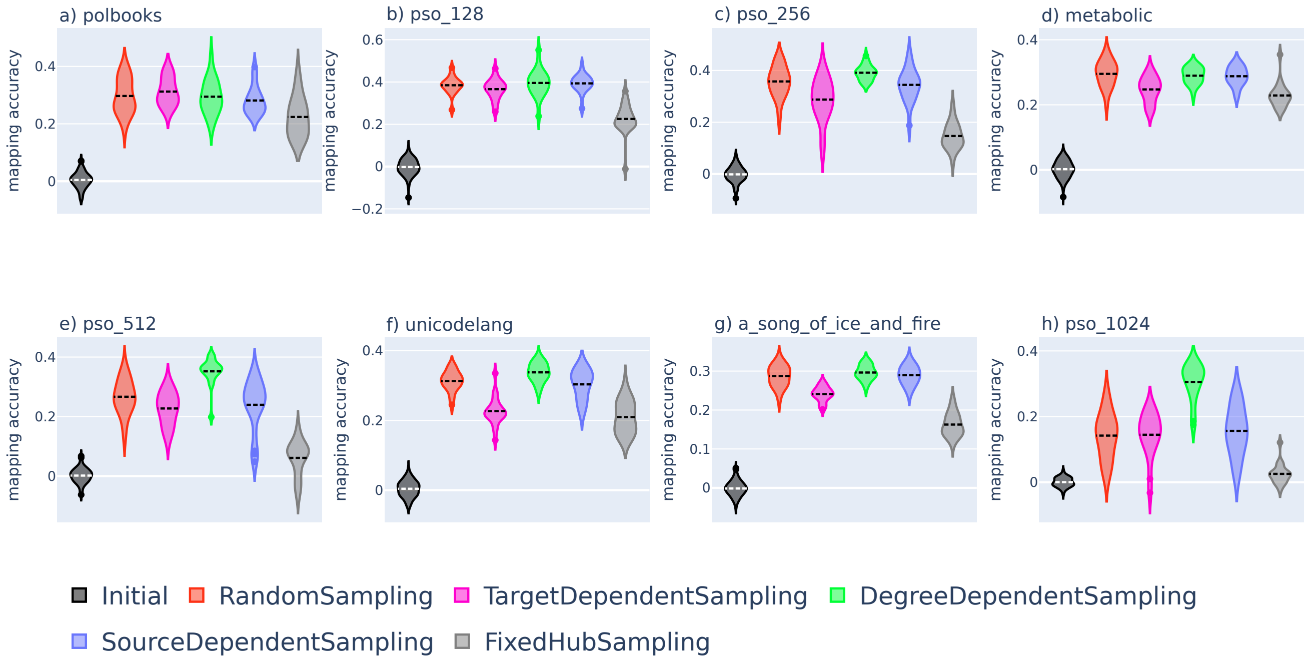

Figure S49. Violin plot for the mapping accuracy at the end of the optimisation in the case of random embeddings. We show the distribution of the mapping accuracy values at the end of the optimisation for random sampling (red), target dependent sampling (purple), degree dependent sampling (green), source dependent sampling (blue) and also for annealing with fixed hubs (grey). For comparison, the distribution before the optimisation is also shown in black.

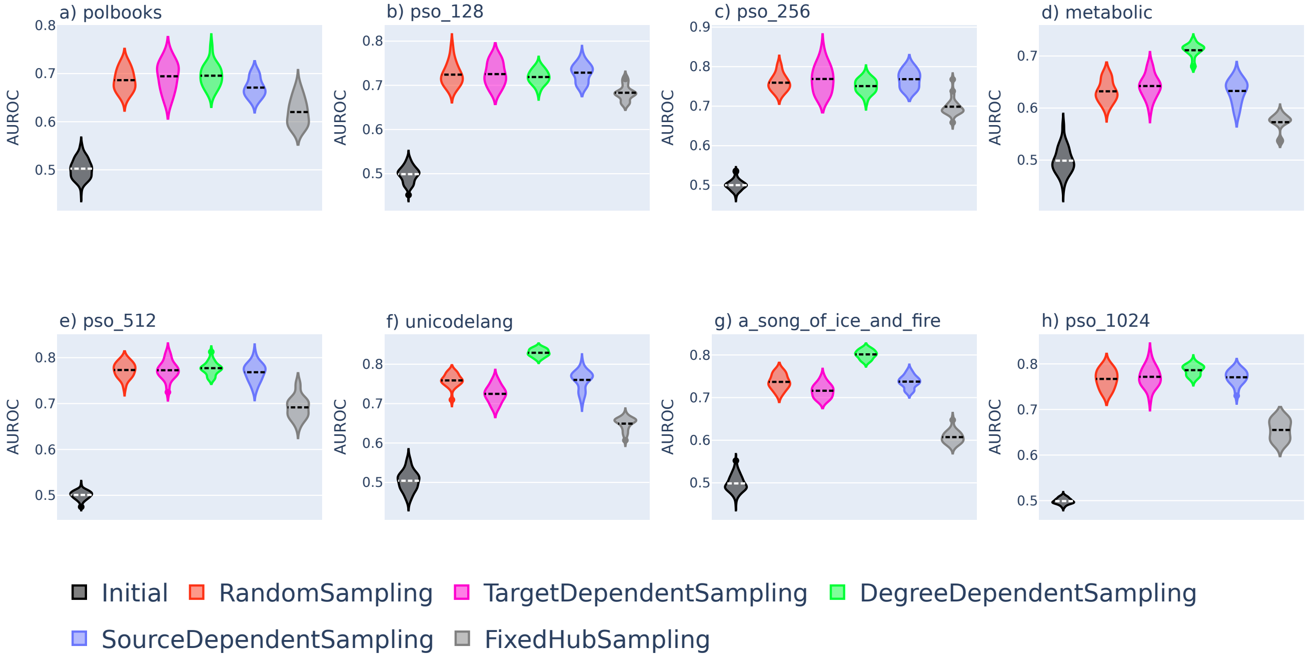

Figure S50. Violin plot for the AUROC at the end of the optimisation in the case of random embeddings. We show the distribution of the AUROC values at the end of the optimisation for random sampling (red), target dependent sampling (purple), degree dependent sampling (green), source dependent sampling (blue) and also for annealing with fixed hubs (grey). For comparison, the distribution before the optimisation is also shown in black.

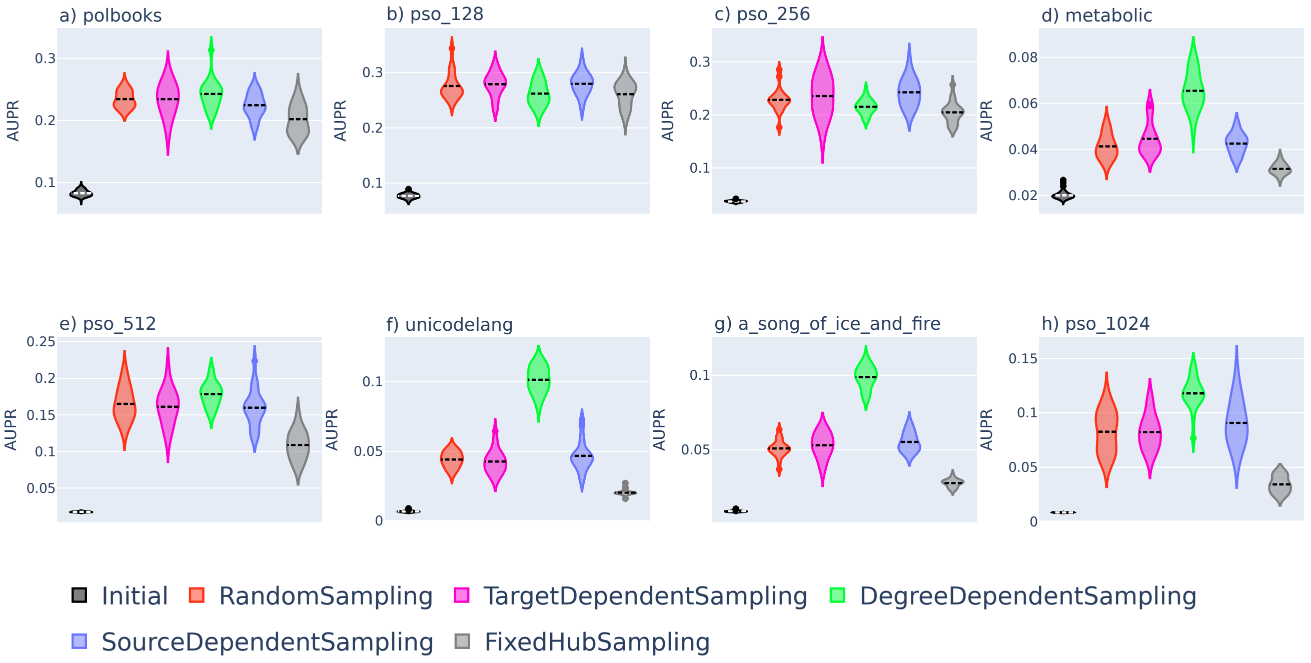

Figure S51. Violin plot for the AUPR at the end of the optimisation in the case of random embeddings. We show the distribution of the AUPR values at the end of the optimisation for random sampling (red), target dependent sampling (purple), degree dependent sampling (green), source dependent sampling (blue) and also for annealing with fixed hubs (grey). For comparison, the distribution before the optimisation is also shown in black.

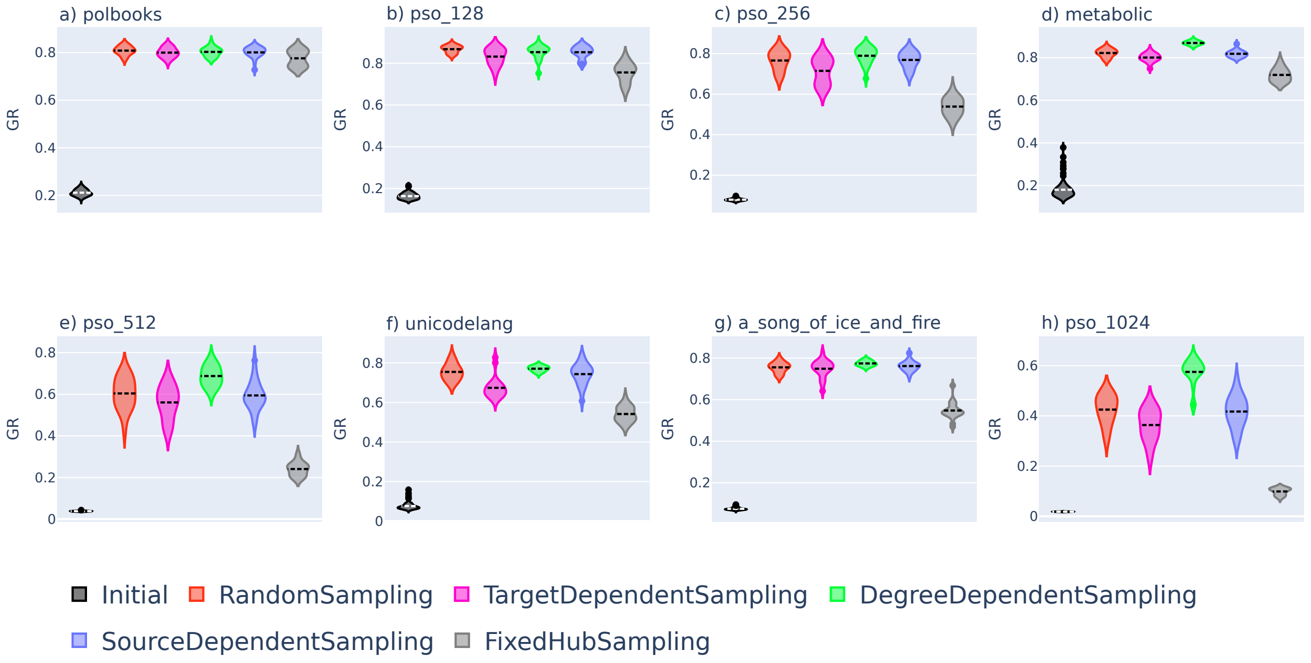

Figure S52. Violin plot for the greedy routing score at the end of the optimisation in the case of random embeddings. We show the distribution of the GR values at the end of the optimisation for random sampling (red), target dependent sampling (purple), degree dependent sampling (green), source dependent sampling (blue) and also for annealing with fixed hubs (grey). For comparison, the distribution before the optimisation is also shown in black.

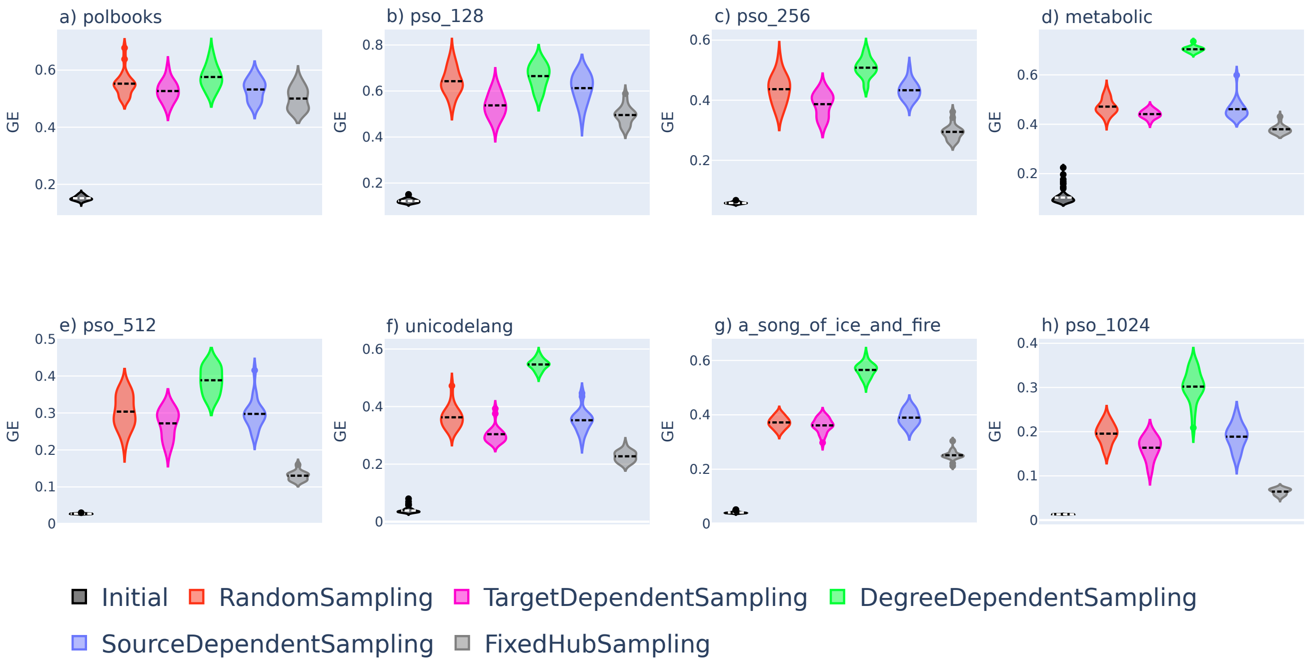

Figure S53. Violin plot for the greedy routing efficiency at the end of the optimisation in the case of random embeddings. We show the distribution of the GE values at the end of the optimisation for random sampling (red), target dependent sampling (purple), degree dependent sampling (green), source dependent sampling (blue) and also for annealing with fixed hubs (grey). For comparison, the distribution before the optimisation is also shown in black.

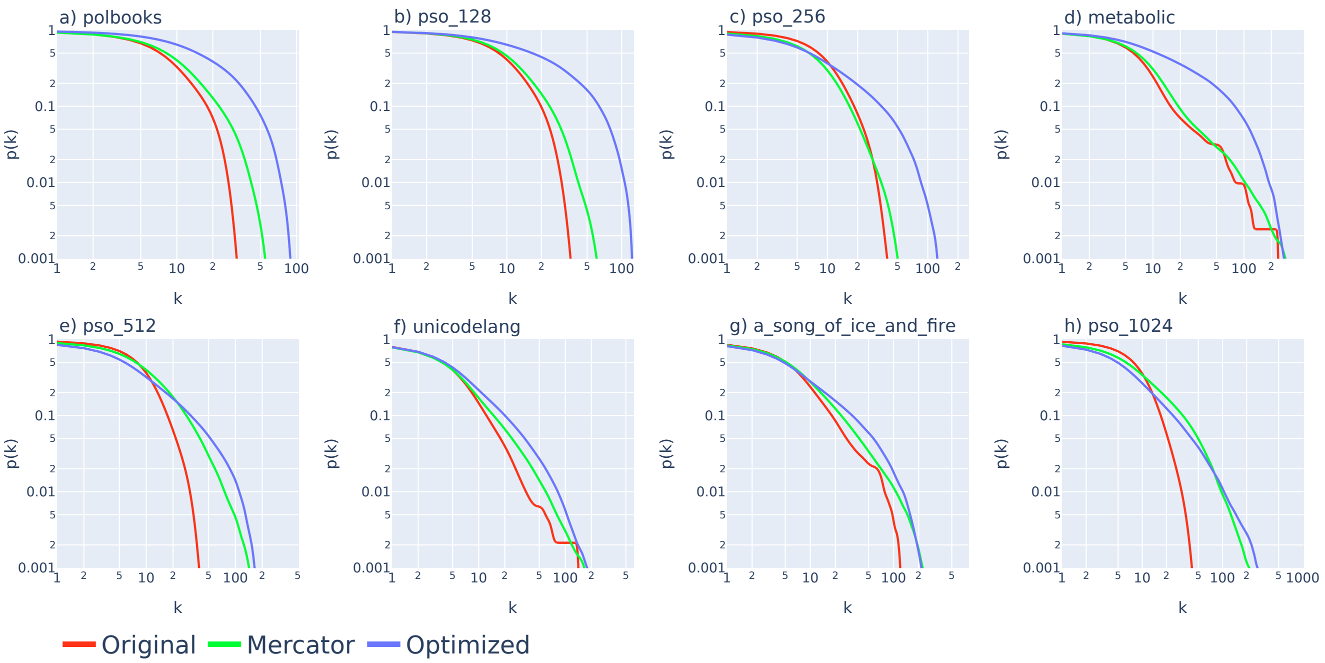

Figure S54. The degree distribuion of the original networks compared to that of graphs obtained in the graph-reconstructon processes. We show the complementary cumulative distribution $p(k)$ as a function of the node degree in red for the original networks, in green for the reconstructed networks based on the Mercator embedding before the optimisation, in blue for the reconstructed networks based on the optimised embeddings and in magenta for reconstruction graphs based on random embeddings.

network name	$\langle C \rangle$ in orignal net	$\langle C \rangle$ in Mercator rec.	$\langle C \rangle$ in optimised rec.
polbooks	0.488 ± 0.0	0.457 ± 0.065	0.564 ± 0.108
pso_128	0.685 ± 0.012	0.663 ± 0.032	0.727 ± 0.118
pso_256	0.675 ± 0.012	0.589 ± 0.100	0.534 ± 0.142
metabolic	0.646 ± 0.0	0.548 ± 0.114	0.604 ± 0.132
pso_512	0.670 ± 0.007	0.552 ± 0.167	0.478 ± 0.192
unicodelang	0.083 ± 0.0	0.149 ± 0.129	0.154 ± 0.123
a_song_of_ice_and_fire	0.486 ± 0.0	0.389 ± 0.209	0.367 ± 0.187
pso_1024	0.669 ± 0.007	0.483 ± 0.217	0.429 ± 0.221

Table S1. The average clustering coefficient in the original and reconstructed networks. The 1st column lists the studied networks, the 2nd column provides the average clustering coefficient, $\langle C \rangle$ in the orignal network, the 3rd column provides $\langle C \rangle$ in the reconstructed network based on the Mercator embedding, and finally, the 4th column lists $\langle C \rangle$ in the reconstructed network based on the optimised coordinates. The average and the standard deviation (displayed aside the average in each cell) was calculated over 20 instances for the original PSO networks and over 20 embeddings in the case of reconstructed networks.
